# Spinal manipulation and mobilisation in the treatment of infants, children, and adolescents: a systematic scoping review

**DOI:** 10.1186/s12887-022-03781-6

**Published:** 2022-12-19

**Authors:** Nikki Milne, Lauren Longeri, Anokhi Patel, Jan Pool, Kenneth Olson, Annalie Basson, Anita R. Gross

**Affiliations:** 1 Department of Physiotherapy, Faculty of Health Sciences and Medicine, Bond University, Queensland, Australia; 2 International Organisation of Physiotherapists in Paediatrics, World Physiotherapy Subgroup, Queensland, Australia; 3 Research Group Lifestyle and Health, Institute of Human Movement Studies, University of Applied Sciences, Utrecht, The Netherlands; 4 International Federation of Orthopaedic Manipulative Physical Therapy and Northern Rehab Physical Therapy Specialists, Anchorage, USA; 5 University of Witwatersrand, Johannesburg, South Africa; 6 McMaster University, Hamilton, Canada

**Keywords:** Spine, Manipulation, Mobilisation, Infant, Child, Adolescent

## Abstract

**Purpose:**

To i) identify and map the available evidence regarding effectiveness and harms of spinal manipulation and mobilisation for infants, children and adolescents with a broad range of conditions; ii) identify and synthesise policies, regulations, position statements and practice guidelines informing their clinical use.

**Design:**

Systematic scoping review, utilising four electronic databases (PubMed, Embase, CINHAL and Cochrane) and grey literature from root to 4^th^ February 2021.

**Participants:**

Infants, children and adolescents (birth to < 18 years) with any childhood disorder/condition.

**Intervention:**

Spinal manipulation and mobilisation

**Outcome measures:**

Outcomes relating to common childhood conditions were explored.

**Method:**

Two reviewers (A.P., L.L.) independently screened and selected studies, extracted key findings and assessed methodological quality of included papers using Joanna Briggs Institute Checklist for Systematic Reviews and Research Synthesis, Joanna Briggs Institute Critical Appraisal Checklist for Text and Opinion Papers, Mixed Methods Appraisal Tool and International Centre for Allied Health Evidence Guideline Quality Checklist. A descriptive synthesis of reported findings was undertaken using a levels of evidence approach.

**Results:**

Eighty-seven articles were included. Methodological quality of articles varied. Spinal *manipulation* and *mobilisation* are being utilised clinically by a variety of health professionals to manage paediatric populations with adolescent idiopathic scoliosis (AIS), asthma, attention deficit hyperactivity disorder (ADHD), autism spectrum disorder (ASD), back/neck pain, breastfeeding difficulties, cerebral palsy (CP), dysfunctional voiding, excessive crying, headaches, infantile colic, kinetic imbalances due to suboccipital strain (KISS), nocturnal enuresis, otitis media, torticollis and plagiocephaly. The descriptive synthesis revealed: no evidence to explicitly support the effectiveness of spinal *manipulation* or *mobilisation* for any condition in paediatric populations. Mild transient symptoms were commonly described in randomised controlled trials and on occasion, moderate-to-severe adverse events were reported in systematic reviews of randomised controlled trials and other lower quality studies. There was strong to very strong evidence for ‘no significant effect’ of spinal *manipulation* for managing asthma (pulmonary function), headache and nocturnal enuresis, and inconclusive or insufficient evidence for all other conditions explored. There is insufficient evidence to draw conclusions regarding spinal *mobilisation* to treat paediatric populations with any condition.

**Conclusion:**

Whilst some individual high-quality studies demonstrate positive results for some conditions, our descriptive synthesis of the collective findings does not provide support for spinal *manipulation or mobilisation* in paediatric populations for any condition. Increased reporting of adverse events is required to determine true risks. Randomised controlled trials examining effectiveness of spinal manipulation and mobilisation in paediatric populations are warranted.

**Supplementary Information:**

The online version contains supplementary material available at 10.1186/s12887-022-03781-6.

## Background

Various healthcare professionals utilise manual therapy including spinal manipulation and or mobilisation as a treatment modality for musculoskeletal and non-musculoskeletal conditions. These treatment modalities are being utilised to treat paediatric clients, including infants, young children and adolescents with a variety of acute and chronic conditions [1, 2]. Manual therapy is an umbrella term that encompasses any hand movement that produces a physiological or mechanical change in soft tissue and joints [3]. Spinal mobilisation is one form of manual therapy which may be used after a thorough and extensive clinical reasoning process. It comprises a continuum of skilled passive movements applied to the spine at varying speeds and amplitudes, impacting joints, muscles or nerves with the intent to restore optimal motion and function, and to reduce pain [3]. Spinal manipulation is another form of manual therapy and is defined in Australian Health Practitioner Regulation National Law as “any technique delivered by any health professional that involves a high velocity, low amplitude (HVLA) thrust *beyond* the usual physiological range of motion, impacting the spine, within the limits of anatomical integrity” [4]. The International Chiropractic Association (ICA) utilises two terms that fit within this definition; i) ‘Spinal Adjustment’—a specific directional thrust that is believed to set the vertebra into motion with the intent to improve or correct vertebral subluxation or malposition, reducing or correcting neuroforaminal / neural canal encroachment and; ii) ‘Spinal Manipulation’ – a specific thrust to a spinal joint to mobilise the joint or put it through its range of motion [5]. Whereas, the International Federation of Orthopaedic Manipulative Physical Therapists (IFOMPT), refer to spinal manipulation as a passive, HVLA thrust applied to a spinal joint complex *within* its anatomical limit, with the intent to restore optimal motion, function, and/or to reduce pain [6].

According to the World Health Organisation (WHO), regulations guiding the utilisation of spinal manual therapy and manipulation are consistent between countries [7]. For example, in Australia, under the Health Practitioner Regulation Law (ACT) Sect. 123, a person must not perform spinal manipulation unless they are registered practitioners in one of the following health professions: Chiropractic, Osteopathy, Medical or Physiotherapy [4]. This is consistent across several other countries including but not limited to the United States of America [8] and Canada [9]. Whilst not common in the physiotherapy profession [10] or used by some evidence-based chiropractors or osteopaths [11–13], the treatment of non-musculoskeletal conditions with spinal manipulative therapy is a long-standing tradition in chiropractic and osteopathic professions [14, 15] and this is based on the underpinning theory that spinal dysfunction, or subluxations can negatively impact the autonomic nervous system and the bodies self-healing ability [16–18], and spinal manipulation can remedy this by impacting the autonomic nervous system and improving physiological functions [19, 20].

There is great controversy regarding the safety and efficacy of spinal manipulation in paediatric populations [2]. An independent expert review was commissioned by Safer Care Victoria in October 2019 and aimed to identify evidence to support position statements for both safety and efficacy of spinal manipulation in children under 12 years of age and resulted in recommendations to the Council of Australian Governments [2, 21]. An announcement by health ministers in Australia regarding spinal manipulation ensued and prompted the Chiropractic Board of Australia to enforce an interim policy prohibiting the use of chiropractic spinal manipulation in children under the age of two years [22]. When exploring the appropriateness of utilising clinical interventions, it is important to explore both effectiveness and adverse events. An adverse event is any unfavourable sign, symptom or disease associated with treatment, despite whether it was caused by the treatment [23]. Patient harm creates both a burden to patients and their families, and strains health system finances significantly. This leads to increased levels of care and resource utilisation [24].

Whilst several reviews of varying methodological quality have explored the effects and adverse events from spinal manipulation in paediatric populations [1, 21, 25–27], there have been conflicting findings published addressing a broad spectrum of conditions and there has been little exploration of the policies, guidelines, regulations or laws, supporting or prohibiting the use of spinal manipulation or mobilisation in the management of infants, children and adolescents. Some reviews on this topic have limited their inclusions to explore the effects or harms in infants, and there has been less exploration of the effects or harms of spinal manipulation and mobilisation of children aged 12 years or older. The conflicting information in published reviews conducted to date, appears to be due, at least partially, to the inclusion of low-quality research or lack of critical appraisal for included studies [28–30]. There has been limited publication of policies, guidelines and position statements regarding the use of spinal manipulation and mobilisation of paediatric clients, with only one review exploring this in paediatric populations from birth – 12 years [21]. Both the inconsistency of empirical research findings and the apparent lack of guidance documents to support or restrain practice in this clinical area, leaves both healthcare professionals and paediatric clients vulnerable to inappropriate, ineffective, or potentially harmful interventions and a broader synthesis of the collective literature to guide clinicians in this clinical area is warranted.

The purpose of this systematic scoping review was to identify and map the available evidence related to the use of spinal manipulation and mobilisation techniques in the treatment of infants, children and adolescents with a variety of common paediatric conditions. This systematic scoping review was planned as a joint investigation by the International Federation of Orthopaedic Manipulative Physical Therapists [IFOMPT] and International Organisation of Physical Therapists in Paediatrics [IOPTP] to inform future position statements on this clinical practice topic and guide more focused research investigations if warranted. In this systematic scoping review, we identified and mapped the results of empirical research, reviews of empirical research, published guidelines for practice, policies and position statements. In relation to infants, children and adolescents, we addressed the following questions:

1. What conditions are being managed with spinal manipulation and mobilisation?
2. Is spinal manipulation and mobilisation effective?
3. Is spinal manipulation and mobilisation harmful?
4. Are there policies, regulations, position statements and practice guidelines informing the clinical use of spinal manipulation and mobilisation?

## Methods

The PRISMA statement extension for scoping reviews (PRISMA-ScR) was used to guide the reporting of this systematic scoping review [31]. The review protocol was registered with Open Science Framework on June 14, 2020 (Retrieved from https://osf.io/zm8e6) prior to conducting the search.

### Identification and selection of studies

After consulting with the Health Sciences and Medicine Faculty librarian at the host university, the appropriate Medical Subject Headings (MeSH terms) and Boolean operations were incorporated before the empirical literature was systematically searched, combining synonyms for “infant”, “child” and “adolescent”, and key words related to “spinal manipulation” and “spinal mobilisation”, followed by outcomes associated with common childhood conditions. The following databases were searched: PubMed, Embase, CINAHL and Cochrane. Grey literature was searched using Google utilising key terms including “paediatric” (and associated synonyms) AND “spinal manipulation” OR “spinal mobilisation” (and associated synonyms) AND “policies” OR “guidelines” OR “statements”, hand-searching reference lists from all included research articles and reviewing articles via expert referral of relevant literature. The search strategy was wide in scope to support the nature of the review and details on the search strategy are presented in Supplementary File 1.

The four databases were searched from root up to 18 June 2020 with an updated search up to 4^th^ February 2021. To identify relevant grey literature, a google search for files ending with [file: PDF] and [file: doc] was conducted. The initial and follow-up search was performed independently by two authors (A.P. and L.L.). Studies were gathered, and duplicates were removed using EndNote (Endnote Version X9.1.1, Clarivate Analytics; 2019). Once duplicate articles were removed, two authors (A.P. and L.L.) independently conducted title and abstract screening to identify potentially relevant articles for full-text review. After undertaking an initial process of consensus, outstanding disagreements between two authors (A.P. and L.L.) were resolved by a third author (N.M.). Studies that appeared to meet the inclusion criteria at title and abstract screening stage were retrieved in full text. Eligibility criteria were applied. Table 1 provides a comprehensive list of the inclusion criteria for both research articles and grey literature. Studies were excluded if individuals were aged over 18 years, if manual therapy techniques were applied to areas of the body other than the spine, if paediatric data was unable to be extracted from mixed populations or if it was an animal study. Grey literature was searched to gain a deeper understanding of current professional services regarding the use of spinal manipulation and or mobilisation. We excluded documents that did not have an attributed author or publisher and protocols with no full published study were excluded. To achieve a final consensus on included full text articles, all discrepancies were resolved by a third reviewer (N.M.). Reference lists of included articles were hand-searched for other possible articles that may have been missed during the initial search. The results of the search are presented in flowchart format according to the PRISMA extension for scoping reviews (PRISMA-ScR) [31].

**Table 1:** Inclusion Criteria

Design
• Full-text articles published in English language only
• Research articles: systematic reviews, randomised controlled trials (RCTs), intervention studies, observational studies, cross sectional studies
• Grey literature: policies, procedures, guidelines, recommendations, position statements or perspectives (including commentaries, opinions and editorials)
**Participants**
• Study participants must be male or female infants (0 to < 2), children (2 to 12) or adolescents (13 to < 18) (WHO, 2006)
**Intervention**
• Study participants must have had spinal manipulation and/or spinal mobilisation carried out by health professionals with an international body guiding their practice
**Outcome**
• Patient/caregiver reported outcome (PRO), observation-based outcomes, other structure impairment, reports, policy statement or recommendation statement related to body function or structure impairment, activity limitation or participation restriction
• Adverse events and harms
**Comparison (for intervention studies)**
• Any comparison group in a randomised or non-randomised study: placebo, waitlist, no treatment, adjunct treatment, or comparison intervention

## Assessment of characteristics of reviews and studies

### Quality appraisal

The quality assessment process was independently conducted by two authors (A.P. and L.L.) (see Supplementary File 2) and a summary of the critical appraisal scores has been summarised. Cohen’s kappa statistic was applied to determine the level of agreement in scoring between the two reviewers and disagreements were settled by a third reviewer (N.M.). The following critical appraisal tools were used to assess quality due to diversity of included study designs, while grey literature which did not fulfil the critical appraisal tools below were not critically appraised.

1. The Joanna Briggs Institute (JBI) Checklist for Systematic Reviews and Research Synthesis was used to assess quality of included Systematic Reviews [32] (Supplementary File 2). This tool includes 11 domains and criteria were assessed using the following scoring: ‘yes’ scoring ‘1’ and ‘no’ or ‘unclear’ scoring ‘0’.
2. The Mixed Methods Appraisal Tool (MMAT) was used to assess the quality of the quantitative and qualitative studies [33] (Supplementary File 2). MMAT appraises quality of five categories including qualitative research, randomised control trials (RCT), non-randomised studies, quantitative descriptive studies and mixed methods studies. Criteria were assessed by scoring ‘yes’ as ‘1’ and ‘no’ or ‘can’t tell’ as ‘0’.
3. The International Centre for Allied Health Evidence Guideline Quality Checklist (ICAHE) was used to assess the quality of guidelines included in grey literature [34] (Supplementary File 2). This tool includes six domains: availability, dates, underlying evidence, guideline developers, guideline purpose and users, and ease of use. Criteria were assessed by scoring ‘yes’ as ‘1’ and ‘no’ as ‘0’.
4. The Joanna Briggs Institute (JBI) Critical Appraisal Checklist for Text and Opinion Papers was used to assess quality of the text and opinion papers [35] (Supplementary File 2). This tool includes six domains: source, expertise, relevant population, logic, reference to the literature and incongruence with the literature. Criteria were assessed by scoring ‘yes’ as ‘1’ and ‘no’ or ‘unclear’ as ‘0’.

### Data extraction and analysis

Data was extracted independently by two authors (A.P. and L.L.) using a standardised pre-piloted data extraction form (see Supplementary File 3) to collect relevant information including study design, participant characteristics, intervention and outcome measures. A third author (N.M.) ensured accuracy and validity of extracted data. Information relating to adverse events and harms were extracted from systematic reviews and individual studies when reported. Adverse events were then classified using a modified version of the common terminology criteria for adverse events (CTCAE) published in the Adverse Event Reporting Requirements by the National Cancer Institute [36] and were considered ‘mild’ – if individuals were experiencing mild symptoms requiring self-care only; ‘moderate’—if symptoms were limiting age-appropriate activities of daily living or requiring care from a physician and; ‘severe’ – if experiencing medically significant symptoms leading to a life-threatening outcome resulting in urgent intervention, hospitalisation or death [36]. Authors of papers were contacted to request missing or additional data if required.

All included articles were reviewed to identify the presenting paediatric conditions being treated with spinal manipulation and/or mobilisation. The form of intervention used in the studies was identified and classified as “spinal manipulation”, “spinal mobilisation” or a combination of both alongside other treatment modalities (e.g., “soft tissue massage” or “exercise”). For transparency of overlap between studies and reviews, a matrix (Supplementary File 4) was developed to identify the percentage of overlap for included studies which were already represented in the included review articles. Only studies that achieved 5/7 or more on critical appraisal (i.e., higher quality studies), would also be included in the descriptive synthesis using a levels of evidence approach. Information from each systematic review was extracted and represented according to the focus of the paediatric conditions and impairments.

After data extraction, a descriptive synthesis was completed to explore the effectiveness of spinal manipulation and mobilisation with paediatric populations. The descriptive synthesis involved two stages. Initially, the results from investigations (reviews and studies) were coded based on whether the effect was significantly positive (i.e., favourable) ( +), negative (i.e., unfavourable) ( −), had no significant effect (0) or was inconclusive (Inc – for reviews only). For individual studies with control groups when there was no difference in effect between control (standard care) and intervention groups (standard care, plus spinal manipulation or mobilisation), a code of zero (0) was applied, or a statistically significant difference (*p* < 0.05) favouring the intervention group or control group was coded (+ or -) respectively [37]. Results from systematic reviews were only included in the descriptive synthesis when more than one study was synthesised in the review and was relevant to the outcome explored in that review. If only one study was included in a review, that study was identified in the individual studies, inclusion and exclusion criteria were applied, as were critical appraisal methods. To be included in the descriptive synthesis, studies had to be of good methodological quality scoring at least five out of seven on the MMAT tool. Reviews that did not synthesise data were excluded from the descriptive synthesis stage of analysis.

**Fig. 1:**
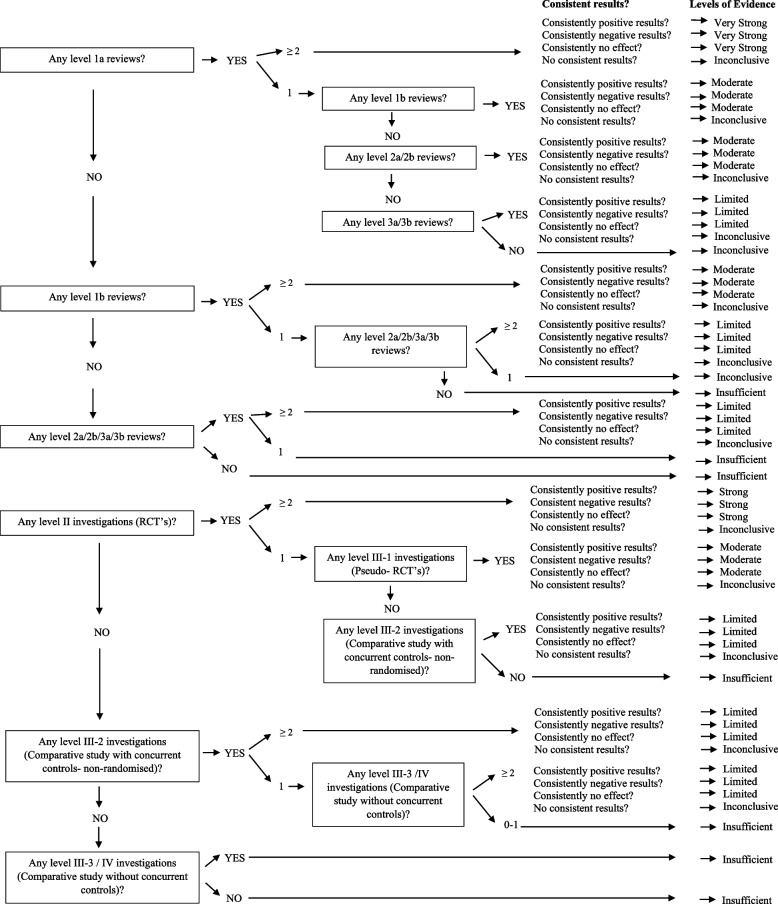
Flow chart of decision-making process for levels of evidence approach, based on study design, quality and quantity

Finally, to ensure that findings reported were from the highest available level of evidence, a levels of evidence approach adapted from previously published literature [37–40] was utilised to assess both the quality and quantity of evidence (reviews and studies) relating to the outcomes for defined impairments for each condition (Fig. 1). After following the decision tree in Fig. 1 which is based on the quality of evidence and quantity of such evidence, the levels of evidence statements available for each outcome were: Very strong, strong, moderate or limited evidence for a positive (favourable) effect, negative (unfavourable) effect or no significant effect. Consistent positive results (≥ 66.6% of relevant investigations at the identified level reporting significant positive results) or consistent negative (66.6% of relevant investigations at the identified level reporting significant negative results) were needed to achieve very strong, strong, moderate or limited levels of evidence statements. Consistently no significant effect (≥ 66.6% of relevant investigations at the identified level reporting no significant effect) was required to determine that the intervention has ‘no significant effect’ on the condition/outcome. If the above percentages were not reached and the results of the decision tree were mixed, the evidence for that intervention was deemed to be ‘inconclusive’ and if there were insufficient studies / reviews exploring the intervention for the identified condition / outcome, then ‘insufficient’ evidence was documented for the levels of evidence statement. If evidence from the systematic reviews resulted in statements of ‘insufficient’ or ‘inconclusive’, collective results from individual studies (if available) were utilised for the final level of evidence statement. All levels of evidence are based on previously published National Health and Medical Research Council (NHMRC) levels of evidence hierarchy for studies [40] and JBI levels of evidence for systematic reviews [39]. The level of evidence utilised are summarised in Table 2 below.

**Table 2:** Levels of Evidence Definitions used for Descriptive Synthesis

Level of Evidence	Study Types
Reviews (JBI, 2013)	
Level 1	Level 1.a – Systematic review of Randomised Controlled Trials (RCTs) Level 1.b – Systematic review of RCTs and other study designs
Level 2	Level 2.a – Systematic review of quasi-experimental studies Level 2.b – Systematic review of quasi-experimental and other lower study designs
Level 3	Level 3.a – Systematic review of comparable cohort studies Level 3.b – Systematic review of comparable cohort and other lower study designs
Level 4	Level 4.a – Systematic review of descriptive studies
Studies (NHMRC, 2009)	
II	A randomised controlled trial
III-1	A pseudorandomised controlled trial (i.e., alternate allocation or some other method)
III-2	A comparative study with concurrent controls: ▪ Non-randomised, experimental trial ▪ Cohort study ▪ Case–control study ▪ Interrupted time series with a control group
III-3	A comparative study without concurrent controls: ▪ Historical control study ▪ Two or more single arm study ▪ Interrupted time series without a parallel control group
IV	Case series with either post-test or pre-test/post-test outcomes

*RCT* Randomised Controlled Trials, *NHMRC* National Health and Medical Research Council, *JBI* Johanna Briggs Institute

Source: [39, 40]

## Results

### Flow of studies through the scoping review

**Fig. 2:**
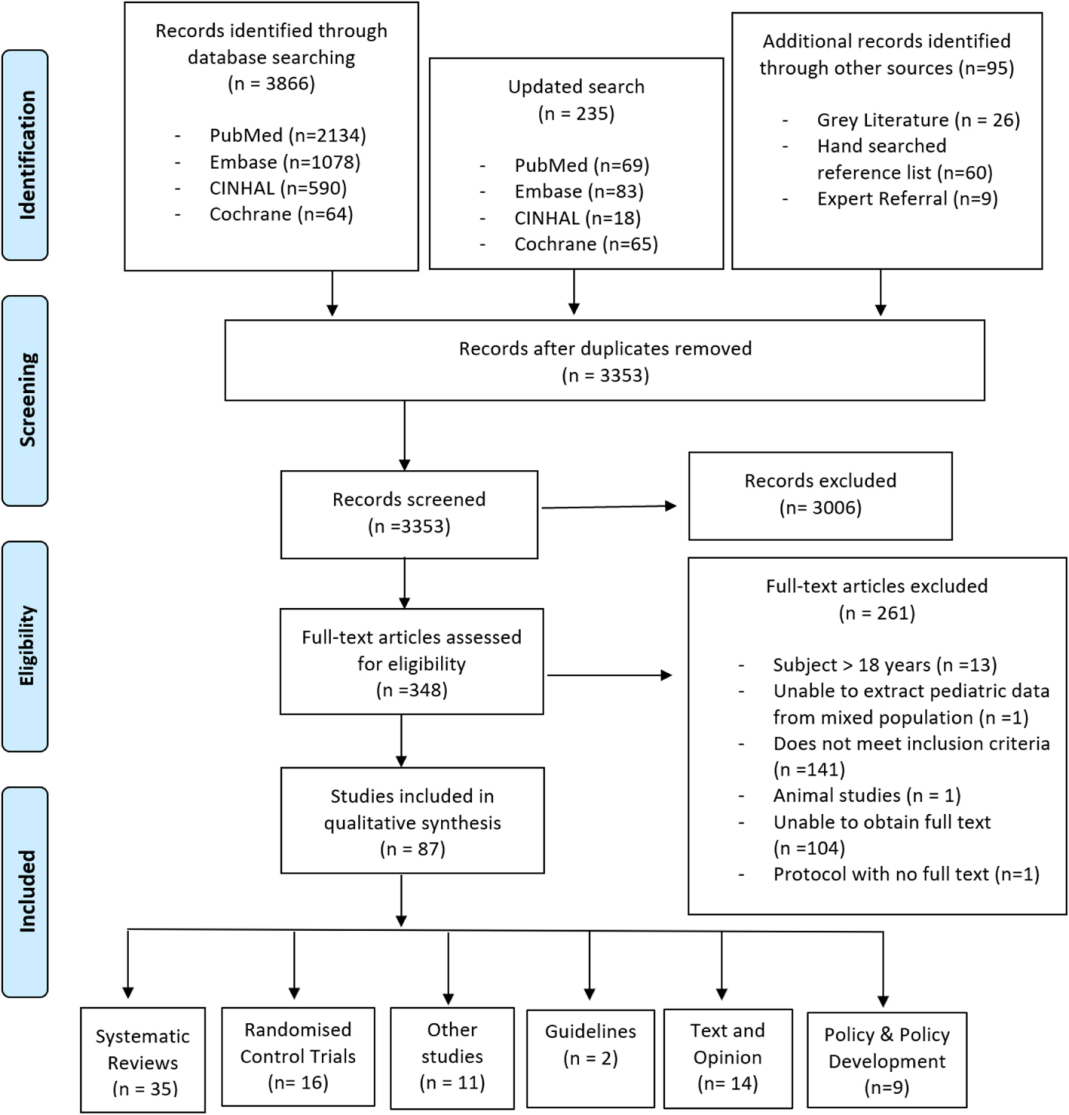
PRISMA flow diagram [31]

The initial literature search yielded 3866 papers (Fig. 2) with 95 additional studies included from scanning reference lists or other sources, and after applying the inclusion and exclusion criteria in the screening process, 348 full text articles were assessed for eligibility and 87 articles met the eligibility criteria (Table 1).

Of the 87 included articles, 35 were systematic reviews with seven being level 1a reviews according to the JBI Levels of Evidence for Systematic Reviews [1, 41–46], 16 RCT’s, 11 other studies (*n* = 2 surveys, *n* = 1 naturalistic study, *n* = 5 cohort studies, *n* = 1 prospective outcome study, *n* = 1 retrospective study, *n* = 1 feasibility study), two guidelines, 14 text and opinion papers and nine policy and policy developments (Fig. 2).

The matrix presented in Supplementary File 4 revealed that only 1 systematic review [21] captured a large proportion of the studies from the present scoping review. The descriptive synthesis in the present scoping review was undertaken for reviews, and studies (scoring ≥ 5/7 on the MMAT) independently and any differences are discussed below.

### Characteristics of the included studies

#### Quality

A summary of critical appraisal consensus scores for all studies and grey literature is reported in Table 3 with a detailed breakdown of individual critical appraisal scores in Supplementary File 2. There was moderate inter-rater agreement on the critical appraisal score between the two independent reviewers (κ = 0.61, *p* =  < 0.001). After a process of consensus, 100% agreement was achieved for all papers during the consensus process. Critical appraisal revealed that review articles generally scored poorly in questions regarding methods to minimise errors in data extraction, and in their assessment of the likelihood of publication bias. Regarding grey literature (see Supplementary File 2), one guideline lacked underlying quality of evidence [47]. Critical appraisal revealed the methodological quality of text and opinion papers was mostly reduced due to poor reporting of the source of opinion, not using an analytical process to form an opinion, or logically defending incongruences in the literature (see Supplementary File 2).

**Table 3:** Summary of Critical Appraisal Scores (CAS)

JBI for Systematic Reviews		**MMAT- RCT’s**		**MMAT- Other studies**		**Grey Literature**	
**Author (Year)**	**CAS**	**Author (Year)**	**CAS**	**Author (Year)**	**CAS**	**Author (Year)**	**CAS**
Alcantara, et al. (2011a) [48]	3/11	Balon (1998) [49]	7/7	Alcantara, et al. (2009) [50]	6/7	**iCAHE – Clinical Guidelines**	
Alcantara, et al. (2011b) [51]	8/11	Borusiak (2009) [52]	5/7	Davies & Jamison (2007) [53]	6/7	Council of Chiropractic Practice (2008) [54]	13/14
Alcantara, et al. (2015) [55]	4/11	Bronfort et al. (2001) [56]	5/7	Hayden, et al. (2003) [57]	6/7	NSW Government (2016) [47]	9/14
Brand, et al. (2005) [41]	7/11	Browning and Miller (2008) [58]	7/7	Lantz and Chen (2001) [59]	5/7		
Bronfort, et al. (2010) [1]	7/11	Cabrera-Martos, et al. (2016) [60]	7/7	Lebouef (1991) [61]	3/7	**JBI for Text and Opinion Papers**	
Brurberg, et al. (2019) [62]	6/11	Dissing, et al. (2018) [63]	6/7	Miller and Benfield (2008) [64]	7/7	World Federation of Chiropractic (WFC) (2019) [65]	2/6
Carnes, et al. (2018) [66]	11/11	Evans, et al. (2018) [67]	6/7	Miller and Phillips (2009) [68]	5/7	Chiropractic Board of Australia (CBA) (2017) [69]	4/6
Clar, et al. (2014) [25]	10/11	Haugen, et al. (2011) [70]	5/7	Miller and Newell (2012) [71]	5/7	Chiropractors’ Association of Australia (CAA) (2016) [72]	4/6
Corso, et al. (2020) [73]	11/11	Kachmar, et al. (2018) [74]	7/7	Saedt, et al. (2018) [75]	5/7	International Chiropractic Association (ICA) (2019) [76]	6/6
Dobson, et al. (2012) [42]	10/11	Lynge, et al. (2021) [77]	6/7	Sawyer, et al. (1999) [78]	6/7	Barham-Floreani (2014) [79]	4/6
Driehuis, et al. (2019) [26]	10/11	Miller, Newell & Bolton. (2012) [80]	5/7	Zhang and Snyder (2004) [81]	5/7	Marron (2011) [82]	6/6
Edwards and Miller (2019) [28]	8/11	Nemett (2008) [83]	4/7			Chevrier (2016) [84]	6/6
Ellwood, et al. (2020) [27]	10/11	Olafsdottir, et al. (2001) [85]	7/7			Kirkey (May 2019) In College of Chiropractors of Ontario (2019) [86]	3/6
Ernst (2009) [43]	6/11	Reed (1994) [87]	2/7			Kirkey (July 2019) In College of Chiropractors of Ontario (2019) [88]	2/6
Fairest, et al. (2019) [29]	3/11	Selhorst and Selhorst (2015) [89]	7/7			Collie (2019) In College of Chiropractors of Ontario (2019) [90]	2/6
Ferrance and Miller (2010) [91]	3/11	Wiberg, et al. (1999) [92]	5/7			Lindsay (2019) In College of Chiropractors of Ontario (2019) [93]	3/6
Fry (2014) [30]	5/11					Rosner (2003) [94]	5/6
Glazener, et al. (2005) [44]	10/11					Australian Chiropractic Association (ACA) (2019) [95]	4/6
Gleberzon, et al. (2012) [96]	10/11					Sellhorst (2015) [97]	5/6
Green, et al. (2019) [21]	10/11						
Hawk, et al. (2007) [98]	8/11						
Hawk, et al. (2019) [99]	11/11						
Hondras, et al. (2005) [45]	10/11						
Huang, et al. (2011) [46]	7/11						
Humphreys (2010) [100]	5/11						
Karpouzis, et al. (2010) [101]	7/11						
Kronau, et al. (2016) [102]	11/11						
Lucassen (2010) [103]	7/11						
Parnell, et al. (2019) [104]	11/11						
Pohlman & Holton-Brown (2012) [105]	8/11						
Romano & Negrini (2008) [106]	6/11						
Theroux, et al. (2017) [107]	10/11						
Todd, et al. (2015) [108]	7/11						
Vaughn, et al. (2012) [109]	11/11						
Vohra et al. (2007) [110]	7/11						

Key: *JBI* Joanna Briggs Institute for Systematic Reviews (0 to 11), *MMAT* Mixed Methods Appraisal Tool (0 to 7), *iCAHE* International Centre for Allied Health Evidence Guideline Quality Checklist (0–14), *JBI* Joanna Briggs Institute for Text and Opinion Papers (0–6), *CAS* Critical Appraisal Scores

#### Participants

Participants represented in both the systematic reviews and studies ranged from birth to ≤ 18 years (Supplementary File 3). The included articles assessed the effects of spinal manipulation or mobilisation to manage a variety of impairments related to many different conditions, including: adolescent idiopathic scoliosis (AIS), asthma and breathing difficulties, autism spectrum disorder (ASD), attention deficit-hyperactivity disorder (ADHD), back/neck pain, breastfeeding difficulties, cerebral palsy (CP), dysfunctional voiding, headache, infantile colic (excessive crying and sleep disturbances), kinetic imbalance due to suboccipital strain (KISS) disorder, nocturnal enuresis, otitis media, torticollis and plagiocephaly. Supplementary File 3 presents a detailed description of all included articles with relevant data extracted. Table 4 outlines the number of included articles exploring spinal manipulation and mobilisation according to study design and age groups explored.

**Table 4:** Participant type in included articles

Study Design	I	C	A	I + C	C + A	I + C + A	Unspecified Paediatric
Reviews on spinal manipulation	10	1	1	3	5	10	-
Reviews on spinal mobilisation	1	-	-	-	-	-	-
RCTS and other studies on spinal manipulation	8	3	1	-	9	-	-
RCTS and other studies on spinal mobilisation	3	-	-	1	-	-	-
Reviews on spinal manipulation and mobilisation	-	1	-	1	-	1	1
RCTS and other studies on spinal manipulation and mobilisation	-	-	-	1	-	1	-

Key: *I* Infants, *C* Children, *A* Adolescents. NB: Duplication exists between reviews and studies

### Intervention

Interventions explored in the present systematic scoping review included spinal manipulation and mobilisation. These interventions were conducted by health professionals with guiding international professional bodies [65, 111–113] including chiropractors (18 reviews, 8 RCTs, 10 other studies), physiotherapists (4 RCTs) a combination of chiropractors, osteopaths, physiotherapists and/or manual therapists (17 reviews and 2 RCTs), medical doctors specialising in manual therapy (2 RCTs) and a manual therapist (not otherwise defined) (1 RCT and 1 other study).

Below is a summary of findings from the included reviews and studies, including the results from the descriptive synthesis in Supplementary File 5 regarding the effectiveness of spinal manipulation and mobilisation. The effects of spinal *manipulation* and *mobilisation* have been reported separately according to the conditions being managed in paediatric populations (see Supplementary File 5).

#### Effects of spinal manipulation in infants, children and adolescents

Of the 35 included reviews, 24 investigated the effectiveness of spinal *manipulation* in paediatric clients and produced quantifiable results which could be utilised in the descriptive synthesis (Supplementary File 5). Three were focused on treatment for AIS [104, 106, 107], seven on asthma [1, 21, 45, 91, 96, 98, 104], two for ASD [51, 102], two on spinal pain [104, 109], four on breastfeeding difficulties for infants [28, 30, 99, 104], two on CP [25, 104], 15 on infantile colic—excessive crying / behaviours [1, 21, 25, 26, 42, 43, 66, 91, 96, 98, 103, 104], four on infantile colic – sleep issues [42, 66, 91, 104], five on nocturnal enuresis [26, 44, 46, 96, 98], three on otitis media [98, 104, 105] and one on torticollis [62] (see Supplementary File 5). Additionally, there were four systematic reviews on adverse events from spinal manipulation [73, 100, 108, 110] and nine reported on multiple conditions including those mentioned above as well as neck and back pain, and upper cervical dysfunction [1, 21, 25, 26, 91, 96, 98, 104, 109].

From the 18 studies included in the descriptive synthesis that explored the effectiveness of spinal manipulation in paediatric populations, one was focused on AIS [59], two on asthma [49, 56], four on back/neck pain [57, 63, 67, 89], one on CP [74], two on headache [52, 77], six on infantile colic – excessive crying / behaviours [53, 58, 68, 71, 85, 92], one on infantile colic – sleeping disturbances [58] and one on torticollis [70].

Only 14 of the 62 included research articles provided supporting evidence (e.g., references to other papers) of psychometric properties for the outcome measures being utilised in the research evaluations and none of the articles provided actual psychometric values for reliability, validity and responsiveness, to suggest the selected outcome measure was a suitable tool to measure effectiveness of treatment. The findings from the descriptive synthesis using the levels of evidence approach are provided below for each condition which met our methodological thresholds for undertaking a descriptive synthesis.

##### Adolescent idiopathic scoliosis (AIS)

From three reviews [104, 106, 107] and one observational study [59] exploring spinal manipulative therapy for treating scoliosis, our ***descriptive synthesis**** revealed ‘inconclusive’ results for using spinal manipulation to manage impairments and symptoms of AIS* (Table 5)*.*

**Table 5:** Summary results of descriptive synthesis with levels of evidence statement for spinal MANIPULATION to manage paediatric populations with a variety of conditions

**Conditions *(Population)***	**Levels of Evidence Statement**			**Adverse Events** Documented in reviews and studies included in the descriptive synthesis		
	Reviews	High-Quality Studies	Summary Statement			
				Original Report (Author & Year)	Adverse Event / (Practitioner Type)	Further cited by
**Spinal MANIPULATION**						
Adolescent Idiopathic Scoliosis ^ (AIS) *(C&A)*	Inconclusive	Insufficient	Inconclusive	Rowe (2006)	Two benign reactions (no further detail documented) (Chiropractic)	Theroux (2017) [107] Todd (2015) [108]
Asthma *(C&A)*	Inconclusive	STRONG Evidence of No Significant Effect	STRONG Evidence of No Significant Effect ^#^			
ASD ^ *(C&A)*	Inconclusive	-	Inconclusive			
Spinal (Back / Neck) Pain ^ *(C&A)* *(Combined Chronic and Acute Pain)*	Inconclusive	Inconclusive	Inconclusive	*Evans (2018)	Unusual or increased soreness (51%-54%) and different type of pain (31%-34%) (Chiropractic)	
				L’Ecuyer (1959)	Neck pain in 12-year-old girl with a history of congenital torticollis, progression to unsteady gait, poor coordination, drowsiness, and hospitalisation with delayed diagnosis of congenital occipitalisation. (Chiropractic)	Green (2020) [21] Vohra (2007) [110]
				Ziv (1983)	Back pain in 12-year-old girl with history of osteogenesis imperfecta—progressive neuromuscular deficits in legs, clonus at rest, urinary urgency and frequency, paraplegia. (Chiropractic)	Vohra (2007) [110]
Breastfeeding Difficulties ^ *(I)*	Inconclusive	-	Inconclusive			
CP ^ *(I, C, A)*	Inconclusive	Insufficient	Inconclusive			
Headache *(C&A)*	Insufficient	STRONG Evidence of No Significant Effect	STRONG Evidence of No Significant Effect	Borusiak (2010)	Hot skin and dizziness, transitory increase in headache intensity and frequency and loss of consciousness in both treatment sessions; quick recovery once treatment stopped. (Manual therapist)	Green (2020) [21]
				Zimmerman (1978)	Severe occipital and bifrontal headache, vomiting, left facial weakness, diplopia ataxia. (Chiropractic)	Vohra (2007) [110]
				Held (1966)	Acute respiratory decompensation, tracheotomy, neurologic deficits at C6 and C7 vertebrae, neck pain. (Medical practitioner)	Vohra (2007) [110]
Infantile Colic – crying *(I)*	Inconclusive	Inconclusive	Inconclusive	Miller (2012)	One child in comparison group reported an AE of increased crying. Incidence of increased crying in comparison group:2.94% (0.52, 14.92) (Chiropractic)	Corso (2020) [73]
				Shafrir (1992)	In three of these studies a small number of mild harms was reported; the other two studies (*n* = 145) reported no harms. One study (*n* = 956) reported side effects or reactions in children after chiropractic treatment (*n* = 557), but both side effects or reactions and treatment techniques were not specified. (Chiropractic)	Driehuis (2019) [26] Ellwood (2020) [114]
Infantile Colic^ – Sleeping *(I)*	Inconclusive	Insufficient	Inconclusive	Koch (1998)	Vegetative reactions, bradycardia, tachycardia, and reflex apnoea recorded in more than half of patients. Although apnoea was of short duration (< 10 s) and reversible, it can be regarded as a potentially life-threatening adverse event. NB: Conditions were mixed (colic, opistotonus, hypertonus, wryneck, plagiocephaly, scoliosis, limb weakness and slobbering). (Chiropractic)	Brand (2005) [41]
				Wilson (2012)	Severe: Rib fractures (7^th^ and 8^th^ posterior) (Chiropractic) NB: referral reason not confirmed	Todd (2015) [108]
Nocturnal Enuresis *(C&A)*	VERY STRONG Evidence of No Significant Effect	-	VERY STRONG Evidence of No Significant Effect	Leboeuf (1991)	4 – 15-year-old children and adolescents: Severe headache, stiff neck and acute lumbar spine pain. (5^th^ year Chiropractic students)	Green (2020) [21] Vohra (2007) [110] Hawk (2007) [98] Glazener (2005) [44]
Otitis Media ^ *(I & C)*	Inconclusive	Insufficient	Inconclusive	Sawyer (1999)	6-months to 6 years—Mid-back soreness and increased irritability (Academic Chiropractors)	Green (2020) [21] Corso (2020) [73] Vohra (2007) [110] Hawk (2007) [98] Pohlman (2012) [105] Glazener (2005) [44]
Torticollis ^ *(I & C)*	Insufficient	Insufficient	Insufficient	Jacobi (2001)	Subarachnoid haemorrhage and death of 3-month-old girl (presenting condition unclear) (Physiotherapist)	Green (2020) [21] Vohra (2007) [110] Ellwood (2020) [27] Driehuis (2019) [26] Brand (2005) [41]
				Shafrir (1992)	Quadriplegia in 4-month-old boy, secondary to spinal cord astrocytoma; regressed to paraplegia (18 months postoperatively). (Chiropractic)	Todd (2015) [108] Driehuis (2019) [26] Ellwood (2020) [27]

All findings presented in this table are a result of the descriptive synthesis presented in Supplementary File 5

High quality evidence was not available to explore the effectiveness of spinal manipulation on individuals with the following conditions: Attention Deficit Hyperactivity Disorder (ADHD), dysfunctional voiding, KISS syndrome, upper cervical dysfunction

Populations: I – Infants, C – Children, A – Adolescents

^#^Asthma—spinal manipulation on paediatric populations had ‘no significant effect’ on pulmonary function and findings were inconclusive for peak expiratory flow, general asthma symptoms, severity levels and quality of life

^^^Additional high-quality research (e.g., RCTs) may be warranted

All adverse events extracted from included systematic reviews, except those studies marked with * which have been extracted from individual studies

Insufficient: Insufficient high-quality evidence available on the topic and further research may be warranted

Inconclusive: Available evidence is inconclusive, and further research may be warranted

No Significant Effect: High-quality evidence suggests the intervention is not effective and should not be used in clinical practice

Significant Positive Effect: High-quality evidence suggests the intervention is effective and could be used when clinical reasoning supports its application

##### Asthma

From seven reviews [1, 21, 45, 91, 96, 98, 104] and two RCTs [49, 56], our *>descriptive synthesis revealed very strong evidence that spinal manipulation on paediatric populations had ‘no significant effect’ on pulmonary function and findings were inconclusive for peak expiratory flow, general asthma symptoms, severity levels and quality of life* (Table 5).

##### Autism spectrum disorder (ASD)

Of the two reviews [102, 104], one concluded that there was a reduction in Autism related symptoms after spinal manipulation [102], however, results from this review must be interpreted with caution as many included studies were case reports. There were no individual studies of good methodological quality exploring this topic included in the present scoping review. *Our descriptive synthesis revealed ‘inconclusive’ findings for spinal manipulation to treat autism related impairments in children* (Table 5).

##### Attention deficit hyperactivity disorder (ADHD)

Whilst our scoping review captured two systematic reviews that explored the effectiveness of spinal manipulation in paediatric populations with ADHD [21, 104], both reviews included the same single study on the topic, which was screened for inclusion in our review but excluded due to not meeting our definition for spinal manipulation. No additional studies were identified on this topic and subsequently there was *not sufficient evidence to complete a descriptive synthesis on the effects of spinal manipulation for children with ADHD* (Table 5).

##### Spinal (Back / Neck) Pain (mixed acute and chronic presentations)

Two reviews [104, 109] explored the effectiveness of spinal manipulation for managing low back pain severity, with one review [104] (*n* = 1 RCT and 1 observational study exploring 239 participants) concluding favourable outcomes for reducing a mixture of acute and chronic back pain severity in children and adolescents, with the second review [109] finding inconclusive results regarding the effectiveness of spinal manipulation for managing a mixture of acute and chronic spinal pain. Additionally, four studies [57, 63, 67, 89] explored the effects of back and neck pain (with mixed acute and chronic presentations) in children and adolescents. One well powered RCT [67] showed spinal manipulation (added to exercise) had significant favourable effects on reducing chronic low back pain severity and one lower quality study [57] showed spinal manipulation resulted in significantly favourable reductions in severity of acute back pain. However, two additional RCTs [63, 89] and one other study [57] provided strong evidence that spinal manipulation had ‘no significant effect’ on spinal pain (mixed acute and chronic) severity in children and adolescents despite strong evidence of improvements in global perceived effects rated by caregivers [63, 67]. There is ‘insufficient’ research to conclude the effectiveness of spinal manipulation on recurrence of spinal (back and neck) pain, episode length, pain medication use, and quality of life in children and adolescents (Table 5). There is also ‘insufficient’ research to conclude if spinal manipulation is effective for managing paediatric populations presenting with chronic (only) spinal pain or acute (only) spinal pain. Consequently, o*ur descriptive synthesis of the collective evidence suggests the effectiveness of spinal manipulation for managing spinal (back and neck) pain in paediatric populations remains ‘inconclusive’.*

##### Breastfeeding difficulties

Of the four reviews [28, 30, 99, 104], two suggested there was favourable findings for the use of spinal manipulative therapy of infants with breastfeeding difficulties, however, one of these was a low level (Level 3b) review [99] and the other reviews [28, 104] did not support these findings. No additional studies were included on this topic. *Our descriptive synthesis suggests that the evidence for using spinal manipulation in infants to improve breastfeeding outcomes is ‘inconclusive’* (Table 5).

##### Cerebral palsy (CP)

Two reviews [25, 104] explored the use of spinal manipulation in children for managing a variety of impairments associated with CP and both determined there was inconclusive evidence for its effectiveness. Whilst a single RCT with 78 participants [74] provides evidence of significant desirable effects for spinal manipulation in children and adolescents for reducing spasticity in wrist muscles, our *descriptive synthesis suggests ‘inconclusive’ findings regarding the effectiveness of spinal manipulation to manage impairments of CP in children* (Table 5).

##### Dysfunctional voiding

Whilst two reviews [25, 104] which investigated osteopathic manipulative therapy for improving symptoms related to dysfunctional voiding in children, were captured in the present scoping review, neither met the requirements for inclusion in the descriptive synthesis as each review only included one study on the topic. Additionally, no individual studies were captured, therefore a *descriptive synthesis on this topic could not be undertaken* (Table 5).

##### Headache

A single systematic review exploring the effectiveness of spinal manipulation for improving impairments related to headache in children and adolescents [104], was included and indicated inconclusive results. Two included RCTs [52, 77] have explored the effects of spinal manipulation across six different outcomes related to headache in children and adolescents. Whilst one large (*n* = 194) RCT [77] found spinal manipulation significantly reduced the number of days with headache and significantly enhanced the global perceived effect from parents, the collective included evidence exploring the effects of spinal manipulation demonstrated no significant changes to the duration of headache, days of school missed due to headache, consumption of analgesics or intensity of headache (see Supplementary File 5). Subsequently our *descriptive synthesis of the collective research revealed there is strong evidence that spinal manipulation has ‘no significant effective’ on headache* (Table 5).

##### Infantile colic

From the twelve reviews [1, 21, 25, 26, 42, 43, 66, 91, 96, 98, 103, 104] that explored the effects of spinal manipulation for managing crying / behaviour related impairments of infantile colic, four [42, 66, 96, 98] demonstrated significant positive results in infants for reducing crying time and improved symptoms. However, all other reviews demonstrated no significant effect, negative effects, or inconclusive findings (see Supplementary File 5). Two additional RCT’s [58, 92] and two other studies [68, 71] showed significant positive effects for reducing crying time and later symptoms as a toddler, with other RCTs [85, 92] and studies [53, 71] demonstrating no significant effects from spinal manipulation in infants (see Supplementary File 5). Four reviews [42, 66, 91, 104] explored the effects of spinal manipulation for improving sleep time for infants with colic and all found inconclusive results, except Dobson [42] who found significant improvements. One additional RCT captured in our review [58] showed significant improvements in sleep time from spinal manipulation in infants with colic. Consequently, our *descriptive synthesis revealed ‘inconclusive’ findings for the effectiveness of spinal manipulation to manage infantile colic for both crying time and sleep disturbances* (Table 5).

##### Nocturnal enuresis

Five reviews [26, 44, 46, 96, 98] explored the use of spinal manipulation in children and adolescents to improve symptoms associated with nocturnal enuresis. Most found that there was no significant effect, with one review [26] finding inconclusive results. No additional studies were captured in our descriptive synthesis. *Our descriptive synthesis suggests that there is very strong evidence of ‘no significant effect’ from spinal manipulation for managing symptoms of nocturnal enuresis in children and adolescents* (Table 5).

##### Otitis media

Three reviews [98, 104, 105] that met our requirements for inclusion in the descriptive synthesis investigated the effects of spinal manipulation in infants to improve symptoms associated with otitis media. One [98] found no significant effects and two reviews [104, 105] found inconclusive results. A small cohort study [81] showed a significant reduction in otitis media symptoms (temperature and redness and bulging appearance of tympanic membrane) in children post spinal manipulation with a hand held pressure applicator but their findings have not been replicated. One further study [78] explored the use of spinal manipulation for improving otitis media-related symptoms in infants, however as it was a feasibility study for a larger RCT, the analysis of between group results was not reported. Our *descriptive synthesis reveals ‘inconclusive’ findings with no strong evidence to support the use of spinal manipulation to manage otitis media* (Table 5).

##### Torticollis

One review [62] exploring the use of spinal manipulation in infants and children met our criteria for descriptive synthesis. This review explored the effects of spinal manipulation on eight different outcomes related to torticollis, revealing inconclusive findings for each outcome. A single study [70] was also included in our descriptive synthesis and showed that lateral flexion and head righting reactions were not significantly improved after treatment involving spinal manipulation. *Subsequently, our descriptive synthesis suggests ‘insufficient’ findings with no clear evidence to support the use of spinal manipulation in infants to manage impairments related to torticollis* (Table 5).

High quality evidence was not available to explore the effectiveness of spinal manipulation for KISS syndrome or upper cervical dysfunction.

#### Effects of spinal mobilisation in infants, children and adolescents

Four systematic reviews [25–27, 104] explored the effects of spinal *mobilisation* on paediatric populations to manage impairments related to asthma [26], ADHD [25], torticollis [27] and upper cervical dysfunction [104]. Three of the four reviews were included in the descriptive synthesis as only one study was reviewed on the topic of interest (upper cervical dysfunction) for Parnell, 2019, which meant that it was precluded from our descriptive synthesis. Four additional studies [60, 75, 80, 81] were also captured in the present scoping review, exploring the effects of spinal mobilisation on infants and children with infantile colic, otitis media, plagiocephaly (without torticollis) and upper cervical dysfunction respectively (Supplementary File 5).

##### Asthma

With only one review [26] included and showing inconclusive results for the use of spinal mobilisation to improve peak expiratory flow in children and adolescents with asthma, our *descriptive synthesis suggests that there is ‘insufficient’ evidence to make conclusions regarding the effectiveness of spinal mobilisation for managing asthma symptoms.* (Table 6).

**Table 6:** Summary results of descriptive synthesis with levels of evidence statement for spinal MOBILISATION to manage paediatric populations with a variety of conditions

**Conditions *(Population)***	**Levels of Evidence Statement**			**Adverse Events** Documented in reviews and studies included in the descriptive synthesis		
	Reviews	High-Quality Studies	Summary Statement			
				Original Report (Author & Year)	Adverse Event / (Practitioner Type)	Further cited by
**Spinal MOBILISATION**						
Asthma ^ *(C&A)*	Insufficient	-	Insufficient			
ADHD ^ *(C)*	Insufficient	-	Insufficient			
Otitis Media ^ *(I&C)*	-	Insufficient	Insufficient			
Torticollis ^ *(I)*	Insufficient	-	Insufficient			
Plagiocephaly ^ *(I)* (With no torticollis)	-	Insufficient	Insufficient			
Upper Cervical Dysfunction ^ *(I)*	-	Insufficient	Insufficient	Saedt (2018)	Mild: back soreness, irritability, poor feeding, mild distress, increased crying, increased head tilt, temporary vegetative responses after mobilisation including: - Flushing: 17.8% (14.03, 22.59) - Hyper-extension: 4.3% (2.49, 7.11) - Perspiration: 3.6% (2.01, 6.30) - Gastro-esophageal reflux: 0.3% (0.06, 1.82) - Short breathing pattern changes: 9.2% (6.39, 12.87)	Corso (2020) [73]

All findings presented in this table are a result of the descriptive synthesis presented in Supplementary File 5

High quality evidence was not available to explore the effectiveness of spinal mobilisation on individuals with the following conditions: adolescent idiopathic scoliosis (AIS), autism spectrum disorder (ASD), back/neck pain, breastfeeding difficulties, cerebral palsy (CP), dysfunctional voiding, headache, infantile colic, KISS syndrome, nocturnal enuresis

Populations: I – Infants, C – Children, A – Adolescents

All adverse events extracted from included systematic reviews, except those studies marked with * which have been extracted from individual studies. ^Additional high-quality research (e.g., RCTs) may be warranted

Insufficient: Insufficient high-quality evidence available on the topic and further research may be warranted

Inconclusive: Available evidence is inconclusive, and further research may be warranted

No Significant Effect: High-quality evidence suggests the intervention is not effective and should not be used in clinical practice

Significant Positive Effect: High-quality evidence suggests the intervention is effective and could be used when clinical reasoning supports its application

##### ADHD

A single systematic review [25] met our criteria for descriptive synthesis which explored the use of spinal mobilisation to improve outcomes for children with ADHD using the Connors Scale. No significant effects were found, and as there were no additional studies exploring the effects of spinal mobilisation on children with ADHD, our *descriptive synthesis reveals ‘insufficient’ evidence to draw conclusions regarding the use of spinal mobilisation for managing ADHD.* (Table 6).

##### Infantile colic

A single RCT [80] explored the effects of spinal mobilisation on crying time in infants with colic, showing positive effects in the medium term (8–10 days) but no significant effectives in the short term (0–6 days). Our *descriptive synthesis revealed ‘insufficient’ evidence to draw conclusions regarding the use of spinal mobilisation to improve infantile colic.* (Table 6).

##### Torticollis

A single systematic review [27] met our criteria for descriptive synthesis which explored the effectiveness of spinal mobilisation for improving cervical mobility and cranial symmetry using the Argenta scale with infants. Both outcomes were reported to be improved by spinal mobilisations in infants with torticollis. However, as there are no additional reviews or studies on the topic, our *descriptive synthesis reveals ‘insufficient’ evidence to draw conclusions regarding the use of spinal mobilisation to improve impairments associated with torticollis in infants.* (Table 6).

##### Plagiocephaly (without torticollis) and upper cervical dysfunction

A single RCT [60] revealed that spinal mobilisation of the neck may reduce treatment days for infants with plagiocephaly but had no significant effects on motor development. Further, a single low level (Level III-2) study [75], suggested that spinal mobilisation of the neck may improve active and spontaneous movement of the neck in infants with upper cervical dysfunction. *Our descriptive synthesis suggests that there is ‘insufficient’ evidence to draw conclusions regarding the use of spinal mobilisation with infants and children to improve outcomes related to plagiocephaly (without torticollis) or upper cervical dysfunction.* (Table 6).

### Adverse events associated with spinal manipulation and mobilisation

For both reviews and studies included in the present scoping review, there was limited reporting of adverse events which means the true incidence is unknown. Of the reviews and studies that did report on adverse events related to spinal manipulation and mobilisation of infants, children and adolescents, they varied from mild to severe in nature (Table 7). Table 7 provides a summary of the reporting behaviours from included articles and demonstrates that six systematic reviews, eight RCTs and five other studies did not report the adverse events associated with using spinal manipulation to manage paediatric populations for a variety of conditions. Six reviews and three RCTs reported that there were no adverse events from using spinal manipulation with paediatric populations. When adverse events were documented, the trend demonstrated in the RCT’s were mild, transitory pain or soreness. All adverse events have been extracted from the original articles and documented in Tables 5 and 6 beside the conditions being treated at the time of the adverse events. Most adverse events were associated with spinal manipulation, rather than mobilisation and most occurred in infants or children, with few noted in adolescent populations.

**Table 7:** Adverse event reporting practice of included reviews and studies

Adverse events not reported	Nil adverse events reported	Adverse events reported		
		Mild	Moderate	Severe
**Adverse events associated with spinal *manipulation***				
**Reviews**				
Alcantara (2015) L2b [55] Ernst (2009) L1a [43] Fairest (2019) L4 [29] Fry (2014) L3 [30] Karpouzis (2010) L3b [101] Kronau (2016) L2b [102]	Edwards (2019) L3b [28] Ferrance (2010) L1b [91] Huang (2011) L1a [46] Hondras (2005) L1a [45] Romano (2008) L3b [106] Vaughn (2012) L1b [109]	Alcantara (2011a) L1b [48] Carnes (2018) L1b [66] Corso (2020) L1b [73] Gleberzon (2012) L1b [96] Pohlman (2012) L1b [105] Theroux (2017) L1b [107] Vohra (2007) L1b [110]	Brand (2005) L1a [41] Glazener (2005) L1a [44] Green (2019) L1b [21] Hawk (2007) L1b [98] Vohra (2007) L1b [110]	Brand (2005) L1a [41] Corso (2020) L1b [73] Green (2019) L1b [21] Todd (2015) L1b [108] Vohra (2007) L1b [110]
**RCT’s**				
Bronfort (2001) LII [56] Browning (2008) LII [58] Dissing (2018) LII [63] Kachmar (2018) SII [74] Lynge (2021) LII [77] Nemett (2008) LII [83] Olafsdottir (2001) LII [85] Reed LII (1994) [87]	Miller, Newell & Bolton (2012) LII [80] Selhorst LII (2015) [89] Haugen LII (2011) [70]	Balon LII (1998) [49] Borusiak LII (2009) [52] Evans (2018) LII [67]		
**Other studies**				
Davies (2007) LIII-3 [53] Hayden (2003) LIII-3 [57] Lantz (2001) LIII-2 [59] Miller & Newell (2012) LIII-2 [71] Miller (2009) LIII-2 [68]		Alcantara (2009) LIII-3 [50] Sawyer (1999) LIII-2 [78]	Leboeuf (1991) LIII-3 [61]	
**Adverse events associated with spinal *mobilisation***				
**Reviews**				
Parnell (2019) L1b (104)	Brurberg (2019) L1b [62]	Ellwood (2020) L1b [27] Driehuis (2019) L1b [26] Corso (2020) L1b [73]	Corso (2020) L1b [73]	Corso (2020) L1b [73]
**RCT’s**				
	Cabrera-Martos (2016) LII [60]			
**Other studies**				
Zhang (2004) LSIII-2 [81]		Saedt (2018) LIII-2 [75]		

L1a – Systematic Review of RCTs; L1b – Systematic Review of RCTs and other studies; L2a – Systematic review of quasi-experimental studies, L2b – Systematic review of quasi-experimental and other lower-level studies; L3a – Systematic Review of comparable cohort studies; L3b – Systematic review of comparable cohort and other lower-level studies; L4a – Systematic review of descriptive studies; LII – RCT, LIII-1 – Pseudorandomised controlled trial, LIII-2 – Comparative study with concurrent controls, LIII-3 – Comparative study without concurrent controls

### Policies, regulations, position statements, practice guidelines and opinion papers

Ten policy and policy development statements [2, 22, 95, 115–121] were included in this systematic scoping review. Most were from the United States of America with three of seven policies from Australia [2, 69, 95]. Two policies recommended the use of spinal manipulation in infants, children and adolescents [117, 118]. The International Chiropractors Association [118] recommend the earliest possible evaluation, detection and correction (using spinal manipulative therapy) in infants to maximise normal growth and development. One policy [117] stated that spinal manipulation must only be performed to manage three conditions: (i) if there has been documented symptoms involving the spine, (ii) subluxations of the spine are evidenced with corresponding symptoms and therapy has a direct relationship with improving function and (iii) if manipulation is appropriate to restore function that has been compromised by illness or injury. Contrary to above recommendations, seven policies do not recommend the use of spinal manipulation in infants, children and adolescents with arguments stating that it is experimental, unproven and not medically necessary [2, 69, 115, 116, 119–121]. Two of these policies specifying age groups, with one stating that spinal manipulation should not be used on paediatric patients under the age of two years [22] and the other not recommending it under the age of 12 years [119]. Of the policies that do not support this form of treatment, most don’t specify the conditions it is not recommended for. In those that do, there is a general trend towards prohibiting use for non-musculoskeletal conditions including ADHD, ASD, asthma, infantile colic, nocturnal enuresis and otitis media.

There were 14 text and opinion papers included in this systematic scoping review. Six did not support the use of spinal manipulation in infants, children and adolescents with comments suggesting there is limited research within the area, with no satisfactory evidence, suggesting the risks outweigh the potential benefits [65, 69, 84, 88, 90, 93]. Two text and opinion papers suggest there is limited, however, growing evidence for the use of spinal manipulation and report that care should be taken when using this form of treatment for managing impairments in paediatric populations [82, 88]. Conversely, six text and opinion papers support the use of spinal manipulation as a form of treatment for paediatric clients [72, 76, 79, 94, 95, 97] arguing that most chiropractors use best practice evidence-based treatment techniques, and that spinal manipulation may be effective in treating the paediatric populations. One text and opinion paper stated they are disappointed by the temporary restriction in Australia and believe chiropractors should not be singled out in performing such treatment, with limited evidence of harm [95].

Two guidelines were included in this review, one from Australia and one from the United States of America. The Australian guideline [47] suggested clinicians should not recommend spinal manipulation in infants as evidence is inconclusive. Conversely, the Council of Chiropractic Practice [54] recommendation suggests chiropractic care (inclusive of spinal manipulation) may be indicated at any age group and care must be taken to select the most appropriate treatment technique along with parental education. However, it is important to note that this guideline was published prior to the interim legislation.

## Discussion

The primary aim of this systematic scoping review was to identify and map the available evidence regarding the effectiveness and harms of spinal manipulation and mobilisation of infants, children, and adolescents. Additionally, we aimed to identify and synthesise policies, regulations, position statements and practice guidelines informing the clinical application of spinal manipulation and mobilisation in paediatric populations. In relation to our first aim, this systematic scoping review revealed that spinal *manipulation* and *mobilisation* is being utilised clinically by a variety of health professionals to manage paediatric populations with nocturnal enuresis, otitis media, infantile colic, excessive crying, breastfeeding difficulties, headaches, CP, back/neck pain, AIS, ADHD, ASD, torticollis, asthma, KISS syndrome, and dysfunctional voiding. We utilised a levels-of-evidence approach in our descriptive synthesis and whilst some individual high-quality studies demonstrated positive effects from spinal manipulation and mobilisation for some conditions, there is no collective evidence using objective measures to explicitly support the application of spinal *manipulation* or *mobilisation* for any condition in paediatric populations, however, adverse events were reported. Our descriptive synthesis revealed very strong evidence that spinal *manipulation* has no significant effect on nocturnal enuresis. Whilst results from previously published systematic reviews were inconclusive, our descriptive synthesis of studies with high methodological quality suggests there is strong evidence that spinal *manipulation* has no significant effect on impairments related to asthma (pulmonary function) or headache. The evidence was inconclusive regarding the effectiveness of spinal *manipulation* for managing impairments related to AIS, ASD, back/neck pain (acute and chronic) and CP in children and adolescents. Additionally, the evidence was inconclusive regarding the effectiveness of spinal *manipulation* for managing impairments and symptoms related to breastfeeding difficulties, infantile colic (excessive crying and sleep disturbances), and otitis media in infants and children. There was insufficient evidence to report on the effectiveness of spinal *manipulation* for infants and children with torticollis and ADHD. Further, there is insufficient evidence to determine the effectiveness of spinal *mobilisation* on paediatric populations for managing any condition.

To further address our first aim, we explored the adverse events/harms associated with spinal manipulation and/or mobilisation in paediatric populations. The findings in the present systematic scoping review revealed that there is limited reporting of adverse events in the included systematic reviews and studies, with six reviews, eight RCTs and five other studies making no mention of adverse events or harms associated with their spinal manipulation intervention of focus (Table 7). Although some of these articles were published before 2010, those RCT’s published after 2010, have failed to comply with the internationally accepted updated CONSORT guidelines which urges authors to be completely transparent in their reporting of harms [122]. Four systematic reviews focused specifically on adverse events and harms associated with treatment of infants, children and adolescents involving spinal manipulation and mobilisations and revealed that adverse events ranged from mild – requiring self or parent care only, to severe – for example, death. All adverse events that were extracted from our included articles are documented in the data extraction table (Supplementary File 3) and these have been summarised according to the conditions being managed in the studies/reviews reporting adverse events (Tables 5 and 6). With respect to potential harms, our review identified under-reporting of adverse events across both reviews and studies (Table 7), impacting our ability to draw firm conclusions regarding the safety of spinal manipulation and mobilisation in infants, children and adolescents and this finding aligns with conclusions expressed in previous reviews [21, 26]. Due to the limited reporting of adverse events in many studies, the true incidence remains unknown [104, 110]. However, we would like to highlight that although there have been some reports (studies and reviews) demonstrating improvement in mild transient adverse symptoms (e.g., muscle soreness and tightness [21, 44, 73, 105, 110], anxiety [44] and increased crying [73] after receiving treatment with spinal manipulation), there has also been reports of more serious adverse events such as severe headache [21, 52, 108, 110], loss of consciousness [21, 52, 110], poor coordination and unsteady gait [110], clonus at rest [110], reflex apnoea [41], facial weakness [108], diplopia ataxia [108], acute respiratory decompensation [110] and urinary urgency and frequency [110]. Whilst most adverse events are mild and transient, the most severe adverse events from spinal manipulation noted in the literature are progressive neuromuscular deficits leading to quadriplegia (later improving to paraplegia post-surgically), missed or delayed diagnoses (e.g., spinal cord astrocytoma and congenital occipitalisation), subarachnoid haemorrhage and death. (Table 5). Related to this issue, it is evident that there is an important difference in the practice of clinical reasoning for spinal manipulation across the professions, with some advocating for using a directional ‘thrust’ to move a spinal segment back into alignment (i.e., adjustment) [5] and some professions using a HVLA passive thrust to the spinal joint *within* its anatomical limit [6]. Whilst the research team were able to confirm ‘spinal manipulation’ as the treatment technique in this review during study selection, very little detail was given to describe the way the spinal segment or joint was being manipulated. Future publications regarding spinal manipulation should explicitly describe the form of manipulation being undertaken, as it is entirely possible that the effectiveness and safety could vary between techniques. Whilst the prevalence of documented adverse events from spinal manipulation and mobilisation appears to be relatively low, the most severe adverse events were reported in infants during treatment of conditions where it is difficult to monitor the structures being impacted due to the small anatomical size of infants and where there are other effective evidence-based intervention options (e.g., torticollis [27] infantile colic [114]). Notably, there were less adverse events reported for spinal *mobilisation* in paediatric populations, with one review article [73] identifying severe adverse events such as rib fractures and missed significant diagnoses (e.g., spinal cord astrocytoma), however, our scoping review also identified far less studies or reviews exploring the use of spinal mobilisation (as opposed to spinal manipulation) in paediatric populations from which to extract this data.

Most studies within the included reviews came from low levels of evidence such as case studies or case series, which were not included as individual studies for our current systematic scoping review as we felt they were too low in the levels of evidence hierarchy to provide additional meaningful results regarding effectiveness. The inclusion of adverse events extracted from lower levels of research published in the included systematic reviews, has provided important safety related information for readers to consider. However, since most of the literature is based on low-level studies such as case reports, it is not safe to assume that their conclusions can be generalised to larger or alternate populations. Health professionals would benefit from further training, either as graduates or in entry-level programs, to better understand levels of evidence to assist with interpretation of research, to inform their choice of treatment techniques and to guide design of future research, should they choose to do it. Consistent with the lower levels of evidence and methodological quality of studies, it was noted that very few studies reported on the clinometric properties of the outcome measures utilised, and we recommend future research on this topic to include references regarding the reliability, validity, utility, and efficacy of outcome measures used to explore effectiveness and to improve credibility of study findings. Healthcare professionals and researchers should be aware of the reliability, validity, and responsiveness of assessment tools and outcome measures to assist in their clinical reasoning, instrument selection and interpretation of clinical or research results. Further evaluation of these factors must be completed in future research to assist with interpretation of the collective findings on this important topic.

Regarding our final aim, we have identified that most policy and policy development statements included in this systematic scoping review were developed in the United States of America, many by third party payers, and only three published in Australia. This highlights the need for more policies globally across all professions who are performing spinal manipulation and mobilisation with paediatric populations. Evidence-based guidelines and policy or position statements are needed to guide health professionals on the appropriateness of spinal manipulation and mobilisation to manage a variety of conditions for which paediatric clients commonly present for care. This is particularly important considering our comprehensive review and descriptive synthesis did not determine spinal manipulation or mobilisation to be effective for treating any condition examined (Tables 5 and 6), albeit with limited research to examine for spinal mobilisation. Whilst not captured by our inclusion criteria (due to being published in Dutch language), the Netherlands have produced four factsheets [123–126] on diagnostics and therapeutics in infants (0–1 year) and children (1–18 years) to guide physiotherapy practice for using manual therapy in paediatric populations, and their guidance is congruent with the findings of the present scoping review.

The findings from the present systematic scoping review align with the findings from the previous work by Green [21] for the Safer Care Victoria report on Chiropractic spinal manipulation of children under 12: Independent review [2] and the recent findings from Cote and colleagues [14]. Green (2019) [21] explored the effectiveness and safety of spinal manipulation (but not mobilisation) in children under 12 years for any condition or impairment, irrespective of the profession providing treatment. The outcome of Greens’ review was that spinal manipulation should not (due to a lack of evidence and potential risk of harm) be recommended for management of paediatric clients with; headache, asthma, otitis media, cerebral palsy, hyperactivity disorders or torticollis, however, they suggested that there may be some (although unlikely) benefits of spinal manipulation in the management of infantile colic and nocturnal enuresis. The findings from the present systematic scoping review differ slightly as our descriptive synthesis using a levels of evidence approach, extends these conclusions as we also found very strong evidence that spinal manipulation is not effective for managing nocturnal enuresis. Further, we found the evidence to be ‘inconclusive’ for managing excessive crying and sleep in infants with infantile colic. Our findings, much like those of Cote [14] suggest that evidence is lacking to support the use of spinal manipulative therapy to treat non-musculoskeletal disorders, undermining the validity of the theory that spinal manipulation has physiological effects on the organs and their function. The findings from the present systematic scoping review add to the Safer Care Victoria review in the following ways: (i) exploring both spinal manipulation and mobilisation; (ii) inclusion of paediatric patients up to the age of 18 years; (iii) inclusion of various study designs except individual case reports and case studies; (iv) investigated policies, guidelines and laws supporting or prohibiting the use of spinal manipulation or mobilisation. It should be noted that many of the policies identified in this scoping review from the USA were reimbursement policies and there remains a need in the USA for professional associations to establish position statements and treatment guidelines.

A challenge that we faced in screening, appraising, data extracting and synthesising the included articles, was the lack of detailed descriptions of therapeutic techniques being applied (i.e., spinal manipulation and mobilisation techniques) on infants, children and adolescents; a concern raised in a previous review on the topic [26]. Relevant and necessary information regarding the treatment technique used were often not clearly stated. Due to the underreporting of specific techniques, we had to exclude numerous reviews on the basis that we were uncertain of the treatment technique being applied. Consequently, this has limited our ability to draw conclusions regarding effectiveness of specific treatment techniques, particularly spinal mobilisation. These findings align with the findings of other reviews who also highlight the importance of increasing the methodological quality to describe intervention techniques completed by the practitioner [26, 104]. To assist with capturing a wider sample of studies in future reviews, it would be beneficial for researchers to include details describing the exact treatment technique, the number and duration of treatments patients received, and the healthcare providers experience and training.

A strength of this systematic scoping review includes the wide breadth of searches undertaken. Several major databases were searched with a detailed search strategy and with a broad, yet specific inclusion criteria to ensure the scope of existing literature was included. Hand searching of reference lists for all included studies and reviews was undertaken to ensure all relevant literature was captured for this systematic scoping review. The study selection, data extraction process and critical appraisal was completed independently by two reviewers to reduce the risk of reviewer error or bias and a third reviewer was utilised to validate data extraction and provide consensus for critical appraisal. Our review sets itself apart from previous research by focusing on both spinal manipulation and mobilisation, as well as including participants from birth to 18 years of age. Exclusion of individual case studies and case reports allowed for conclusions to be based on higher levels of evidence and this was particularly important when the collective evidence from systematic reviews were inconclusive.

Due to the inclusion of systematic reviews, there were several primary studies included more than once, potentially leading to overrepresentation of individual studies, which may have biased the interpretation of the results. Whilst we independently descriptively synthesised the individual study outcomes (from high quality studies); the synthesis findings may have been influenced by one study population or methods if they had completed multiple investigations, and therefore, one population sample may have biased our analysis. On the occasion that this was likely (*n* = 2 conditions), we have highlighted this to the reader (see Supplementary File 5). As there was limited overlap and because many reviews included low levels of evidence, data extraction, critical appraisal and descriptive synthesis was completed for both the studies and the reviews independently before applying the levels of evidence approach to our descriptive synthesis. The overarching limitation of our findings is the high representation of non-RCT (e.g., observational studies, case studies) in the included reviews, leaving in some cases our synthesis and conclusions to be based on the collective findings from lower levels of evidence. A further limitation of this scoping review is the use of a descriptive synthesis employing a levels of evidence approach based on quality and quantity of studies without consideration of sample size, rather than a meta-analysis which meant we were unable to determine effect sizes.

Despite the current limitations, this systematic scoping review provides information to build awareness regarding the available evidence for safety and effectiveness of spinal manipulation and mobilisation in paediatric populations (birth up to 18 years) and these findings can be used to guide more impairment focused quantitative analysis in future meta-analyses. The results of this systematic scoping review will also help to inform the future development of a shared position statement between the IFOMPT and IOPTP to guide clinical practice.

## Conclusions

The present systematic scoping review revealed spinal manipulation and mobilisation are utilised clinically by a variety of health professionals to manage many different musculoskeletal and non-musculoskeletal impairments for paediatric populations. A broad descriptive synthesis of the collective evidence (using a levels-of-evidence approach) did not demonstrate evidence to explicitly support spinal *manipulation* or *mobilisation* as an effective intervention for any condition in paediatric populations with mild to severe adverse events reported. Strong to very strong evidence exists to suggest that spinal *manipulation* is not effective for managing asthma, headache or nocturnal enuresis whereas, there was inconclusive or insufficient evidence for all other conditions explored. There is insufficient evidence to determine the effectiveness of spinal *mobilisation* for treating paediatric populations with any condition, with some mild adverse responses reported. Despite spinal manipulation and mobilisation being used to treat infants, children, and adolescents internationally, there is a lack of conclusive high-level evidence providing positive (i.e., favourable) results with paediatric populations. More high-level clinically reasoned RCT’s, expressing the magnitude of effect from spinal manipulation and mobilisation are needed, to further allow exploration of the safety and effectiveness of these interventions with infants, children and adolescents, for further conclusions to be drawn. Future research should include strict monitoring and recording of adverse events to determine true risks and could start with small long term RCTs. If evidence was accumulating for a given condition, a large multicentre RCT would be beneficial. In addition, future research in this field, should provide *detailed* information about the therapeutic technique, the clinical reasoning, and theoretical underpinnings for its use, particularly in non-musculoskeletal conditions. Currently most research informing the results of this systematic scoping review are based on chiropractic interventions. Research regarding physiotherapy methods for mobilisation and manipulation for some conditions (e.g., back and neck pain/stiffness) in older children and adolescents is warranted as it remains a gap in the literature.

## Supplementary Information

**Additional file 1: Supplementary File 1. **Search Strategy 

**Additional file 2: Supplementary File 2. **Critical Appraisal Consensus Scores

**Additional file 3: Supplementary File 3. **Data Extraction 

**Additional file 4: Supplementary File 4. **Matrix with RCT's and other studies included across all systematicreviews 

**Additional file 5: Supplementary File 5. **Descriptive Synthesis

## Data Availability

All data generated or analysed during this study are tabulated in this published article [and its supplementary information files].
