# Supplementary material for: Spinal manipulation and mobilisation in the treatment of infants, children, and adolescents: a systematic scoping review
Source: BMC Pediatr. 2022 Dec 19;22:721. doi: 10.1186/s12887-022-03781-6 (PMC9762100; doi:10.1186/s12887-022-03781-6)
Supplement: Supplementary file 1 — Additional file 1: Supplementary File 1. Search Strategy [file 12887_2022_3781_MOESM1_ESM.docx]

| Supplementary File 1 Search Strategy | |
| --- | --- |
| Databases | Search Strategy |
| PubMed, Embase, CINAHL and Cochrane | (“Infant*” OR “baby” OR “neonate” OR “newborn” OR “toddler” OR “kid” OR “Adolescent*” OR “teen*” OR “juvenile” OR “youth” OR “young” OR “paediatric*” OR “pediatric*” OR “child*”) AND (“spin* manipulation” OR “Spin* Mobilisation*” OR “Spin* Mobilization*” OR “Spin*Adjustment*” OR “Spin* Manual therapy” OR “high velocity low amplitude thrust” OR “HVLA” OR “Musculoskeletal of the spine” OR “Spinal musculoskeletal” OR “manual therapy of the spine” OR “cervical manual therapy” OR “thoracic manual therapy” OR “ lumbar manual therapy” OR “manual therapy of the lumbar spine” OR “manual therapy of the thoracic spine” OR “manual therapy of the cervical spine” OR “spinal osteopath*” OR “osteopath* of the spine” OR “osteopath* of the cervical spine” OR “osteopath* of the thoracic spine” OR “osteopath* of the lumbar spine” OR “Chiro*” OR “Spinal manipulative therapy”) AND (“Cry” OR “Crying” OR “Unsettled” OR “distressed” OR “Colic” OR “gastroesophageal reflux” OR “reflux” OR “Extension Posturing”  OR “posture” OR “Stiff Neck” OR “Stiff Back” OR “Stiff spine” OR “Enuresis” OR “Headache*” OR “dizziness” OR “Asthma” OR “dyspnea” OR “Otitis Media” OR “Cerebral Palsy” OR “Hyperactivity” OR “Torticollis” OR “cervical dystonia” OR “Acute wryneck” OR “Acute wry neck” OR “Whiplash” OR “whiplash” OR “Jaw Pain” OR “Neck Pain” OR “Back Pain” OR “Mobility” OR “Range of Motion” OR “Scoliosis” OR “posture changes” OR “breastfeeding” OR “Feeding” OR “Latching” OR “Sleep*” OR “Wellbeing” OR “preventative care” OR “kinetic imbalance due to suboccipital strain” OR “KISS syndrome” OR “autism” OR “constipation” OR “gastrointestinal” OR “GI” OR “allergy” OR “non musculoskeletal”) AND (“Review” OR “Systematic Review” OR “Meta-analysis” OR “RCT” OR “Randomised Controlled Trial” OR “ Randomized Controlled Trial” OR “Randomised Control Trial” OR “Randomized Control Trial” OR “Clinical Trial” OR “Trial” OR “Observational” OR “Effec*” OR “Adverse” OR  “harm*” OR “best practice” OR “recommendations” OR “consensus” OR “risk*” OR “critical overview” OR “Evidence” OR “treatment”) |
