# Supplementary material for: Spinal manipulation and mobilisation in the treatment of infants, children, and adolescents: a systematic scoping review
Source: BMC Pediatr. 2022 Dec 19;22:721. doi: 10.1186/s12887-022-03781-6 (PMC9762100; doi:10.1186/s12887-022-03781-6)
Supplement: Supplementary file 2 — Additional file 2: Supplementary File 2. Critical Appraisal Consensus Scores [file 12887_2022_3781_MOESM2_ESM.docx]

| **SUPPLEMENTARY FILE 2** Critical Appraisal Consensus Scores | | | | | | | | | | | | | | | | | | | | | | | | | | | | | | | | | | | | | | | | | | | | | | | | | | | | | | | | | |  |
| --- | --- | --- | --- | --- | --- | --- | --- | --- | --- | --- | --- | --- | --- | --- | --- | --- | --- | --- | --- | --- | --- | --- | --- | --- | --- | --- | --- | --- | --- | --- | --- | --- | --- | --- | --- | --- | --- | --- | --- | --- | --- | --- | --- | --- | --- | --- | --- | --- | --- | --- | --- | --- | --- | --- | --- | --- | --- | --- |
| **Critical Appraisal Consensus Scores for Systematic Reviews** | | | | | | | | | | | | | | | | | | | | | | | | | | | | | | | | | | | | | | | | | | | | | | | | | | | | | | | | | |  |
| **Authors (Year)** | | | **JBI Systematic Reviews and Research Synthesis** | | | | | | | | | | | | | | | | | | | | | | | | | | | | | | | | | | | | | | | | | | | | | | | | | | | | | |  |  |
|  | | | Review question stated | | | | | Inclusion Criteria | | | | | Search Strategy | | | | Sources/ Resources | | | | | Criteria to appraise | | | | | Critical appraisal | | | | | Method to minimise errors in data extraction | | | | | | Methods to combine studies | | | | | Publication Bias | | | | | | Recommendations for policy/practice supported | | | | | Specific derivative for new research | | | Critical Appraisal Score |  |
| Alcantara, et al. (2011a) | | | 0 | | | | | 1 | | | | | 0 | | | | 1 | | | | | 0 | | | | | 0 | | | | | 0 | | | | | | 1 | | | | | 0 | | | | | | 0 | | | | | 0 | | | 3/11 |  |
| Alcantara, et al. (2011b) | | | 1 | | | | | 1 | | | | | 1 | | | | 1 | | | | | 0 | | | | | 0 | | | | | 1 | | | | | | 1 | | | | | 0 | | | | | | 1 | | | | | 1 | | | 8/11 |  |
| Alcantara, et al. (2015) | | | 0 | | | | | 0 | | | | | 1 | | | | 1 | | | | | 0 | | | | | 0 | | | | | 0 | | | | | | 1 | | | | | 0 | | | | | | 1 | | | | | 0 | | | 4/11 |  |
| Brand, et al. (2005) | | | 1 | | | | | U | | | | | 1 | | | | 1 | | | | | 0 | | | | | 0 | | | | | 0 | | | | | | 1 | | | | | 1 | | | | | | 1 | | | | | 1 | | | 7/11 |  |
| Bronfort, et al. (2010) | | | 1 | | | | | 1 | | | | | U | | | | 1 | | | | | 1 | | | | | 1 | | | | | U | | | | | | 1 | | | | | 0 | | | | | | 1 | | | | | 0 | | | 7/11 |  |
| Brurberg, et al. (2019) | | | 0 | | | | | 1 | | | | | 1 | | | | 1 | | | | | 1 | | | | | 0 | | | | | 0 | | | | | | 1 | | | | | 0 | | | | | | 1 | | | | | 0 | | | 6/11 |  |
| Carnes, et al. (2018) | | | 1 | | | | | 1 | | | | | 1 | | | | 1 | | | | | 1 | | | | | 1 | | | | | 1 | | | | | | 1 | | | | | 1 | | | | | | 1 | | | | | 1 | | | 11/11 |  |
| Clar, et al. (2014) | | | 1 | | | | | 1 | | | | | 1 | | | | 1 | | | | | 1 | | | | | 1 | | | | | 1 | | | | | | 1 | | | | | 1 | | | | | | 0 | | | | | 1 | | | 10/11 |  |
| Corso, et al. (2020) | | | 1 | | | | | 1 | | | | | 1 | | | | 1 | | | | | 1 | | | | | 1 | | | | | 1 | | | | | | 1 | | | | | 1 | | | | | | 1 | | | | | 1 | | | 11/11 |  |
| Dobson, et al. (2012) | | | 1 | | | | | 1 | | | | | 1 | | | | 1 | | | | | 1 | | | | | 1 | | | | | 1 | | | | | | 1 | | | | | 1 | | | | | | 0 | | | | | 1 | | | 10/11 |  |
| Driehuis, et al. (2019) | | | 1 | | | | | 1 | | | | | 1 | | | | 1 | | | | | 1 | | | | | 1 | | | | | 1 | | | | | | 1 | | | | | 1 | | | | | | 0 | | | | | 1 | | | 10/11 |  |
| Edwards and Miller (2019) | | | 1 | | | | | 1 | | | | | 1 | | | | 1 | | | | | 1 | | | | | 0 | | | | | 0 | | | | | | 1 | | | | | 0 | | | | | | 1 | | | | | 1 | | | 8/11 |  |
| Ellwood, et al. (2020) | | | 1 | | | | | 1 | | | | | 1 | | | | 1 | | | | | 1 | | | | | 1 | | | | | 1 | | | | | | 1 | | | | | 0 | | | | | | 1 | | | | | 1 | | | 10/11 |  |
| Ernst (2009) | | | 1 | | | | | 1 | | | | | 0 | | | | 1 | | | | | 0 | | | | | 0 | | | | | 0 | | | | | | 1 | | | | | 0 | | | | | | 1 | | | | | 1 | | | 6/11 |  |
| Fairest, et al. (2019) | | | 0 | | | | | 0 | | | | | 1 | | | | 0 | | | | | 0 | | | | | 0 | | | | | 0 | | | | | | 1 | | | | | 0 | | | | | | 1 | | | | | 0 | | | 3/11 |  |
| Ferrance and Miller (2010) | | | 1 | | | | | 0 | | | | | 0 | | | | 0 | | | | | 0 | | | | | 0 | | | | | 0 | | | | | | 0 | | | | | 0 | | | | | | 1 | | | | | 1 | | | 3/11 |  |
| Fry (2014) | | | 1 | | | | | 1 | | | | | 1 | | | | 1 | | | | | 0 | | | | | 0 | | | | | 0 | | | | | | 1 | | | | | 0 | | | | | | 0 | | | | | 0 | | | 5/11 |  |
| Glazener, et al. (2005) | | | 1 | | | | | 1 | | | | | 1 | | | | 1 | | | | | 1 | | | | | 1 | | | | | 1 | | | | | | 1 | | | | | 0 | | | | | | 1 | | | | | 1 | | | 10/11 |  |
| Gleberzon, et al. (2012) | | | 1 | | | | | 1 | | | | | 1 | | | | 1 | | | | | 1 | | | | | 1 | | | | | 1 | | | | | | 1 | | | | | 0 | | | | | | 1 | | | | | 1 | | | 10/11 |  |
| Green, et al. (2019) | | | 1 | | | | | 1 | | | | | 1 | | | | 1 | | | | | 1 | | | | | 0 | | | | | 1 | | | | | | 1 | | | | | 1 | | | | | | 1 | | | | | 1 | | | 10/11 |  |
| Hawk, et al. (2007) | | | 1 | | | | | U | | | | | 1 | | | | 1 | | | | | 1 | | | | | 1 | | | | | 0 | | | | | | 1 | | | | | 0 | | | | | | 1 | | | | | 1 | | | 8/11 |  |
| Hawk, et al. (2019) | | | 1 | | | | | 1 | | | | | 1 | | | | 1 | | | | | 1 | | | | | 1 | | | | | 1 | | | | | | 1 | | | | | 1 | | | | | | 1 | | | | | 1 | | | 11/11 |  |
| Hondras, et al. (2005) | | | 1 | | | | | 1 | | | | | 1 | | | | 1 | | | | | 1 | | | | | 1 | | | | | 1 | | | | | | 1 | | | | | 0 | | | | | | 1 | | | | | 1 | | | 10/11 |  |
| Humphreys (2010) | | | 1 | | | | | 1 | | | | | 0 | | | | 1 | | | | | 0 | | | | | 0 | | | | | 0 | | | | | | 0 | | | | | 0 | | | | | | 1 | | | | | 1 | | | 5/11 |  |
| Huang, et al. (2011) | | | 1 | | | | | 1 | | | | | 0 | | | | 1 | | | | | 0 | | | | | 0 | | | | | 1 | | | | | | 1 | | | | | 0 | | | | | | 1 | | | | | 1 | | | 7/11 |  |
| Karpouzis, et al. (2010) | | | 1 | | | | | 1 | | | | | 1 | | | | 1 | | | | | 1 | | | | | 0 | | | | | 0 | | | | | | 0 | | | | | 1 | | | | | | 1 | | | | | 1 | | | 7/11 |  |
| Kronau, et al. (2016) | | | 1 | | | | | 1 | | | | | 1 | | | | 1 | | | | | 1 | | | | | 1 | | | | | 1 | | | | | | 1 | | | | | 1 | | | | | | 1 | | | | | 1 | | | 11/11 |  |
| Lucassen (2010) | | | 1 | | | | | 1 | | | | | 1 | | | | 1 | | | | | 1 | | | | | 0 | | | | | 0 | | | | | | 1 | | | | | 0 | | | | | | 1 | | | | | 0 | | | 7/11 |  |
| Parnell, et al. (2019) | | | 1 | | | | | 1 | | | | | 1 | | | | 1 | | | | | 1 | | | | | 1 | | | | | 1 | | | | | | 1 | | | | | 1 | | | | | | 1 | | | | | 1 | | | 11/11 |  |
| Pohlman and Holton-Brown (2012) | | | 1 | | | | | 1 | | | | | 1 | | | | 1 | | | | | 1 | | | | | 1 | | | | | 0 | | | | | | 1 | | | | | 0 | | | | | | 0 | | | | | 1 | | | 8/11 |  |
| Romano and Negrini (2008) | | | 1 | | | | | 1 | | | | | 1 | | | | 1 | | | | | 0 | | | | | 0 | | | | | 0 | | | | | | 1 | | | | | 0 | | | | | | 0 | | | | | 1 | | | 6/11 |  |
| Theroux, et al. (2017) | | | 1 | | | | | 1 | | | | | U | | | | 1 | | | | | 1 | | | | | 1 | | | | | 1 | | | | | | 1 | | | | | 1 | | | | | | 1 | | | | | 1 | | | 10/11 |  |
| Todd, et al. (2015) | | | 1 | | | | | 1 | | | | | 1 | | | | 1 | | | | | 0 | | | | | 0 | | | | | 0 | | | | | | 1 | | | | | 0 | | | | | | 1 | | | | | 1 | | | 7/11 |  |
| Vaughn, et al. (2012) | | | 1 | | | | | 1 | | | | | 1 | | | | 1 | | | | | 1 | | | | | 1 | | | | | 1 | | | | | | 1 | | | | | 1 | | | | | | 1 | | | | | 1 | | | 11/11 |  |
| Vohra, et al. (2007) | | | 1 | | | | | 1 | | | | | 1 | | | | 1 | | | | | 0 | | | | | 0 | | | | | 1 | | | | | | 0 | | | | | 0 | | | | | | 1 | | | | | 1 | | | 7/11 |  |
| Scoring: 0 – ‘no’, 1 – ‘yes’, U – ‘unclear’. | | | | | | | | | | | | | | | | | | | | | | | | | | | | | | | | | | | | | | | | | | | | | | | | | | | | | | | | | |  |
| **Critical Appraisal Consensus Scores for Studies** | | | | | | | | | | | | | | | | | | | | | | | | | | | | | | | | | | | | | | | | | | | | | | | | | | | | | | | | | |  |
| **Author (Year)** | **Screening Questions** | | | **MMAT (Randomised study)** | | | | | | | | | | | | | | **MMAT (Non-randomised study)** | | | | | | | | | | | | | **MMAT (Descriptive Studies)** | | | | | | | | | | | | | **MMAT (Mixed Methods Studies)** | | | | | | | | | | |  | | | |
|  | S1 | S2 | | 2.1 | 2.2 | | | | | 2.3 | 2.4 | | | 2.5 | | | | 3.1 | | 3.2 | | | 3.3 | | | 3.4 | | | 3.5 | | 4.1 | | 4.2 | | | 4.3 | | | 4.4 | | 4.5 | | | 5.1 | | | 5.2 | | 5.3 | | 5.4 | | 5.5 | | Critical Appraisal Score | | | |
| Alcantara et al. (2009) | 1 | 1 | |  |  | | | | |  |  | | |  | | | |  | |  | | |  | | |  | | |  | | 1 | | 1 | | | 1 | | | 0 | | 1 | | |  | | |  | |  | |  | |  | | 6/7 | | | |
| Balon (1998) | 1 | 1 | | 1 | 1 | | | | | 1 | 1 | | | 1 | | | |  | |  | | |  | | |  | | |  | |  | |  | | |  | | |  | |  | | |  | | |  | |  | |  | |  | | 7/7 | | | |
| Borusiak (2009) | 0 | 1 | | 1 | 1 | | | | | 1 | 1 | | | 0 | | | |  | |  | | |  | | |  | | |  | |  | |  | | |  | | |  | |  | | |  | | |  | |  | |  | |  | | 5/7 | | | |
| Bronfort et al. (2001) | 1 | 1 | | 1 | 0 | | | | | 0 | 1 | | | 1 | | | |  | |  | | |  | | |  | | |  | |  | |  | | |  | | |  | |  | | |  | | |  | |  | |  | |  | | 5/7 | | | |
| Browning et al. (2008) | 1 | 1 | | 1 | 1 | | | | | 1 | 1 | | | 1 | | | |  | |  | | |  | | |  | | |  | |  | |  | | |  | | |  | |  | | |  | | |  | |  | |  | |  | | 7/7 | | | |
| Cabrera-Martos et al. (2016) | 1 | 1 | | 1 | 1 | | | | | 1 | 1 | | | 1 | | | |  | |  | | |  | | |  | | |  | |  | |  | | |  | | |  | |  | | |  | | |  | |  | |  | |  | | 7/7 | | | |
| Davies and Jamieson (2007) | 1 | 1 | |  |  | | | | |  |  | | |  | | | |  | |  | | |  | | |  | | |  | | 1 | | 1 | | | 1 | | | 1 | | 0 | | |  | | |  | |  | |  | |  | | 6/7 | | | |
| Dissing et al. (2018) | 1 | 1 | | 1 | 1 | | | | | 0 | 1 | | | 1 | | | |  | |  | | |  | | |  | | |  | |  | |  | | |  | | |  | |  | | |  | | |  | |  | |  | |  | | 6/7 | | | |
| Evans et al. (2018) | 1 | 1 | | 1 | 1 | | | | | 1 | 0 | | | 1 | | | |  | |  | | |  | | |  | | |  | |  | |  | | |  | | |  | |  | | |  | | |  | |  | |  | |  | | 6/7 | | | |
| Haugen et al. (2011) | 1 | 1 | | 1 | 0 | | | | | 0 | 1 | | | 1 | | | |  | |  | | |  | | |  | | |  | |  | |  | | |  | | |  | |  | | |  | | |  | |  | |  | |  | | 5/7 | | | |
| Hayden et al. (2003) | 1 | 1 | |  |  | | | | |  |  | | |  | | | | 1 | | 1 | | | 1 | | | U | | | 1 | |  | |  | | |  | | |  | |  | | |  | | |  | |  | |  | |  | | 6/7 | | | |
| Kachmar, et al. (2018) | 1 | 1 | | 1 | 1 | | | | | 1 | 1 | | | 1 | | | |  | |  | | |  | | |  | | |  | |  | |  | | |  | | |  | |  | | |  | | |  | |  | |  | |  | | 7/7 | | | |
| Lantz and Chen (2001) | 1 | 1 | |  |  | | | | |  |  | | |  | | | | 1 | | 1 | | | 1 | | | U | | | 1 | |  | |  | | |  | | |  | |  | | |  | | |  | |  | |  | |  | | 5/7 | | | |
| Lebouef (1991) | 1 | 1 | | 0 | 0 | | | | | 1 | 0 | | | U | | | |  | |  | | |  | | |  | | |  | |  | |  | | |  | | |  | |  | | |  | | |  | |  | |  | |  | | 3/7 | | | |
| Lynge et al. (2021) | 1 | 1 | | 1 | 1 | | | | | 1 | 0 | | | 1 | | | |  | |  | | |  | | |  | | |  | |  | |  | | |  | | |  | |  | | |  | | |  | |  | |  | |  | | 6/7 | | | |
| Miller and Beinfield (2008) | 1 | 1 | |  |  | | | | |  |  | | |  | | | |  | |  | | |  | | |  | | |  | | 1 | | 1 | | | 1 | | | 1 | | 1 | | |  | | |  | |  | |  | |  | | 7/7 | | | |
| Miller and Newell (2012) | 1 | 1 | |  |  | | | | |  |  | | |  | | | | 1 | | 1 | | | U | | | 0 | | | 1 | |  | |  | | |  | | |  | |  | | |  | | |  | |  | |  | |  | | 5/7 | | | |
| Miller, Newell and Bolton (2012) | 1 | 1 | | 1 | 1 | | | | | 1 | 0 | | | 0 | | | |  | |  | | |  | | |  | | |  | |  | |  | | |  | | |  | |  | | |  | | |  | |  | |  | |  | | 5/7 | | | |
| Miller and Phillips (2009) | 1 | 1 | |  |  | | | | |  |  | | |  | | | |  | |  | | |  | | |  | | |  | | 1 | | 1 | | | 1 | | | 0 | | 1 | | |  | | |  | |  | |  | |  | | 5/7 | | | |
| Nemett (2008) | 1 | 1 | | 1 | 1 | | | | | 0 | U | | | 0 | | | |  | |  | | |  | | |  | | |  | |  | |  | | |  | | |  | |  | | |  | | |  | |  | |  | |  | | 4/7 | | | |
| Olafsdottir et al. (2001) | 1 | 1 | | 1 | 1 | | | | | 1 | 1 | | | 1 | | | |  | |  | | |  | | |  | | |  | |  | |  | | |  | | |  | |  | | |  | | |  | |  | |  | |  | | 7/7 | | | |
| Reed (1994) | 1 | 1 | | 0 | 0 | | | | | 0 | 0 | | | 0 | | | |  | |  | | |  | | |  | | |  | |  | |  | | |  | | |  | |  | | |  | | |  | |  | |  | |  | | 2/7 | | | |
| Saedt et al. (2018) | 1 | 1 | |  |  | | | | |  |  | | |  | | | |  | |  | | |  | | |  | | |  | | 1 | | 0 | | | 1 | | | 0 | | 1 | | |  | | |  | |  | |  | |  | | 5/7 | | | |
| Selhorst and Selhorst (2015) | 1 | 1 | | 1 | 1 | | | | | 1 | 1 | | | 1 | | | |  | |  | | |  | | |  | | |  | |  | |  | | |  | | |  | |  | | |  | | |  | |  | |  | |  | | 7/7 | | | |
| Sawyer et al. (1999) | 1 | 1 | | 1 | 1 | | | | | 0 | 1 | | | 1 | | | |  | |  | | |  | | |  | | |  | |  | |  | | |  | | |  | |  | | |  | | |  | |  | |  | |  | | 6/7 | | | |
| Thiel et al. (2007) | 1 | 1 | |  |  | | | | |  |  | | |  | | | |  | |  | | |  | | |  | | |  | | 1 | | 0 | | | 1 | | | 0 | | 1 | | |  | | |  | |  | |  | |  | | 5/7 | | | |
| Wiberg et al. (1999) | 1 | 1 | | 0 | 1 | | | | | 1 | 1 | | | 0 | | | |  | |  | | |  | | |  | | |  | |  | |  | | |  | | |  | |  | | |  | | |  | |  | |  | |  | | 5/7 | | | |
| Zhang and Synder (2004) | 1 | 1 | |  |  | | | | |  |  | | |  | | | |  | |  | | |  | | |  | | |  | | 1 | | 0 | | | 1 | | | 0 | | 1 | | |  | | |  | |  | |  | |  | | 5/7 | | | |
| Scoring: 0 – ‘no’ or ‘can’t tell’, 1 – ‘yes’ | | | | | | | | | | | | | | | | | | | | | | | | | | | | | | | | | | | | | | | | | | | | | | | | | | | | | | | | | | |
| **Critical Appraisal Consensus Scores for Grey Literature - Guidelines** | | | | | | | | | | | | | | | | | | | | | | | | | | | | | | | | | | | | | | | | | | | | | | | | | | | | | | | | | | |
| **Title and Authors (Year)** | | | | | | | **iCAHE Guideline Quality Check List** | | | | | | | | | | | | | | | | | | | | | | | | | | | | | | | | | | | | | | | | | | |  |  |  |  |  |  |  |  |  |
|  | | | | | | | Full text | | Reference List | | | Summary of recommendations | | | Date of completion | | | | Anticipated review date | | Dates of inclusion | | | Strategy | | | | Levels of evidence | | Appraisal of recommendations | | | | Hierarchy of recommendations | | | Developers | | | Qualifications | | Purpose and target users | | | | Readability | | Critical Appraisal Scores | |  |  |  |  |  |  |  |  |  |
| Clinical Practice Guideline – Vertebral Subluxation in Chiropractic Practice (3^rd^ Edition). Council of Chiropractic Practice (2008) | | | | | | | 1 | | 1 | | | 1 | | | 1 | | | | 1 | | 1 | | | 1 | | | | 1 | | 0 | | | | 1 | | | 1 | | | 1 | | 1 | | | | 1 | | 13 | |  |  |  |  |  |  |  |  |  |
| Infants and Children: Acute Management of the Unsettled and Crying Infant (1^st^ Edition).  NSW Government (2016) | | | | | | | 1 | | 1 | | | 1 | | | 1 | | | | 1 | | 0 | | | 0 | | | | 0 | | 0 | | | | 0 | | | 1 | | | 1 | | 1 | | | | 1 | | 9 | |  |  |  |  |  |  |  |  |  |
| Scoring: 0 – ‘no’, 1 – ‘yes’ | | | | | | | | | | | | | | | | | | | | | | | | | | | | | | | | | | | | | | | | | | | | | | | | | |  |  |  |  |  |  |  |  |  |
| **Critical Appraisal Consensus Scores for Grey Literature - Text and Opinion** | | | | | | | | | | | | | | | | | | | | | | | | | | | | | | | | | | | | | | | | | | | | | | | | | | | | | | | | | | |
| **Authors (Year)** | | | | | | **JBI Critical Appraisal Checklist for Text and Opinion Papers** | | | | | | | | | | | | | | | | | | | | | | | | | | | | | | | | | | | | | | | | | | | | | | | | | | | | |
|  | | | | | | Source of opinion | | | | | | | | | | Standing in field | | | | | | | | | Population | | | | | | | | | | Analytical Process | | | | | | | | | | Extant literature | | | | | | | Incongruence with literature | | | | Critical Appraisal Score | | |
| World Federation of Chiropractic (WFC) (2019) | | | | | | 0 | | | | | | | | | | 0 | | | | | | | | | 1 | | | | | | | | | | 0 | | | | | | | | | | 1 | | | | | | | 0 | | | | 2 | | |
| Chiropractic Board of Australia (CBA) (2017) | | | | | | 0 | | | | | | | | | | 0 | | | | | | | | | 1 | | | | | | | | | | 1 | | | | | | | | | | 1 | | | | | | | 1 | | | | 4 | | |
| Chiropractic Association of Australia (CAA) (2016) | | | | | | 0 | | | | | | | | | | 0 | | | | | | | | | 1 | | | | | | | | | | 1 | | | | | | | | | | 1 | | | | | | | 1 | | | | 4 | | |
| International Chiropractic Association (ICA) (2019) | | | | | | 1 | | | | | | | | | | 1 | | | | | | | | | 1 | | | | | | | | | | 1 | | | | | | | | | | 1 | | | | | | | 1 | | | | 6 | | |
| Jennifer Barham-Floreani (2014) | | | | | | 1 | | | | | | | | | | 1 | | | | | | | | | 1 | | | | | | | | | | 0 | | | | | | | | | | 1 | | | | | | | 0 | | | | 4 | | |
| Loretta Marron (2011) | | | | | | 1 | | | | | | | | | | 1 | | | | | | | | | 1 | | | | | | | | | | 1 | | | | | | | | | | 1 | | | | | | | 1 | | | | 6 | | |
| Pat Chevrier (2016) | | | | | | 1 | | | | | | | | | | 1 | | | | | | | | | 1 | | | | | | | | | | 1 | | | | | | | | | | 1 | | | | | | | 1 | | | | 6 | | |
| Sharon Kirkey (May 2019)  In College of Chiropractors of Ontario (2019) | | | | | | 1 | | | | | | | | | | U | | | | | | | | | 1 | | | | | | | | | | 0 | | | | | | | | | | 0 | | | | | | | 1 | | | | 3 | | |
| Sharon Kirkey (July 2019)  In College of Chiropractors of Ontario (2019) | | | | | | 1 | | | | | | | | | | U | | | | | | | | | 1 | | | | | | | | | | 0 | | | | | | | | | | 0 | | | | | | | 0 | | | | 2 | | |
| Meghan Collie (2019)  In College of Chiropractors of Ontario (2019) | | | | | | 1 | | | | | | | | | | 0 | | | | | | | | | U | | | | | | | | | | 0 | | | | | | | | | | 1 | | | | | | | 0 | | | | 2 | | |
| Lindsay (2019)  In College of Chiropractors of Ontario (2019) | | | | | | 1 | | | | | | | | | | 0 | | | | | | | | | 1 | | | | | | | | | | 0 | | | | | | | | | | 1 | | | | | | | 0 | | | | 3 | | |
| Rosner (2003) | | | | | | 1 | | | | | | | | | | 1 | | | | | | | | | 1 | | | | | | | | | | 1 | | | | | | | | | | 1 | | | | | | | 0 | | | | 5 | | |
| Australian Chiropractic Association (ACA) (2019) | | | | | | 0 | | | | | | | | | | 0 | | | | | | | | | 1 | | | | | | | | | | 1 | | | | | | | | | | 1 | | | | | | | 1 | | | | 4 | | |
| Mitchell Sellhorst (2015) | | | | | | 1 | | | | | | | | | | 1 | | | | | | | | | 1 | | | | | | | | | | 1 | | | | | | | | | | 1 | | | | | | | 1 | | | | 5 | | |
| Scoring: 0 – ‘no’, 1 – ‘yes’ | | | | | | | | | | | | | | | | | | | | | | | | | | | | | | | | | | | | | | | | | | | | | | | | | | | | | | | | | | |
