# Supplementary material for: Spinal manipulation and mobilisation in the treatment of infants, children, and adolescents: a systematic scoping review
Source: BMC Pediatr. 2022 Dec 19;22:721. doi: 10.1186/s12887-022-03781-6 (PMC9762100; doi:10.1186/s12887-022-03781-6)
Supplement: Supplementary file 3 — Additional file 3: Supplementary File 3. Data Extraction [file 12887_2022_3781_MOESM3_ESM.docx]

| **SUPPLEMENTARY FILE 3** Data Extraction | | | | | | | | | | | | |  |
| --- | --- | --- | --- | --- | --- | --- | --- | --- | --- | --- | --- | --- | --- |
| **Data Extraction for Reviews** | | | | | | | | | | | | |  |
| Review | | **Aim/Purpose/Context** | | **Participants** | | | **Databases searched [Date range]** | | **Critical Appraisal Tools used** | | | **Main Findings/Adverse Events** |  |
| Alcantara et al (2011a)  ASD | | **Aim/Purpose:** To examine to what degree claims are supported by the scientific literature.  **Professional Context:** Chiropractic | | n= 5 articles: 3 case studies, 1 cohort study and 1 RCT  **Inclusion Criteria:**  1. Primary investigation/ report published in an English peer-reviewed journal 2. Patients <18 years 3. Patients are diagnosed with autism, Asperger’s Syndrome, PDD-NOS, or ASD | | | **No. of databases**: 8  **Databases and date ranges:** MANTIS [1965-2010]; ICL [1984- 2010]; PubMed [1966-2010]; EMBASE [1974-2010], AMED[1975-2010], CINAHL [1965-2010], Alt-Health Watch [1965- 2010], and PsychINFO [1965-2010].  Additionally, chiropractic journals (i.e., Journal of Manipulative and Physiological Therapeutics, Journal of the Canadian Chiropractic Association, Clinical Chiropractic, The Chiropractic Journal of Australia, and the Journal of Clinical Chiropractic Paediatrics) were hand searched from 2006-2011 for possible relevant materials. | | Nil critical appraisal conducted  **Outcomes:**  Aguilar et al: - Modified Autism Checklist and Childhood Autism Rating Scale -Brain stem-evoked potential recordings, pre-treatment and comparative radiographic examination, a dual probe infrared heat recording of paraspinal soft tissue structures, and supine leg check analysis - Decreased medication use (i.e., Ritalin), and improved function (i.e., improved bowel movement and bowel control, decreased aggression, and hyperactivity Korshid et al: - Autism Treatment Evaluation Checklist (ATEC) | | | **Main Findings:** A total of five articles consisting of three case reports, one cohort study and one RCT were found suggesting literature exploring chiropractic care of children with ASD is lacking. Literature base is lacking, both in terms of quality and quantity. This systematic review demonstrates a need for more research to examine the safety and effectiveness of chiropractic SMT and adjunctive therapies as they relate to autism.  **Adverse events**: Adverse events associated with chiropractic SMT in children were found to be rare. Acknowledge adverse events may be underreported, but in terms of what is documented thus far, adverse events associated with chiropractic SMT are mild, self-limiting, and do not require medical attention or hospitalisation. |  |
| Alcantara et al (2011b)  Infantile Colic | | **Aim/ Purpose:** To perform a systematic review of the literature on the chiropractic care of patients with infantile colic.  **Professional Context:** Chiropractic | | n= 26 articles- 3 clinical trials, 2 survey studies, 6 case reports, 2 case series, 4 cohort studies, 5 commentaries, and 4 reviews of literature Population: Infants  **Inclusion Criteria:** (1) The manuscript was of a primary investigation/report (i.e., case reports, case series, case control, randomised controlled trials (RCTs), and survey or surveillance studies) published in a peer-reviewed journal in the English language (2) Part or all of the study population involved patients 18 years or younger (3) The topic involved the chiropractic care of a patient with colic. | | | **No. of databases**: 8   **Databases and date ranges**: MANTIS [1965-2010], PubMed [1966- 2010], Index to Chiropractic Literature [1984-2010], EMBASE [1974-2010], AMED [1967-2010], CINAHL [1964-2010], Alt- Health Watch [1965-2010], and PsychINFO [1965-2010]. Additionally, chiropractic journals (i.e., Journal of Manipulative and Physiological Therapeutics, Journal of the Canadian Chiropractic Association, Clinical Chiropractic, The Chiropractic Journal of Australia, and the Journal of Clinical Chiropractic Paediatrics) were hand searched from 2006-2011. | | Nil critical appraisal was conducted  **Outcomes**: Crying time | | | **Main Findings:** Chiropractic offers a safe and effective alternative treatment approach for the child with infantile colic. In the context of safety and effectiveness of chiropractic SMT for infantile colic, a trial of chiropractic care is warranted and congruent with the needs and wants of parents for their colicky infant and in keeping with biomedical ethics.  **Adverse Events**: Chiropractic SMT and children is essentially safe. |  |
| Alcantara et al (2015)  Breastfeeding Difficulties | | **Aim/ Purpose:** Discuss the clinical rationale of chiropractic care for infants with breastfeeding difficulties to help inform clinical practice.  **Professional Context:** Chiropractic | | n= 24 articles - 8 case reports, 2 case series, 3 cohort studies, 6 manuscripts (5 case reports and a case series)  **Inclusion Criteria:** (1) Reports that addressed or described the chiropractic care of infants with breastfeeding difficulties regardless of peer-review.  Participants: Infants 0-6 months | | | **No. of databases**: 3  **Databases and date range**: PubMed [1966–2013], Manual, Alternative and Natural Therapy Index System (MANTIS) [1964–2013] and Index to Chiropractic Literature [1984–2013]. | | Nil critical appraisal was conducted  **Outcomes:** Not reported. | | | **Main Findings**: Theoretical and clinical rational for addressing infants with breastfeeding difficulties by correcting cervico-cranio-mandibular dysfunction, utilizing the full spectrum of chiropractic care including spinal and extra-spinal manipulation as well as adjunctive therapies.  **Adverse Events**: Not reported. |  |
|  | | **Aim/Purpose/Context** | | **Participants** | | | **Databases searched/Date range of database** | | **Critical Appraisal Tools used** | | | **Main Findings/Adverse Events** |  |
| Brand et al (2005)  Infantile Colic | | **Aim/ Purpose:**  To establish the effects of manual therapy, chiropractic, or osteopathic treatment of the kinetic imbalance due to suboccipital strain (KISS) syndrome in infants with positional preference, plagiocephaly, and colic.  **Professional Context:** Chiropractic and Osteopathic | | n= 2 RCT's (about infantile colic, not KISS as not studies found on KISS).   **Inclusion Criteria:** (1) Randomised controlled trials. (2) Available electronic databases  Participants: Infants 0-23 months | | | **No. of databases**: 3  **Databases:** PubMed (Medline), Embase and Cochrane  **Date range**: 1966-2004 | | Nil critical appraisal was conducted  **Outcomes**: Crying time (as no studies found on KISS). | | | **Main Findings:**  - No effect of chiropractic treatment on crying behaviour after 8 weeks was found. - No evidence supporting spinal manipulation by manual physical therapists, chiropractors, or osteopaths, in infants with signs and symptoms proposed to be indicative of the KISS syndrome. - No scientific evidence that spinal manipulation by manual physical therapists, osteopaths, or chiropractors is useful in infants with signs and symptoms of the proposed KISS syndrome.  **Adverse Events**: Vegetative reactions, bradycardia, tachycardia, and reflex apnoea recorded in more than half of patients. Although apnoea was of short duration (<10 sec) and reversible, it can be regarded as a potentially life-threatening adverse event. |  |
| Bronfort et al (2010)  Multiple Conditions | | **Aim/Purpose:** To provide a brief and succinct summary of the scientific evidence regarding the effectiveness of manual treatment as a therapeutic option for the management of a variety of musculoskeletal and non-musculoskeletal conditions based on the volume and quality of the evidence.  **Professional Context:** Chiropractic and Osteopathic | | Paediatric related reviews:  Infantile Colic: 2 systematic reviews, 8 RCT's  Nocturnal Enuresis: 2 systematic reviews, 2 RCT's  **Inclusion Criteria**: Not clearly reported. | | | **No. of databases:** 9  **Databases:** MEDLINE (PubMed), Ovid, Mantis, Index to Chiropractic Literature, CINAHL, the specialized databases Cochrane Airways Group trial registry, Cochrane Complementary Medicine Field, and Cochrane Rehabilitation Field  **Date range:** NR | | The Cochrane Collaboration tool   **Outcomes:**  - Asthma: Lung function and symptom severity. - Infantile Colic: Crying time. - Nocturnal Enuresis: Not reported. - Otitis Media: Not reported. | | | **Main Findings:**  Infantile Colic - Inconclusive evidence in an unclear direction regarding the effectiveness of osteopathic manipulative therapy for otitis media. Moderate quality evidence that spinal manipulation is no more effective than sham spinal manipulation for the treatment of infantile colic (i.e., spinal manipulation is not effective for managing infantile colic).  Nocturnal Enuresis- Inconclusive evidence in a favourable direction regarding the effectiveness of chiropractic care for the treatment of enuresis.  Otitis Media- Inconclusive evidence in an unclear direction regarding the effectiveness of osteopathic manipulative therapy for otitis media.  Health professionals should be permitted to use generally safe but as yet unproven methods given that professionals operate within the context of EBH. For children, the evidence is inconclusive regarding the effectiveness of spinal manipulation/ mobilization for otitis media and enuresis, but shows it is not effective for infantile colic and for improving lung function in asthma when compared to sham manipulation.  **Adverse Events**: True incidence of serious adverse events in children as a result of spinal manipulation remains unknown. |  |
|  | | **Aim/Purpose/Context** | | **Participants** | | | **Databases searched/Date range of database** | | **Critical Appraisal Tools used** | | | **Main Findings/Adverse Events** |  |
| Brurberg et al (2019)  Infant Torticollis | | **Aim/Purpose:** Update of a systematic review published by the Norwegian Knowledge Centre for the Health Services in 2009. In addition to using the search results from the earlier review, we also performed a search covering the period from the original search to August 2017  **Professional Context:** Chiropractic, physiotherapy, manual therapy and osteopathy | | n= 6 publications: 3 RCTS, 2 of which examined the efficacy of manual therapy and 1, osteopathy   **Inclusion Criteria for Efficacy**: (1) Randomised, quasi-randomised and non-randomised controlled studies (2) Infants (0–2 years) in whom cervical joint- induced symmetry disorder was suspected (3) Manual therapy, chiropractic, osteopathy and physiotherapy targeting the cervical spine (4) No treatment, or placebo or other forms of treatment not targeting the cervical joints (5) Degree of symmetry, sleep disorders, duration of crying, passive rotation, etc.  **Inclusion Criteria for Adverse Effects**: (1) All, including case reports (2) Infants (0–2 years) treated for cervical joint-induced symmetry disorder. (3) Manual therapy, chiropractic, osteopathy and physiotherapy targeting the cervical spine (4) Respiratory problems, nerve damage, paralysis, death, etc. | | | **No. of databases:** 7  **Databases:** MEDLINE, EMBASE, CINAHL, Cochrane CENTRAL, AMED, PEDro, and the Index to Chiropractic Literature  **Date Range:** Up to August 2017 | | The Cochrane Collaboration’s tool, GRADE assessment  **Outcomes:**  Efficacy: Degree of symmetry (Alberta Infant Motor Scale and Argenta scale), sleep disorders, duration of crying, passive cervical flexion and rotation  **Safety**: Respiratory problems, nerve damage, paralysis, death | | | **Main Findings**: Research did not support the use of manipulation techniques in the treatment of infantile asymmetry, but there was uncertainty associated with the research findings. Since the current evidence base consists of a small number of studies with few participants, it is difficult to draw firm conclusions, but our findings do not support the use of manipulation techniques in the treatment of infantile asymmetry.  **Adverse Events:** The randomised studies included in this review did not report adverse effects. |  |
| Carnes et al (2018)  Infantile Colic / Excessive Crying | | **Aim/Purpose:** To update the Cochrane review of RCTs for crying time and investigate non-RCT studies and outcomes that are important to parents, rather than biomedical markers alone that might be of more interest to primary researchers exploring aetiology as our selected population was infants that were considered healthy.  **Professional Context:** Physiotherapy, Chiropractic, Osteopathy | | n= 19 articles: 7 RCT, 7 case series, 3 cohorts, 1 service evaluation, 1 qualitative  **Inclusion Criteria:** (1) RCTs, prospective cohort studies, observational studies, case control studies, case series, questionnaire surveys and qualitative studies. Participants**:** Infants 0-12 months | | | **No. of databases:** 9  **Databases:**  Medline Ovid, Embase, Web of Science (WOS), Physiotherapy Evidence Database, Osteopathic Medicine Digital Repository, Cochrane (all databases), Index of Chiropractic Literature, Open Access Theses and Dissertations and Cumulative Index to Nursing and Allied Health Literature  **Date range:** January 1990 to January 2017 | | The Cochrane Collaboration Tool  **Outcomes**: Excessive crying, lack of sleep, displays of distress or discomfort (back arching and drawing up of legs) and difficulty feeding. | | | **Main Findings:** Significant outcome of manual therapy for reduced crying time of 1.27 (95% CI −2.19 to −0.36) hours per day. Overall RR of 0.12 (95% CI 0.12 to 0.66); that is, those who had manual therapy had an 88% reduced risk of having an adverse event compared with those who did not have manual therapy. Moderate favourable evidence for the reduction in crying time in infants receiving manual therapy care (around 1 hour per day). Do not know if this result is meaningful to parents or if the reduction is due to the manual therapy component of care or other aspects of care. For other outcomes, the strength of evidence was low and inconclusive.  **Adverse Events**: 1308 infants exposed to manual therapy and nine non-serious adverse events recorded, giving an incidence rate of seven non-serious events per 1000 infants. Conversely, there were 11 non-serious adverse events in the infants not exposed to manual therapy (n=97), giving an incidence rate of around 110 per 1000 infants. The safety data we extracted regarding adverse events indicated that manual therapy is a relatively low risk intervention, reflecting similar findings in other studies. Relative Risk (RR): 0.12 (95% CI of 0.12 to 0.66). Those who had manual therapy had an 88% reduced risk of having an adverse event compared with those who did not have manual therapy. (These results only included the 4 RCTS in the review). |  |
|  | | **Aim/Purpose/Context** | | **Participants** | | | **Databases searched/Date range of database** | | **Critical Appraisal Tools used** | | | **Main Findings/Adverse Events** |  |
| Clar et al (2014)  Multiple Conditions | | **Aim/Purpose:** (1) Updated and extended the “UK evidence report” by Bronfort et al.  (2) Synthesise evidence additional to Bronfort et al.  (3) Compare conclusions from the additional studies summarised to those of Bronfort et al. focusing in particular on areas where it was stated that the available evidence was inconclusive or that manual therapy was not effective report.  **Professional Context:** Chiropractic and Osteopathic | | n=178 articles: 72 systematic reviews, 96 RCTs, 10 non-randomised primary studies  **Inclusion criteria:** (1) Full text reports of systematic reviews, RCTs or controlled clinical trials (CCTs), cohort studies with a comparison group, or qualitative studies of patients' views on manual therapy. (2) Primary studies to include at least 20 participants. (3) Studies had to include participants of any age and in any setting treated for any musculoskeletal or on musculoskeletal condition who were treated with any manual treatment/ therapy were included (alone or in combination). (3) Interventions to include an element of manipulation or mobilisation, and emphasis was on interventions typically carried out by a manual therapist/ chiropractor/ osteopath. | | | **No. of databases:** 17  **Databases:** MEDLINE (Ovid), EMBASE, Mantis, Index to Chiropractic Literature, CINAHL, the specialised databases Cochrane Airways Group trial register, Cochrane Complementary Medicine Field register, and Cochrane Rehabilitation Field register (via CENTRAL). We supplemented these searches by using the following other databases: Science Citation Index, AMED, CDSR, NHS DARE, NHS HTA, NHS EED, CENTRAL (full search), and ASSIA, Social Science Citation Index.  **Date range:** NR | | AMSTAR (for systematic reviews); Cochrane Risk of Bias (for RCTs); CRD checklist (for controlled cohort studies); and CASP (for qualitative studies)  **Outcomes:**  - ADHD: Not reported. - CP: leg or hand use and in sleep documented by parents, drooling. Gross Motor Function Measurement percent and Functional Independence Measure for Children mobility - Paediatric dysfunctional voiding: vesicoureteral reflux and/daytime incontinence - Infantile Colic: Crying time - Otitis Media: Not reported. | | | **Main Findings:**  - Cerebral Palsy: Inconclusive (unclear) evidence for the effectiveness of osteopathic manual therapy in the treatment of cerebral palsy (not evaluated in the UK evidence report). - Paediatric Dysfunctional Voiding: Inconclusive (favourable) evidence for osteopathic manual therapy improving symptoms of paediatric dysfunctional voiding (not evaluated in the UK evidence report). - Otitis Media: Inconclusive (unclear) evidence for osteopathic manual therapy in treating otitis media (no change from the UK evidence report). - Paediatric Nocturnal Enuresis: Inconclusive (favourable) evidence for spinal manipulation in paediatric nocturnal enuresis (no change from the UK evidence report). - ADHD: Given the severe methodological limitations of the included studies, there is inconclusive (unclear) evidence regarding the effectiveness of osteopathic treatment for ADHD - Infantile Colic: Inconclusive (favourable) evidence for cranial osteopathic manual therapy in treating infantile colic.  **Adverse Events:** - Asthma: Only one study reported on adverse events (non-reported). - Poorly and scarcely reported harms data limited our ability to make meaningful comparisons of rates of adverse events between the treatments. |  |
| Corso et al (2020)  Multiple Conditions | | **Aim/ Purpose:** (1) Describe adverse events (2) Report the incidence of adverse events (3) Determine whether SMT increases the risk of adverse events compared to other interventions.  **Professional Context:** Chiropractic | | n= 3: 2 RCT, 1 cohort  **Inclusion Criteria:**  (1) Case report, case series, case-control study, cohort study or RCT Participants: 0-9 years | | | **No. of databases**: 3  **Databases:** MEDLINE, CINAHL, and Index to Chiropractic Literature   **Date range:** January 1, 1990 to August 1, 2019 | | Scottish Intercollegiate Guidelines Network (SIGN)   **Outcome**: Daily Crying Time | | | **Main Findings:** Most studies report mild and transient adverse events in children under 10 years receiving SMT. The risk of moderate and severe adverse events is unknown in children treated with SMT. It is unclear whether SMT increases the risk of adverse events in children < 10 years old.  **Adverse Events/ Major Findings**: Most adverse events are mild (e.g., increased crying, soreness). One case report describes a severe adverse event (rib fracture in a 21-day-old) and another an indirect harm in a 4-month-old. The incidence of mild adverse events ranges from 0.3% (95% CI: 0.06, 1.82) to 22.22% (95% CI: 6.32, 54.74). This review suggests that the risk of mild adverse events ranges from 0.3% (95% CI: 0.06, 1.82) in infants < 27 weeks old treated with mild mobilisation of C1 for upper cervical dysfunction to 22.22% (95% CI: 6.32, 54.74) in children aged between 6 months and 6 years treated for otitis media.  Relative Risk (RR): 2.44 (0.26, 22.8). Incidence of AE in treatment group: 22.22% (6.32, 54.74) One parent of a child in placebo group reported excessive crying after treatment. Incidence of AE in the placebo group: 9.09% (1.62, 37.74) One parent in treatment group reported child had mid-back soreness after 1 treatment which resolved after a few days. Another child was reported to be irritable for a short time after treatment. |  |
|  | | **Aim/Purpose/Context** | | **Participants** | | | **Databases searched/Date range of database** | | **Critical Appraisal Tools used** | | | **Main Findings/Adverse Events** |  |
| Dobson et al (2012)  Infantile Colic | | **Aim/Purpose:** To evaluate the results of studies designed to address efficacy or effectiveness of manipulative therapies (specifically, chiropractic, osteopathy and cranial manipulation) for infantile colic in infants less than six months of age.  **Professional Context:** Chiropractic and Osteopathic | | n= 6 studies  **Inclusion Criteria:**  (1) Randomised trials evaluating the effect of chiropractic, osteopathy or cranial osteopathy alone or in conjunction with other interventions for the treatment of infantile colic. Participants: Infants <6 months | | | **No. of databases**: 9  **Databases and date ranges:** CENTRAL (2012, Issue 4), MEDLINE (1948 to April Week 3 2012), EMBASE (1980 to 2012 Week 17), CINAHL (1938 to April 2012), PsycINFO (1806 to April 2012), Science Citation Index (1970 to April 2012), Social Science Citation Index (1970 to April 2012), Conference Proceedings Citation Index - Science (1990 to April 2012) and Conference Proceedings Citation Index - Social Science & Humanities (1970 to April 2012). | | Cochrane Handbook for Systematic Reviews of Interventions and GRADE assessment  **Outcomes:**  Primary Outcomes: Daily hours of crying, daily hours of sleeping, presence/ Absence of Colic  **Secondary Outcomes:**  1. Changes in frequency of crying bouts (number of crying episodes per day) (post-treatment versus baseline). 2. Measures of parental or family quality of life. 3. Measures of parental stress, anxiety or depression. 4. Sleeping time, that is, change in duration of peaceful sleeping (post-treatment versus baseline). 5. Parental satisfaction. | | | **Main Findings**: Five studies measured daily hours of crying and these data were combined, suggesting that manipulative therapies had a significant effect on infant colic reducing average crying time by one hour and 12 minutes per day (mean difference (MD) -1.20; 95% confidence interval (CI) -1.89 to -0.51), however when only studies with parental blinding were included, these improvements were not significant (MD – 0.57; 95% CI -2.24 to 1.09). One study measured infant sleeping time and found manipulative therapy resulted in statistically significant improvement (MD 1.17; 95% CI 0.22 to 2.12). Most included trials appeared to indicate that the parents of infants receiving manipulative therapies reported fewer hours crying per day than parents whose infants did not, based on contemporaneous crying diaries, and this difference was statistically significant. Analysis of data from three studies indicated that manipulative therapies did not result in significantly higher proportions of parents reporting recovery from colic (OR 11.12; 95% CI 0.46 to 267.52).  **Adverse Events**: One of the studies recorded adverse events and none were encountered. |  |
| Driehuis et al (2019)  Multiple Conditions | | **Aim/Purpose:** To conduct a systematic review of the evidence for effectiveness and harms of specific SMT techniques for infants, children and adolescents. This systematic review and meta-analysis of the literature provides a broad overview of the evidence regarding the effectiveness and harms of specific SMT techniques in infants, children and adolescents, related to specified treatment indication.  **Professional Context:** Chiropractic, Physiotherapy and Osteopathic | | n=26 articles; 12 controlled trials, of which 10 were randomised controlled trials, 9 observational studies, and 5 case reports  **Inclusion Criteria:** (1) Controlled studies were included to investigate effectiveness and harms.  (2) Observational studies and case reports were included to investigate harms Participants: 0-18 years | | | **No. of databases:** 5  **Databases:** PubMed, Index to Chiropractic Literature, Embase, CINAHL and Cochrane Library.   **Date range**: Up to 20 December 2017 | | Cochrane Risk of Bias tool. Observational studies were assessed with the Item Bank for Assessing Risk of Bias and Confounding for Observational Studies of Interventions or Exposures. Risk of bias of case reports was assessed using the JBI Critical Appraisal Checklist for Case Reports.  **Outcomes:** Crying Hours, Changes in Torticollis, Peakflow (FEV1), symptoms, medication use, quality of life, headache duration, VAS, frequency of bed wetting. | | | **Main Findings:** Two studies compared SMT to no treatment. Miller et al. compared a blinded treatment group (n = 35), non-blinded treatment group (n = 33) and a non-treatment group (n = 34) and found that crying hours significantly decreased (p<0.05) with 1.5 hours/day after 10 days between blinded treatment and non-treatment.  Gentle, low-velocity spinal mobilisations seem to be a safe treatment technique. Although scarcely reported, HVLA manipulations in infants and young children could lead to severe harms. Severe harms were likely to be associated with unexamined or missed underlying medical pathology. Due to very low-quality evidence,   - uncertain whether SMT consisting of HVLA manipulations improves lung function in children/adolescents with asthma. - uncertainty about the effect of SMT consisting of upper cervical manipulations on reducing autism related symptoms in children/adolescents with autism. - uncertain about the effect of cervical SMT with HVLA manipulations on reducing headache related symptoms in children/ adolescents with headache - uncertain whether SMT consisting of HVLA manipulations reduces the frequency of bed-wetting in children with nocturnal enuresis. - uncertainty about the effect of segmental spinal mobilizations on reducing back pain and increasing quality of life in adolescents with idiopathic scoliosis.   **Adverse Events:** Nine observational studies, five case reports, and four controlled studies reported on harms. Two controlled studies reported mild, transient harms in terms of side effects. Five studies reported harms after HVLA manipulations performed on the full spine. In three of these studies a small number of mild harms was reported; the other two studies (n = 145) reported no harms. No studies were found reporting on harms after cervical or full spine mobilizations. One study (n = 956) reported side effects or reactions in children after chiropractic treatment (n = 557), but both side effects or reactions and treatment techniques were not specified. Hence, conclusions on treatment technique cannot be given. |  |
|  | | **Aim/Purpose/Context** | | **Participants** | | | **Databases searched/Date range of database** | | **Critical Appraisal Tools used** | | | **Main Findings/Adverse Events** |  |
| Edwards & Miller (2019)  Breastfeeding Difficulties | | **Aim/Purpose:** To find and evaluate the types of clinical studies that have been done in the arena of chiropractic care for suboptimal infant breastfeeding, to determine whether a RCT was an appropriate next step.  **Professional Context**: Chiropractic | | n=12 studies  **Inclusion Criteria:**  (1) Primary studies that addressed or described chiropractic or chiropractic/ multidisciplinary care of breastfed, infant, human participants were included.  (2) ll study designs were included with no restriction in terms of publication or date. (3) Only articles published in the English language were included. | | | **No. of databases**: 4  **Databases:** PubMed, Cumulative Index to Nursing and Allied Health Literature (CINAHL), Index to Chiropractic Literature (ICL) and the Cochrane Library were searched  **Date range:** NR | | Nil critical appraisal was conducted  **Outcomes:** Improved breastfeeding, exclusive breastfeeding, extended breastfeeding. | | | **Main Findings**: Both chiropractic care alone and in a multi-disciplinary setting appear to help sub-optimal breastfeeding but are supported by the lowest levels of scientific evidence and, as such, no statistical significance can be drawn from the positive results reported.  **Adverse Events**: No independent conclusion made. |  |
| Ellwood et al (2020)  Plagiocephaly & Torticollis | | **Aim/Purpose:** To investigate for congenital muscular torticollis (CMT) and positional plagiocephaly (PP) the effectiveness and safety of manual therapy, repositioning and helmet therapy (PP only) using a systematic review of systematic reviews and national guidelines.  **Professional Context**: Chiropractic | | n= 10 articles  **Inclusion Criteria:** (1) We included reviews that reported a systematic review methodology with more than one reviewer indicated in the review process. (2) We included guidelines where clear methodological procedures for development were reported. (3) Only systematic reviews and national guidelines published in English within the last 20 years. | | | **No. of databases**: 4  **Databases**: PubMed, MANTIS, Embase, and Cochrane databases  **Date range:**1999–2019 | | AMSTAR 2  **Outcomes**: Cranial asymmetry and/or cervical range of movement. | | | **Main Findings**: The results for CMT showed that manual therapy had moderate favourable evidence for increasing range of movement and there was low quality inconclusive evidence to support SMT in addition to physiotherapy. Manual therapy for PP showed favourable outcomes when compared with repositioning therapy but equivocal low-level evidence when compared with helmet therapy.  **Adverse Events**: No adverse serious events were reported in any of the RCTs reviewed. 2 studies looked at adverse events in other types of studies and case report studies. Both found examples of serious adverse events. One case reported by Driehuis et al. (2019) described temporary quadriplegia following treatment in 4-monthold boy. The other case was reported in 2 studies which described death in a 3-month-old girl. |  |
| Ernst, E. (2009)  Infantile Colic | | **Aim/ Purpose:** Critically evaluate the data from randomised clinical trials of chiropractic spinal manipulation as a treatment of infant colic.  **Professional Context:** Chiropractic | | n= 3 RCTS  **Inclusion Criteria:**  (1) randomised, test the effectiveness of spinal manipulation, focus on infants with the clinical diagnosis of colic and include outcome measures which are of clinical importance. Participants: 0-10 weeks | | | **No. of databases**: 4  **Databases:** Amed, Embase, Medline (using the Ovid interface) and Cinahl (using the Ebsco interface)  **Date range**: Up to January 2009 | | Nil critical appraisal was conducted  **Outcomes:** Hours of crying time daily. | | | **Main Findings**: RCT's fail to demonstrate that chiropractic spinal manipulation is an effective therapy for infant colic. In conclusion, the current evidence from RCTs does not show that chiropractic spinal manipulation is an effective treatment for infant colic.  **Adverse Events**: Not reported. |  |
| Fairest et al (2019)  ADHD | | **Aim/Purpose**: To report the improvements in symptoms associated with ADHD in a 7-year-old female following chiropractic care and to narratively review previous literature.  **Professional Context:** Chiropractic | | Narrative review of n= 3 systematic reviews, 1 clinical trial, 1 qualitative study, 4 case series and 18 case reports  **Inclusion Criteria:**  (1) Peer-reviewed and complete systematic reviews, clinical trials, case series and case reports | | | **No. of databases**: 3  **Databases:** The Index to Chiropractic Literature, PubMed and Google Scholar  **Date range**: Up to July 2017 | | Nil critical appraisal was conducted  **Outcomes:** Sleeping pattern and anxiety. | | | **Main Findings**: Current literature suggests that chiropractic care can improve symptoms related to ADHD. Chiropractic care, using instrument assisted Modified Diversified technique for the correction of vertebral subluxation, was associated with improvements in the child’s presenting symptoms associated with ADHD. This study supports the use of chiropractic care for children and young adults with ADHD.  **Adverse Events**: Not reported. |  |
|  | | **Aim/Purpose/Context** | | **Participants** | | | **Databases searched/Date range of database** | | **Critical Appraisal Tools used** | | | **Main Findings/Adverse Events** |  |
| Ferrance & Miller (2010)  Multiple Conditions | | **Aim/Purpose:** To summarise the literature from the point of view of clinicians, rather than researchers, and to discuss some additional detail of the conditions themselves.  **Professional Context**: Chiropractic | | **Inclusion Criteria**: NR | | | **No. of databases**: 4  **Databases**: PubMed, Mantis, Index to Chiropractic Literature, and CINAHL  **Date range**: NR | | Nil critical appraisal was conducted  **Outcomes**:  - Enuresis: Crying time, sleep time and maternal stress - Other paediatric conditions: Not reported. | | | **Main Findings**: For the crying infant, there is some (contradictory) evidence to suggest that chiropractors may have a positive influence on this distressing problem of infancy. For enuresis, the chiropractor (if called upon) as well as the medical physician can demystify the problem and offer suggestions on behaviour modification and alarms and, when appropriate, evaluate for more significant physical disorders. In asthma, as studies have shown, a positive impact on quality of life has been observed and documented in several different studies but the evidence is otherwise negative for chiropractic. Notwithstanding this, the conscientious and educated chiropractor, while working within his or her scope of practice, can potentially be a valuable member of the paediatric health care team."  **Adverse Events:** Not reported. |  |
| Fry, L (2014)  Breastfeeding Difficulties | | **Aim/purpose:** To investigate the available evidence to support the role chiropractic may play in treating breastfeeding dysfunction.  **Professional Context**: Chiropractic | | 11 articles were reviewed inclusive of: 6 case studies, 3 case series, 1 clinical trial and 1 narrative  **Inclusion Criteria**:  (1) Only articles published in the English language in a peer reviewed journal (2) Infant human participants  (3) Chiropractic treatment for breastfeeding dysfunction | | | **No. of databases**: 5  **Databases:** PubMed, MEDLINE (ProQuest), CINAHL and Index to Chiropractic Literature (ICL)  **Date range**: from inception through to December 2013 | | Nil critical appraisal was conducted  **Outcomes:** biomechanical changes to the upper cx spine (atlas or atlantooccipital joint) for breastfeeding complications and improvement in breast feeding (ability to latch, suck etc.). | | | **Main Findings:** Limited evidence exists to support chiropractic treatment for infants with breastfeeding dysfunction. Of the 6 case studies, 3 case series and 1 clinical trial found in this report there was a trend towards resolution of breastfeeding issues with chiropractic treatment of biomechanical imbalances, however, higher levels of evidence are needed.  **Adverse Events**: Not reported. |  |
| Glazener et al (2005)  Nocturnal Enuresis | | **Aim/Purpose:** To assess the effects of complementary interventions on nocturnal enuresis in children and to compare them with other interventions.  **Professional Context**: Chiropractic | | n= 1389 children (15 RCT'S)  **Inclusion Criteria:**  (1) All randomised or quasi-randomised trials of complementary and other miscellaneous interventions for nocturnal enuresis in children were included except those focused solely on daytime wetting (2) Comparison interventions could include no treatment, placebo or sham treatment, alarms, simple behavioural treatment, desmopressin, imipramine and miscellaneous other drugs and interventions. | | | **No. of databases**: 3  **Databases/Date range**: Cochrane Incontinence Group Specialised Register (searched 16 November 2006), the Traditional Chinese Medical Literature Analysis and Retrieval System (TCMLARS) (January 1984 to June 2004) and the reference lists of relevant articles. | | Methods of Cochrane Collaboration  **Outcomes**: number of wet nights | | | **Main Findings:** Complementary treatments such chiropractic may help, but the evidence was weak. Further research is needed.  Relative Risk (RR): (Reed 1994), in which the group given active chiropractic adjustment were less often wet at baseline, the children given active treatment were more likely to improve (RR versus sham adjustment 0.74, 95% CI 0.60 to 0.91, Comparison 04.02.01) and had fewer wet nights after treatment stopped (MD -1.8, 95% CI -2.96 to -0.64, Comparison 04.03.01). Active chiropractic adjustment had better results than sham adjustment (RR for failure or relapse after stopping treatment 0.74, 95% CI 0.60 to 0.91).  **Adverse Events:** Headache, stiff neck and lumbar spine pain were the adverse events; however, they could not be attributed to the treatments with certainty. |  |
|  | | **Aim/Purpose/Context** | | **Participants** | | | **Databases searched/Date range of database** | | **Critical Appraisal Tools used** | | | **Main Findings/Adverse Events** |  |
| Gleberzon et al (2012)  Multiple Conditions | | Aim/Purpose: (1) Conduct a search of the literature between 2007 and 2011 investigating the use of spinal manipulative therapy for paediatric health conditions  (2) Perform a systematic review of eligible retrieved clinical trials  **Professional Context**: Chiropractic | | n= 1980 (16 clinical trials)  **Inclusion criteria:**  (1) Subjects were age 18 or less (2) Studies involved more than two subjects (3) Treatments must have been administered by a chiropractor (4) Treatment administered was manual, high-velocity low-amplitude (HVLA) thrusting spinal manipulations (5) English language (6) Published between January 1980 and March 2011 (7) Prospective or retrospective studies including randomised controlled trials, controlled clinical/quasi-experimental trials, prospective cohort studies or retrospective case series (8) Studies using some type of outcome measure for determining the effect of chiropractic care (9) Published in peer-reviewed journal (10) Human subjects | | | **No. of databases**: 2  **Databases:** The Index of Chiropractic Literature and PubMed   **Date range:** 2007-2011 | | Grading criteria developed by Sackett  **Outcomes**: parent diaries for decreased crying time, cobb angle for scoliosis, frequency of wet nights for nocturnal enuresis, pulmonary functions and peak expiratory flow for asthma and improvement in symptoms for infantile colic | | | **Main Findings**: Studies that monitored both subjective and objective outcome measures of relevance to both patients and parents tended to report the most favourable response to spinal manual therapy, especially among children with asthma. Further research is clearly required in this area of chiropractic health care especially with respect to the clinical effectiveness of spinal manual therapy on paediatric back pain.  **Adverse Events**: No adverse events were reported. |  |
| Green et al (2019)  Multiple Conditions | | **Aim/Purpose:** Safer Care Victoria commissioned Cochrane Australia to undertake this systematic review of the effectiveness and safety of spinal manipulation in children under 12 years for any condition or symptom   **Professional Context**: Chiropractic, physiotherapy, osteopathy, manual therapists | | Effectiveness review: n=13 studies   Safety review: n= 10 studies   **Inclusion Criteria:**  (1) Children under 12 years of age  (2) Treated with spinal manipulation from any healthcare professional for any condition or indication  Effectiveness review: (1) Eligible studies included both randomised trials and observational studies, provided the observational studies included a comparator (e.g. non- randomised trial, cohort study, controlled before-and-after study).  Safety review: (1) Any article reporting adverse events was eligible, irrespective of the study type (i.e. trials, observational studies, case reports, etc.) | | | **No. of databases**: 7  **Databases**: PubMed, Index to Chiropractic Literature, Embase, Cochrane Central Register of Controlled Trials, CINAHL, Allied and Complementary Medicine (AMED) and Scopus.  **Date range**: 1960-2018 | | Nil critical appraisal was conducted  **Outcomes for effectiveness review:** Cerebral Palsy- wrist spasticity Infantile colic- crying time Nocturnal Enuresis - # of wet nights. Torticollis - symptoms improvement. ADHD - motion behavioural scores & visual spatial attention. Otitis Media- number of days with symptoms Asthma- lung function. Headache- percentage of days with headache Back pain- recurrence of spinal pain. Neck pain- recurrence of spinal pain.  **Outcomes for safety review**:  Adverse events categorised as mild (transient effects lasting less than 24 hours, e.g., crying or discomfort), moderate (requiring medical/general practitioner treatment) or severe (requiring hospital treatment, including disability or death) | | | **Main Findings**: Consistent with the findings of other systematic reviews, due to the paucity of studies and the lack of reported information on the specific treatment techniques employed, it is difficult to draw conclusions about the safety and effectiveness of spinal manipulation in children.  Relative Risk (RR): RR 0.98 (0.70 to 1.39) – 1 RCT for Torticollis; RR 1.26 (0.98 to 1.61) – 1 RCT (Haugen 2011) for neck/back pain. Compared to no SMT, spinal manipulation may increase recurrences of spinal pain (back, neck or both) among children (9- 15 years). If 10 children were followed for one year, 4 more recurrences may occur with spinal manipulation compared to no manipulation (95% CI: from 0 to 7 more recurrences, 238 participants) – Dissing 2018.  **Adverse Events**: Four studies described five individual cases of adverse effects from SMT in infants or children. Three were classified as severe and two as moderate. Of the three reports of a serious adverse event, one resulted in death. The technique employed in this case was described as the Vojta technique and involved forced active rotation and head retraction. Other serious adverse events were loss of consciousness with recovery and hospitalisation for drowsiness and weakness. |  |
|  | | **Aim/Purpose/Context** | | **Participants** | | | **Databases searched/Date range of database** | | **Critical Appraisal Tools used** | | | **Main Findings/Adverse Events** |  |
| Hawk et al (2007)  Multiple Conditions | | **Aim/Purpose:** To evaluate the evidence on the effect of chiropractic care, rather than spinal manipulation only, on patients with non-musculoskeletal conditions and to identify shortcomings in the evidence base on this topic, from a Whole Systems Research perspective  **Professional Context**: Chiropractic | | n= 179 papers addressing 50 different non-musculoskeletal conditions (122 case reports or case series, 47 experimental designs including 14 RCTs, 9 systematic reviews, and 1 a large cohort study)  **Inclusion Criteria:**  (1) Human subjects (2) English language (3) Peer-reviewed journal (4) Publication before May 2005 | | | **No. of databases**: 5  **Databases:** PubMed, Ovid, Mantis, Index to Chiropractic Literature, and CINAHL.  **Date range**: up to 2005 | | All randomised controlled trials (RCTs) were evaluated using the Scottish Intercollegiate Guidelines Network (SIGN) and Jadad checklists; a checklist developed from the CONSORT (Consolidated Standards of Reporting Trials) guidelines; and one developed by the authors to evaluate studies in terms of Whole Systems Research (WSR) considerations  **Outcomes**: patient-based measures (pain, function, health status) Nocturnal enuresis- number of wet nights Infantile colic- decreased crying time by crying diary  Asthma- pulmonary function Otitis Media- fewer recurrences of episodes | | | **Main Findings:** (1) Adverse effects should be routinely reported. (2) Evidence from controlled studies and usual practice supports chiropractic care as providing benefit to patients with asthma, cervicogenic vertigo, and infantile colic. Evidence was promising for potential benefit of manual procedures for children with otitis media. (3) There is insufficient evidence to make conclusions about chiropractic care for patients with other conditions.  **Adverse Events**: No serious events were reported, and several transient cases of muscle soreness and irritability were reported. |  |
| Hawk et al (2019)  Breastfeeding Difficulties | | **Aim/Purpose:** To survey the literature on manual treatments to correct musculoskeletal dysfunctions in infants with suboptimal breastfeeding.   **Professional Context**: Chiropractic and osteopathy | | n= 201 infants (27 articles included- 7 expert commentaries, 1 high-quality RCT, 1 low-quality cohort, 1 pilot study, 2 cross-sectional surveys, 5 narrative reviews and 10 case series or case reports)  **Inclusion Criteria:**  (1) English language (2) Human participants (3) Publications in peer reviewed journals, non-peer reviewed journals, books, webpages and conference proceedings | | | **No. of databases**: 2  **Databases**: PubMed and Index to Chiropractic Literature  **Date range:** up until 2018 | | Nil critical appraisal was conducted  **Outcomes**: LATCH (Latch, Audible swallowing, Type of nipple, Comfort, Hold) tool for improvement in breastfeeding | | | **Main Findings**: Based on the GRADE criteria, there is moderate positive evidence for the effect of manual therapy on suboptimal breastfeeding. Additional controlled studies of this topic are warranted. In terms of adverse events, published cases of serious adverse events in infants and children due to manual therapies are rare.  **Adverse Events**: Most articles did not state whether or not there were any adverse effects. |  |
| Hondras et al (2005)  Asthma | | **Aim/ Purpose:** To evaluate the evidence for the effects of manual therapies for treatment of patients with bronchial asthma **Professional Context**: Chiropractic | | n = 156 patients (in 3 RCT's)  **Inclusion criteria:** (1) Trials were included if they were randomised (2) Asthmatic children (up to age of 16 years) or adults (3) Examined one or more types of manual therapy (4) Included clinical outcomes with observation periods of at least two weeks | | | **No. of databases**: 8  **Databases**: EMBASE, CINAHL and MEDLINE and specialized databases Cochrane Complementary Medicine Field, Cochrane Rehabilitation Field, Index to Chiropractic Literature (ICL), and Manual, Alternative and Natural Therapy (MANTIS)  **Date range**: inception to 2001 | | Nil critical appraisal was conducted  **Outcomes:** lung function (such as vital capacity, forced expiratory volume in one second (FEV1), FEV1/FVC) ratio, hospital admissions, hospitalization days, emergency room visits, medication use, quality of life, and subjective symptoms. | | | **Main Findings**: There is insufficient evidence to support the use of manual therapies for patients with asthma. Adequately sized RCTs that examine the effects of manual therapies on clinically relevant outcomes need to be conducted and future trials should maintain observer blinding for outcome assessments, as well as report on the costs of care and adverse events.  **Adverse Events**: Reported no adverse serious events. |  |
|  | | **Aim/Purpose/Context** | | **Participants** | | | **Databases searched/Date range of database** | | **Critical Appraisal Tools used** | | | **Main Findings/Adverse Events** |  |
| Huang et al (2011)  Nocturnal Enuresis | | **Aim/Purpose:** To assess the effects of complementary interventions and others on nocturnal enuresis in children, and to compare them with other interventions  **Professional Context**: Chiropractic | | n= 2334 children (24 RCT's)  **Inclusion criteria:**  (1) All randomised or quasi-randomised trials of complementary and other miscellaneous interventions for nocturnal enuresis in children (up to age of 16 years) were included except those focused solely on daytime wetting.  (2) Comparison interventions could include no treatment, placebo or sham treatment, alarms, simple behavioural treatment, desmopressin, imipramine and miscellaneous other drugs and interventions. | | | **No. of databases**: 7  **Databases/date range:** PubMed (1950 to June 2010), EMBASE (1980 to June 2010), the Traditional Chinese Medical Literature Analysis and Retrieval System (TCMLARS) (1984 to June 2010), Chinese Biomedical Literature Database (CBM) (1975 to June 2010), China National Knowledge Infrastructure (CNKI) (1979 to June 2010), VIP database (1989 to June 2010), and the reference lists of relevant articles, all last searched 26 June 2010. | | Nil critical appraisal was conducted  Outcome Measures: number of wet nights | | | **Main Findings:** Current evidence does not support the use of spinal manipulation until proper randomised trials have demonstrated their effectiveness. Adverse effects of these therapies seemed to be generally mild.  **Adverse Events**: No adverse events reported in the chiropractic RCTs. |  |
| Humphreys, B. K. (2010)  Adverse Events | | **Aim/Purpose:** To update the clinical research literature from the 2007 report by Vohra, Johnston, Cramer and Humphreys on possible adverse events in children treated by spinal manipulation.  **Professional Context**: Chiropractic & Osteopathic | | n= 3 clinical studies, 1 systematic review with meta-analysis and 1 evidence report   **Inclusion Criteria:**  (1) Reviewed the literature from the 2007 report by Vohra, Johnston Cramer and Humphreys | | | **No. of databases:** 3  **Databases**: MEDLINE, PubMed and PubMed Central  **Date range**: 2004-2010 | | Nil critical appraisal was conducted   **Outcomes**: subjective reporting of adverse events | | | **Main Findings**: There is currently insufficient research evidence related to adverse events and manual therapy, therefore, more high-quality research specifically addressing adverse events and paediatric manual therapy is needed.  **Adverse Events**: No serious or catastrophic adverse events were reported. |  |
| Karpouzis et al (2010)  ADHD | | **Aim/Purpose:** The review seeks to answer the question of whether chiropractic care can reduce symptoms of inattention, impulsivity and hyperactivity for paediatric and adolescent ADHD.  **Professional Context**: Chiropractic | | No studies met the inclusion criteria  **Inclusion criteria:** (1) Levels I, II and III evidence (2) Chiropractic intervention studies (3) Children age 0-17 years, diagnosis of AD/HD consistent with DSM-III, DSM-IV, DSM-IV-TR or ICD-10 criteria (4) Diagnosis made by Paediatrician, Psychiatrist, Medical Doctor, Clinical or Educational Psychologist (5) Validated Psychometric Outcome Measure as recommended by the American Academy of Child and Adolescent Psychiatry (AACAP 2007),  (6) Full-text articles and English language | | | **No. of databases**: 8  **Databases:** Cochrane CENTRAL register of Controlled Trials, Cochrane Database of Systematic reviews, MEDLINE, PsycINFO, CINAHL, Scopus, ISI Web of Science, Index to Chiropractic Literature  **Date range:** inception to July 2009 | | All randomised controlled trials were evaluated using the Jadad score and a checklist developed from the CONSORT (Consolidated Standards of Reporting Trials) guidelines  **Outcomes**: severity of symptoms of inattention, impulsivity, and hyperactivity | | | **Main Findings**: The current finding for this systematic review has been classified as an 'empty review' since no studies met the inclusion criteria for this topic. This 'empty review' allows for the opportunity to learn from the excluded studies. To date there is insufficient evidence to evaluate the efficacy of chiropractic care for paediatric and adolescent ADHD. The claim that chiropractic care improves paediatric and adolescent ADHD, is only supported by low levels of scientific evidence. If chiropractic care for ADHD is to continue, more rigorous scientific research needs to be undertaken to examine the efficacy and effectiveness of chiropractic treatment. Adequately-sized RCTs using clinically relevant outcomes and standardised measures to examine the effectiveness of chiropractic care verses no-treatment/placebo control or standard care are needed”.  **Adverse Events:** No adverse events reported. |  |
|  | | **Aim/Purpose/Context** | | **Participants** | | | **Databases searched/Date range of database** | | **Critical Appraisal Tools used** | | | **Main Findings/Adverse Events** |  |
| Kronau et al (2016)  Autism Spectrum Disorder | | **Aim/Purpose:** To answer whether new research on the topic of the clinical benefits of manual therapy of the musculoskeletal system in children diagnosed with autism spectrum disorder has been updated and formerly assess the available evidence for methodological quality and risk of bias, as well as giving detailed recommendations for further research on the topic.  **Professional Context**: Chiropractic | | n = 1 randomised clinical trial (uncontrolled), 1 case series and 11 case reports  **Inclusion criteria:**  (1) Individuals aged 0-21 years (2) Studies published in English, German, or French, a diagnosis of autism or ASD (3) Study designs of randomised clinical trial, case control studies, case series, case reports, and single subject studies, which include manual therapeutic interventions of the musculoskeletal system. | | | **No. of databases**: 12  **Databases:** Bio Med Central, Chiropractic Library Collaboration, Clinical Trials, Cochrane library, Dimdi, EBSCO host, PubMed, PubMed central, Medline Plus, Osteopathic research Digital repository, Osteopathic Research Web, and Physiotherapy Evidence Database.   **Date range**: Database start up until October 2015 | | Downs and Black tool and Quality of reporting for case series and case reports was assessed with the appropriate checklists provided by the Quality and Transparency Of health Research (EQUATOR) network.  **Outcomes:** ATEC; X-rays; spinal palpation; leg length difference; palpation of the spine, modified autism rating scale, thermal scan; SEMG, palpation in motion | | | **Main Findings:** Literature on the effects of chiropractic interventions to the musculoskeletal system of autistic children and young adults appears to be favourable with respect to the severity of their symptoms, however, results of this review have to be interpreted with great caution, as the majority of identified studies were case reports. Due to a lack of statistical data and poor study design of the included randomised clinical trial, no comments on the effectiveness/efficacy of the chiropractic intervention can be made. Further feasibility and pilot research are needed to lay the foundation for good quality clinical trials of spinal manipulation in the autistic child population.  **Adverse Events:** Not reported. |  |
| Lucassen, P. (2010)  Infantile Colic | | **Aim/Purpose**: To answer the following clinical question: "What are the effects of treatments for colic in infants?"  **Professional Context**: Chiropractic | | n= 7 systematic reviews, RCTs, or observational studies   **Inclusion criteria:**  (1) Published systematic reviews and RCTs in any language (2) Containing at least 20 individuals of whom more than 80% were followed up. | | | **No. of databases**: 3+  **Databases**: Medline, Embase, The Cochrane Library, and other important databases  **Date range:** up to 2009 | | GRADE evaluation of the quality of evidence for interventions  **Outcomes:**  Crying time | | | **Main Findings**: Spinal manipulation significantly reduced crying compared with simethicone; however, it is unclear whether reduced crying reflected the effects of interventions or spontaneous improvement.  **Adverse Events:** Not reported. |  |
| Parnell et al (2019)  Multiple Conditions | | **Aim/Purpose:** To evaluate the use of manual therapy for clinical conditions in the paediatric population, assesses the methodological quality of the studies found, and synthesizes findings based on health condition. This systematic review also assessed the reporting of adverse events within the included studies and compared their conclusions to those of the UK Update report.  **Professional Context**: Chiropractic and osteopathic | | n= 50 studies  **Inclusion criteria:**  (1) Children under the age of 18 years (2) Treatment using manual therapy (3) Any type of healthcare profession (4) Published between 2001 and March 31, 2018 (5) English language only | | | **No. of databases**: 6  **Databases:** PubMed, Cochrane Library, Medline complete, CINAHL complete, ScienceDirect, McCoy Press, Index to Chiropractic Literature, and National Guideline Clearinghouse  **Date range**: 2001-2018 | | Nil critical appraisal conducted  **Outcomes:** (1) Asthma- peak expiratory flow and inhaler use, questionnaires assessing quality of life (QOL), asthma severity, & improvement.  (2) Otitis Media- symptoms (3) Cerebral Palsy- Gross Motor Function Measure 66 (GMFMM-66) and QOL Child Health Questionnaire (CHQ) PF50 (4) ADHD- visual spatial attention (5) Headache- days with a headache, total duration of headache (hrs), days missing school, days with necessity of analgesic medication, and intensity of headache. (6) Breastfeeding  (7) Torticollis- symptoms (8) Scoliosis- cobb angle (9) Nocturnal Enuresis- wet nights (10) Low back pain- pain severity (11) Infantile Colic- crying time (12) Autism- Modified Autism Research Institute Outcomes Survey (13) Dysfunctional voiding- symptom improvement. | | | **Main Findings:** Inconclusive (favourable) evidence for: ADHD (using OMT), autism (using CMT), asthma (using OMT), dysfunctional voiding (using OMT), infantile colic (using OMT), otitis media (using OMT), breastfeeding (using CMT).  Inconclusive (unclear) evidence for: asthma (using CMT), autism (using VOMT), cerebral palsy (using OMT), headache (using CMT, OMT and MT), infantile colic (using CMT), low back pain (using MT), otitis media (using CMT), nocturnal enuresis (using CMT), scoliosis (using CMT) and upper cervical dysfunction (using MT).  Inconclusive unfavourable evidence for:  Scoliosis (using OMT) and torticollis (using MT).  MT= manual therapy; CMT= chiropractic manual therapy; OMT= osteopathic manual therapy; VOMT= visceral osteopathic manual therapy.  **Adverse Events**: Not reported. |  |
|  | | **Aim/Purpose/Context** | | **Participants** | | | **Databases searched/Date range of database** | | **Critical Appraisal Tools used** | | | **Main Findings/Adverse Events** |  |
| Pohlman & Holton-Brown (2012)  Otitis Media | | **Aim/Purpose:** Review the literature for otitis media in children, outlining the diagnosis of OM, SMT description, and adverse event notation.  **Professional Context**: Chiropractic and osteopathic | | n= 49 articles inclusive of 17 commentaries, 15 case reports, 5 case series, 8 reviews and 4 clinical trials  **Inclusion criteria:**  (1) English language (2) Addressed otitis media (3) Involved human participants 6 years or younger (4) Addressed spinal manual therapy | | | **No. of databases**: 6  **Databases:** PubMed, Cochrane Library, Cumulative Index to Nursing and Allied Health, Index to Chiropractic Literature, The Allied and Complementary Medicine, and Alt Health Watch  **Date range**: inception through March 11th | | Checklist developed by the Canadian Medical Association Journal to assess the quality of case reports, Yang et al developed the checklist for case series, CONSORT (Consolidated Standards of Reporting Trials) was used for the clinical trials and QUORUM (Quality of Reporting of Meta- analyses) was used to evaluate the review articles  **Outcomes:** Decreased Symptoms | | | **Main Findings**: Limited quality evidence for the use of spinal manual therapy for children with otitis media. Currently, there is no evidence to support or refute its use and no evidence to suggest that spinal manipulation produces serious adverse effects for children with otitis media. More rigorous studies are needed to provide higher levels of evidence.  **Adverse Events:** No serious adverse events were found. Minor transient adverse effects were noted in 1 case series article and 2 of the clinical trials. |  |
| Romano &  Negrini (2008)  Adolescent idiopathic scoliosis | | **Aim/purpose:** To verify the data existing in the literature on the efficacy of using manual therapy to treat adolescent idiopathic scoliosis (AIS)  **Professional Context**: Chiropractic and osteopathic | | n= 3 papers  **Inclusion criteria:**  (1) Any kind of research (2) Diagnosis of adolescent idiopathic scoliosis (3) Patients treated exclusively by one of the procedures established as a standard for this review (chiropractic manipulation, osteopathic techniques, massage) (4) Outcome in Cobb degrees | | | **No. of databases**: 5  **Databases**: Medline, Embase, CINAHL, Cochrane Library, Pedro   **Date range**: not stated | | Nil critical appraisal was conducted  **Outcomes**: cobb angle - visual evaluation and palpation, including shoulder asymmetry, axillae asymmetry and position of scapula angle. | | | **Main Findings**: No articles satisfied all the required inclusion criteria because they were characterized by a combination of manual techniques and other therapeutic approaches. The lack of any kind of serious scientific data does not allow us to draw any conclusion on the efficacy of manual therapy as an efficacious technique for the treatment of Adolescent idiopathic scoliosis.  **Adverse Events:** No adverse events or harms reported. |  |
| Theroux et al (2017)  Adolescent idiopathic scoliosis | | **Aim/Purpose:** Perform a systematic review of clinical trials of spinal manipulative therapy for adolescent idiopathic scoliosis  **Professional Context:** Chiropractic, osteopathic, physical therapy | | n= 4 studies  **Inclusion criteria:** (1) Prospective trials (RCTs and other studies) that evaluated spinal manipulative therapy (e.g., chiropractic, osteopathic, physical therapy) for adolescent idiopathic scoliosis. | | | **No. of databases**: 3  **Databases**: PubMed, CINHAL, and CENTRAL  **Date range**: until June 2016 | | Cochrane risk of bias tools were used to assess the quality of the included studies  **Outcomes:** Cobb angle  Secondary outcome measures included: Aesthetics, pain intensity, physical disability, quality of life, and adverse events | | | **Main Findings:** There is currently insufficient evidence to establish whether spinal manipulative therapy may be beneficial for adolescent idiopathic scoliosis. The results of the included studies suggest that spinal manipulative therapy may be a promising treatment, but these studies were all at substantial risk of bias. Further high-quality studies are warranted to conclusively determine if spinal manipulative therapy may be effective in the management of adolescent idiopathic scoliosis.  **Adverse Events:** Only mentioned in 1 of the included studies, which noted that 2 benign reactions were reported over the course of the study. |  |
| Todd et al (2015)  Adverse Events | | **Aim/Purpose:** Review the literature for cases of adverse events in infants and children treated by chiropractors or other manual therapists, identifying treatment type and if a pre-existing pathology was present."  **Professional Context**: Chiropractors, physiotherapists, practitioners | | n= 31 articles  **Inclusion criteria:**  (1) Adverse events documented by any manual therapy health provider (i.e., chiropractors/Doctor of Chiropractic, physical therapists, medical physicians, doctors of osteopathy, and other manual therapists) (2) English only (3) Clear discussion of adverse events (mild, moderate, or severe) | | | **No. of databases**: 14  **Databases**: EMBASE, AMED, BIOSIS previews, MEDLINE, Maternity and Infant Care, OVID, CINAHL, PsycInfo, PubMed, INDEX to Foreign and Legal Periodicals, SCOPUS, Science Direct, Index to Chiropractic Literature, and PubMed Central databases   **Date range**: inception of searchable bibliographic databases to March 2014 | | Nil critical appraisal was conducted  **Outcomes:**  Adverse events | | | **Main Findings:** Published cases of serious adverse events in infants and children receiving chiropractic, osteopathic, physiotherapy, or manual medical therapy are rare. The 3 deaths that have been reported were associated with various manual therapists; however, no deaths associated with chiropractic care were found in the literature to date. Because underlying pre-existing pathology was associated in a majority of reported cases, performing a thorough history and examination to exclude anatomical or neurologic anomalies before applying any manual therapy may further reduce adverse events across all manual therapy professions.  **Adverse Events**: 12 articles reported 15 serious adverse events. 3 deaths occurred under the care of various providers (1 physical therapist, 1 unknown practitioner, and 1 craniosacral therapist). 12 serious injuries were reported (7 chiropractors/Doctor of Chiropractic, 1 medical practitioner, 1 osteopath, 2 physical therapists, and 1 unknown practitioner). Underlying pre-existing pathology was identified in a majority of the cases. |  |
|  | | **Aim/Purpose/Context** | | **Participants** | | | **Databases searched/Date range of database** | | **Critical Appraisal Tools used** | | | **Main Findings/Adverse Events** |  |
| Vaughn et al (2012)  Multiple Conditions | | **Aim/Purpose:** Evaluate the evidence for spinal manual therapy interventions in patients 4–17 years old with headaches and/or mechanical spinal pain  **Professional Context:** Chiropractic, physiotherapist, osteopathy and manual therapist | | n= 2 RCTs and 2 prospective cohort studies   **Inclusion criteria:**  (1) Age 4–17 years (2) Diagnosis of spinal pain and/ or headaches of mechanical origin (3) Intervention of spinal manual therapy (4) Include at least one outcome measure for pain, function, or quality of life | | | **No. of databases**: 6  **Databases**: MEDLINE, CINAHL, Cochrane Central Register of Randomised Control Trials, PEDro, PubMed, and Sports Discus  **Date range**: the past 15 years from 2012 | | GRADE (Grading of Recommendations Assessment, Development and Evaluation) criteria  **Outcomes:** Pain, function, and/or disability changes by Roland Morris score mean (SD) and VAS score | | | **Main Findings**: There is very little data in the literature to support or refute the use of spinal manual therapy interventions in paediatric patients. Further research is required to establish a strong evidence-based foundation for use of these interventions in children.  **Adverse Events**: No adverse events reported. |  |
| Vohra et al (2007)  Adverse Events | | **Aim/Purpose:** Systematically identify and synthesize available data on adverse events associated with paediatric spinal manipulation.  **Professional Context:** Chiropractic, physiotherapy and medical doctor | | n= 13 studies, 2 randomised trials 11 observational reports  **Inclusion criteria**: (1) Primary investigation of spinal manipulation (e.g., observation studies, controlled trials, surveys) (2) Children aged 18 years or younger (3) Reported data on adverse events | | | **No. of databases:** 8  **Databases**: Central (second quarter, 2004), Medline (1966 –2004), PubMed (1966–2004), Embase (1988–2004), CINAHL (1982–2004), AltHealthWatch (1990–2004), MANTIS (1900–2005), and ICL (1985–2004)   **Date range**: inception to June 2004 | | Nil critical appraisal was conducted  **Outcomes**: Adverse events | | | **Main Findings**: Serious adverse events may be associated with paediatric spinal manipulation, however, neither causation nor incidence rates can be inferred from observational data. Although serious adverse events have been identified, their true incidence remains unknown due to underreporting.  **Adverse Events:** 9 cases involved serious adverse events (e.g., subarachnoid haemorrhage, paraplegia), 2 involved moderately adverse events that required medical attention (e.g., severe headache), and 3 involved minor adverse events (e.g., midback soreness). |  |
|  | | | | | | | | | | | | |  |
| **Data Extraction for RCT’s** | | | | | | | | | | | | |  |
| **Study** | **Aim / Design** | | | | **Participants** | | | **Intervention** | | | **Main Findings & Adverse Events** | | |
| Balon et al (1998)  Asthma | **Aim**: To assess objective and subjective outcomes in children with asthma who were treated with active or simulated chiropractic manipulation.  **Design**: RCT | | | | n= 80 children   **Age** = 7 - 16 years  **Inclusion Criteria:**  (1) Children 7 - 16 years who had had asthma (diagnosed by a physician) for > one year  (2) Symptoms requiring the use of a bronchodilator at least 3 times weekly  (3) Evidence of vertebral subluxation on palpation | | | Group 1: Active chiropractic manipulation and gentle soft tissue therapy in addition to usual medical care (n= 38).   Group 2: Simulated chiropractic manipulation (n=42). Soft-tissue therapy and gentle palpation to spine, paraspinal muscles, and shoulders, in addition to usual medical care.  Duration of intervention: 4 months  **Context**: Chiropractic  **Outcomes**: Change from base line in the peak expiratory flow, changes in airway responsiveness, FEV1, symptoms of asthma, need for inhaled b-agonists, use of oral corticosteroids, quality of life, and overall satisfaction with treatment. | | | **Statistics:** Changes in morning peak expiratory flow for each subject were expressed as the percent change from the mean of the measurements. All analyses were performed with SPSS. All P values are two-tailed.  **Main Findings:** Small increases in peak expiratory flow in the morning and the evening in both treatment groups, with no significant differences between the groups in the degree of change from base line (2 months p=0.49; 4 months p=0.82). Symptoms of asthma and use of b-agonists decreased, and the quality of life increased in both groups, with no significant differences between the groups (symptoms; p=0.59 at 2 months and p=0.84 at 4 months, b-agonist use; p=0.55 and p=0.35). No significant changes occurred in spirometric measurements or airway responsiveness. In children with mild or moderate asthma, the addition of chiropractic spinal manipulation to usual medical care provided no benefit.  **Adverse Events**: No adverse events (apart from exacerbations of asthma) occurred during the study. | | |
| Borusiak et al (2009)  Headache | **Aim:** to investigate the efficacy of SMT in children and adolescent with recurrent headache.  **Design:** Prospective, randomised, placebo-controlled and blinded trial | | | | n= 52 children and adolescents   **Age**: 7- 15 years  **Inclusion Criteria**: In relation to all children/adolescents presenting to outpatient clinic at one of the neuropediatric departments in Wuppertal, Oberhausen, and Wesel (1) With headaches for > 6 months at least once a week  (2) 7-15 years of age | | | Group 1: SMT group (n=24). Cervical high-velocity, low-amplitude lateral directed manipulation without rotation or extension.  Group 2: Placebo group (n=28). Light touch of specific spinal segments. Low amplitude, high-velocity thrust, given the impression of a cervical manipulation that however was not directed to correct the assumed cervical blockage.  **Context**: Manual Therapist   **Outcomes**: Percentage of days with headache, total duration of headache, days with school absence due to headache, consume of analgesics, intensity of headache. | | | **Statistics**: Proportions compare using chi-square tests with continuity correction. Analysis of changes from baseline to follow-up period using paired t-test. Two-sided significance tests used throughout.  **Main Findings:** Both the placebo and SMT group demonstrated reduced frequency of headaches. No significant difference between groups existed (p=>0.05) with respect to the main outcome measure suggesting that the changes were not related to SMT.  **Adverse Events:** Only minor adverse events reported without significant or clinically relevant differences comparing the patients with SMT and sham treatment with hot skin and dizziness being reported most often. All patients recovered spontaneously with no reported sequelae. The trial was ceased before reaching their planned participant rates, due to a higher-than-expected level of complaints. | | |
| Bronfort et al (2001)  Asthma | **Aim**: To determine if chiropractic spinal manipulative therapy (SMT) in addition to optimal medical management resulted in clinically important changes in asthma-related outcomes in children. To assess the feasibility of conducting a full-scale, randomised clinical trial in terms of recruitment, evaluation, treatment, and ability to deliver a sham SMT procedure.  **Design:** RCT | | | | n= 36  **Age**: 6 - 17 years  **Inclusion Criteria:**  (1) Aged 6 to 17 years  (2) Mild or moderate persistent asthma as diagnosed by the paediatric pulmonologist using national guideline criteria | | | Group 1: Spinal manipulation (n=22). Drop mechanism and manual spinal thrusting technique used over a vertebral osseous process, muscle, or ligament. Carried out with a high-velocity, low-amplitude thrust (short-lever technique) in addition to optimal medical care Group 2: Sham spinal manipulation (n=11): light manual contact to the spine with no manipulative thrust. Drop mechanism was used, in addition to optimal medical care.   Duration: 20 chiropractic treatment sessions were scheduled during the 3-month intervention phase.   **Context:** Chiropractic   **Outcomes:** Pulmonary function tests, patient- and parent- or guardian-rated asthma-specific quality of life, asthma severity, and improvement, AM and PM peak expiratory flow rates, and diary-based day and night-time symptoms | | | **Statistics:** Student t tests comparing baseline values with posttreatment values at 12 weeks.   **Main Findings**: At the end of the 12-week intervention phase, objective lung function tests and patient-rated day and night-time symptoms based on diary recordings showed little or no change. Of the patient-rated measures, a reduction of approximately 20% in β2 bronchodilator use was seen (P = .10). The quality-of-life scores improved by 10% to 28% (P < .01). Asthma severity ratings showed a reduction of 39% (P < .001), and there was an overall improvement rating corresponding to 50% to 75%. After 3 months of combining chiropractic SMT with optimal medical management for paediatric asthma, the children rated their quality of life substantially higher and their asthma severity substantially lower. These improvements were maintained at the 1-year follow-up assessment. There were no important changes in lung function or hyperresponsiveness at any time.  **Adverse Events**: Not reported. | | |
| **Study** | **Aim / Design** | | | | **Participants** | | | **Intervention** | | | **Main Findings & Adverse Events** | | |
| Browning & Miller (2008)  Infantile Colic | **Aim:** To compare two interventions in the treatment of infant colic.  **Design**: RCT | | | | n= 48   **Age**: less than 8 weeks of age  **Inclusion Criteria:**  (1) Less than 8 weeks of age (2) Birth weight equal to > 2500  (3) Born at or after 38 weeks gestation (4) Cry for 3h or more per day with one or more inconsolable crying episodes for at least four of the previous 7 days (5) Show typical restless behaviour (i.e. motor unrest, flexing knees against abdomen, extending the trunk, neck, and extremities) | | | Group 1 (n=22): Received spinal manipulative therapy (SMT) appropriate for neonates and appropriate to the age of the patient as indicated on examination  Group 2 (n=21): Received occipito-sacral decompression (OSD)- occiput and the sacral base are contacted simultaneously, and gentle distraction is applied for up to 30s.  **Context:** Physiotherapy  **Outcomes:** Change in daily hours of crying | | | **Statistics**: Data were analysed using SPSS version. Comparison of categorical data was performed using the Chi-squared test and of continuous data using the unpaired t-test. To test changes within the groups, the paired t-test was used.   **Main Findings**: At day 7 and day 14, mean hours of crying per day were significantly reduced in both groups (Day 7 SMT, by 2.1 h/day, p=<0.001; Day 7 OSD, by 2.0 h/day, p=<0.001 and Day 14 SMT, by 3.1 h/day, p=<0.001; Day 14 OSD, by 2.5 h/day, p=<0.001). At day 14, mean hours of sleep per day were significantly increased in both groups (SMT, by 1.7 h/day, p=<0.01; OSD, by 1.0h/day, p=<0.01). Four weeks after completion of the treatment trial, colic had resolved in 82% of the SMT group and 67% of the OSD group. Both treatments appear to offer significant benefits to infants with colic. Infants treated by SMT or OSD cried less and slept more after 2 weeks of treatment. There were no differences in outcomes between the two treatment approaches. Although the participants completed the trial of therapy prior to the usual age of remission for infant colic, the natural course cannot be ruled out.   **Adverse Events:** Not reported. | | |
| Cabrera- Martos et al (2016)  Plagiocephaly | **Aim:** To evaluate the effects of a therapeutic approach based on manual therapy as an adjuvant option on treatment duration and motor development in infants with severe non-synostotic plagiocephaly.  **Design:** Prospective, randomised controlled pilot study | | | | n= 46 infants  **Age**: 4-8 months  **Inclusion Criteria:**  (1) Clinical diagnosis of non-synostotic plagiocephaly (2) Facial asymmetry (3) Ear misalignment  (4) 4 - 8 months of age | | | Group 1 (n=23): This was based on a conservative approach including positional changes and the use of an orthotic helmet. It consisted of positional changes from supine, prone, lateral and sitting positions.  Group 2 (n=23): In addition to conservative treatment (positional changes and orthotic helmet), decompression techniques were used to normalise tissue around cranial sutures. The physical therapist guides the segments away from one another in a gentle distraction force maintaining this position until the tension released and improving the motion of the joints, mainly the spheno-occipital, the atlanto-occipital synchondrosis and the sacrum (1 x 45min/week mobilisation session.  **Context**: Physiotherapy   **Outcomes**: duration of treatment (days) and the motor development at baseline and discharge assessed with the Alberta Infant Motor Scale (AIMS). | | | **Statistics:** Data analysed using SPSS. Descriptive statistics (mean ± standard deviation) used to determine the characteristics of the infants included in the study. The statistical distribution of the data was initially analysed using the Shapiro-Wilk W test. Results were analysed for significance using the Student’s t test for parametric and the Mann-Whitney U test for nonparametric variables.   **Main Findings:** No significant between-group differences were found in motor development scores at baseline. Significant (p < 0.001) between group differences in treatment duration were observed, being shorter in the experimental group (109.84 +14.45 days) compared to control group (148.65 + 11.53 days). Significant differences were found in the AIMS subscales with higher scores in the experimental group however both groups were in the ‘normal motor behaviour’ range at time of discharge. The results show the feasibility of manual therapy for severe non-synostotic plagiocephaly, reducing the treatment duration and obtaining an adequate motor development score of the infants although it is important to remember that without the additional treatment, the infants still reached normal motor behaviour scores.  **Adverse Events**: Nil adverse events reported. | | |
| Dissing et al (2018)  Neck and Back Pain | **Aim:** To investigates the effectiveness of adding manipulative therapy (MT) to other conservative care for spinal pain in a school-based cohort of Danish children aged 9–15 years.  **Design:** RCT | | | | n=~283 children (invited), 243 (enrolled and randomised), 238 (analysed).  **Age**: 9-15 years (mean: 12.6 years)  **Inclusion Criteria:**  (1) Pain in neck or back equal to or > 3 on an 11-box numerical rating scale for more than three days | | | Group 1 (n=116): advice, exercise and soft tissue treatment (Non-MT)  Group 2 (n=122): advice, exercises and soft tissue treatment plus spinal manipulative therapy (MT)  **Context**: Chiropractic  **Primary outcome:** number of recurrences of spinal pain (3-27 months follow-up)  **Secondary outcomes**: duration of spinal pain, episode length, change in pain intensity (after 2 weeks) and Global Perceived Effect (after 2 weeks). | | | **Statistics:** Robust standard errors were used to take a violation of the distributional assumption into account. Intervention effects will be expressed as incidence rate ratio.  **Results:** No significant difference was found between groups in the primary outcome (control group median 1 (IQR 1-3) and intervention group 2 (IQR 0-4), p=0.07). There were no significant differences between groups in the average episode length (p=0.21), total number of pain weeks (p=0.22) or change in pain intensity (p=0.76), Children in the manipulative therapy group reported higher Global Perceived Effect: OR 2.22, (95% CI 1.19 to 4.15).  **Adverse Events:** Nil adverse events reported. | | |
| **Study** | **Aim / Design** | | | | **Participants** | | | **Intervention** | | | **Main Findings & Adverse Events** | | |
| Evans et al (2018)  Low Back Pain | **Aim**: To test the comparative effectiveness of SMT plus exercise therapy (ET) vs ET alone for individuals 12 to 18 years of age with recurrent or chronic LBP.  **Design:** RCT | | | | n=185 adolescents   **Age**: 12 -18 years   **Inclusion Criteria:**  (1) Adolescents (12-18 years of age) with subacute recurrent or chronic, nonspecific LBP (severity 3/10) with or without leg pain. | | | Group 1 (n=40): The goal of the ET (Exercise Therapy) program was to help adolescents manage their LBP and prevent future occurrences. The ET program included self-care education, supervised exercise, and instructions for home exercise. Participants attended 8 to 16, 45 minutes sessions with an exercise therapist or licensed chiropractor no more than 2 times per week  Group 2 (n=38): The goal of the combined SMT (Spinal Manipulative Therapy) 1 ET program was to enhance patients’ ability to exercise by providing treatment to the lumbar vertebral or sacroiliac joints in an effort to increase mobility and decrease pain. Participants attended 8- to 16-, 10- to 20- minute study visits with experienced licensed chiropractors, no more than 2 times per week.  **Context**: Orthopaedic doctor specialising in manual therapy   **Outcomes**: Self-reported typical level of LBP severity, disability, quality of life, medication use, patient- and caregiver-rated improvement, and satisfaction. | | | **Statistics**: Outcomes were analysed using longitudinal linear mixed effect mode.  **Main Findings**: For adolescents with chronic LBP, spinal manipulation combined with exercise was more effective for reducing low back pain severity than exercise alone over a 1-year period (p=0.007). Whilst no differences in low back pain severity existed directly after treatment (MD – 0.5; p=0.08), the largest benefits for reducing LBP severity were evident at 6 months (MD – 1.1; p=0.001) and 12 months (MD – 0.8; p=0.009). At 26 weeks, SMT with ET performed better than ET alone for disability (p=0.04) and care-giver rated improvement (p=0.02). At 12 months disability associated with LBP remained significantly better for the group who received SMT in addition to ET (p = 0.048) as did the parent reported improvement scores (p = 0.03). The SMT with ET group reported significantly greater satisfaction with care at all time points (p<0.02). There was not significant difference in pain medication use between groups through the duration of the study (p=0.22) and the addition of SMT did not significantly change Quality of Life (QOL).  **Adverse Events**: Two serious adverse events occurred during the course of the trial. Both occurred in the SMT ET group after intervention and were classified as unrelated to study interventions. One participant developed appendicitis and had an appendectomy. Another participant was hospitalised because of renal issues related to type I diabetes. Minor self-limiting adverse events during the 12 weeks of intervention were reported with approximately equal frequency in both groups. The more commonly reported adverse events associated with the addition of SMT were unusual or increased soreness (54%) and different type of pain (34%), and increased back pain severity (22%). | | |
| Haugen et al (2011)  Torticollis | **Aim:** To evaluate measurement methods and examine the short-time effect of manual therapy in addition to child physiotherapy.  **Design:** RCT (Pilot)  (Double blinded) | | | | n = 32 infants  **Age**: 3 – 6 months  **Inclusion Criteria:** (1) diagnosis of torticollis  (2) reduced mobility in the neck, in at least one axial plane. | | | Group 1: Control – physiotherapy (encouragement of symmetrical motor performance through a variety of methods.  Group 2: Intervention – physiotherapy + manual therapy (moderate force manipulation conducted with the child supine and the head in neutral position, without extension of any structures.  8 recruited infants not included as they were either having alternative manual therapy or had full range despite asymmetric movement patterns.  **Context:** Physiotherapy.  **Outcomes**: (1) Change in torticollis symptoms (primary); (2) Passive lateral flexion to the affected side (secondary); (3) Active lateral flexion against gravity to the affected side (head righting) (secondary). | | | **Main Findings**: No significant tendency to greater improvement in lateral flexion (p = 0.116)) and head righting reaction (p = 0.092) in the intervention group. Physiotherapy with manual therapy (cervical manipulation) is not significantly better than physiotherapy alone at weeks 2 (p = 0.313) or 8 (p = 0.848).  Adverse Events: Not reported. | | |
| Kachmar et al (2018)  Cerebral Palsy | **Aim:** To investigate the short-term effects of spinal manipulation (SM) on wrist muscle spasticity and manual dexterity in participants with cerebral palsy (CP).  **Design**: RCT | | | | n= 78  **Age**: 7-18 years   **Inclusion Criteria:**  (1) Spastic forms of CP  (2) Age 8 to 18 years  (3) Hand function level I to III according to the Manual Ability Classification System (MACS) | | | Group 1 – Experimental (receiving Spinal Manipulation)  Intervention was cervical, thoracic and lumbar manipulation.  Thoracic = posterior-anterior pressure with counter-clockwise rotational force in prone driving the right hand away from the left. High-velocity, low-amplitude (HVLA) thrust applied in vertical direction when patient exhaled.  Lumbar = lateral recumbent position with upper leg flexed at hip and knee, lower leg straight, lumber joints in slight extension. Joint pretension followed by rotational force to shoulder and thigh with HVLA thrust to facet joints in posterior to anterior direction. Cervical = seated position with head flexed sideways and slightly rotated, traction and side-bending force before applying HVLA thrust.  Cervical and Lumbar manipulations performed bilaterally.  Group 2 – Sham of Spinal Manipulation – Control using same positions with a gentle thrust.  **Context:** Medical Doctor  **Primary Outcomes:** Muscle spasticity in the wrist assessed quantitatively with a NeuroFlexor device (measured at baseline and post intervention)  Secondary Outcome Measure: Manual dexterity assessed with the Box and Block test (measured at baseline and post intervention). | | | **Main Findings:**  - Experimental group: muscle spasticity was significantly reduced (p= 0.002). - Control group: reduction in spasticity was negligible.  - The between-group difference in change of muscle spasticity was statistically significant (p=0.034) but change in dexterity was not significantly different between groups (p=0.28).  Spinal manipulation may help to reduce spasticity in participants with cerebral palsy for the short term. Long term effects were not studied.  **Adverse Events:** Not reported. | | |
| **Study** | **Aim / Design** | | | | **Participants** | | | **Intervention** | | | **Main Findings & Adverse Events** | | |
| Lynge et al (2021)  Headaches | **Aim:** To investigate the effectiveness of chiropractic spinal manipulation versus sham manipulation in children aged 7–14 with recurrent headaches.  **Design:** RCT | | | | n= 199 (194 analysed)  **Age**: 7-14 years  **Inclusion Criteria:**  (1) At least one episode of headache per week for the previous 6 months  (2) At least one musculoskeletal dysfunction | | | All participants received standard oral and written advice to reduce headaches plus:  Active treatment group (n=99): chiropractic spinal manipulation  Control group (n=100): sham manipulation for 4 months  Duration: number and frequency of treatment was based on individual evaluation for the treatment group and the control group received approximately 8 visits during the treatment period  **Context**: Chiropractic  **Outcomes:** number of days with headache, pain intensity and medication reported weekly by text message and global perceived effect after 4 months. | | | **Main Findings:** Chiropractic spinal manipulation resulted in significantly fewer days with headaches (reduction of 0.81 vs. 0.41 for sham manipulation, p= 0.019) and better global perceived effect (dichotomized into improved/not improved, OR = 2.8 (95% CI:1.5–5.3) compared with sham manipulation. There was no difference between groups for pain intensity during headache episodes (p=0.930). No significant differences between groups for medication use were found (p=0.28).  **Adverse Events:** Not reported. | | |
| Miller, Newell and Bolton (2012)  Infantile Colic | **Aim:** To determine the efficacy of chiropractic manual therapy for infants with unexplained crying behaviour and if there was any effect of parental reporting bias.  **Design:** Pragmatic, single blind, RCT | | | | n= 104 (102 analysed)   **Age:** < 8 weeks  **Inclusion Criteria:**  (1) Infants with infantile colic presenting to a chiropractic teaching clinic at the AngloEuropean College of Chiropractic (2) Infants younger than 8 weeks, born at a gestational age of 37 weeks or later (3) Birth weight of 2500 grams or more  (4) No signs of other conditions or illness | | | Group 1: Treated up to 10 days - manual therapy 2N of force without rotation to spinal joints and paraspinal muscles (parent aware)  Group 2: Treated up to 10 days - manual therapy 2N of force without rotation to spinal joints and paraspinal muscles (parent unaware)  Group 3: Not treated (parent unaware)  **Context**: Chiropractic (mobilisation)  **Outcomes:** daily crying diary completed by parents over a period of 10 days | | | **Main Findings**: Chiropractic manual therapy improved crying behaviour in infants with colic. The odds of improvement for those who were treated compared to those who were not treated in days 0-6 were not significantly different. There was an increase in odds of improvement for those who were treated compared to those who were not treated at day 8 [OR] 8.1 (95% confidence interval [CI], 1.4-45) and at day 10, [OR] 12.0 (2.1-68) using the cut-offs of 2 or less hours of crying per day with similar results for the threshold of 30% change in crying.  The findings showed that knowledge of treatment by the parent did not appear to contribute to the observed treatment effects in this study, therefore, it is unlikely that observed treatment effect is due to bias on the part of the reporting parent.  **Adverse Events**: No adverse events were reported. | | |
| **Study** | **Aim / Design** | | | | **Participants** | | | **Intervention** | | | **Main Findings & Adverse Events** | | |
| Nemett et al (2008)  Dysfunctional Voiding | **Aim:** To determine whether MPT-OA added to standard treatment (ST)improves DV more effectively than ST alone.  **Design:** RCT | | | | n= 21  **Age**: 4-11   **Inclusion Criteria:** (1) Children aged 4-11 years who were consecutive new referrals to an interdisciplinary paediatric urology clinic, specializing in the treatment of children with recalcitrant voiding dysfunction, between 1999 and 2003  (2) Children enrolled in this study had been treated by a paediatric urologist for at least 6 months prior to study entry. | | | Treatment Group (n=10): Standard care from the Urology clinic and four x 1 hour customised manual physical therapy-Osteopathic Approach (MPT-OA) treatments (gentle mobilisations of body tissues to relieve movement restrictions to achieve balanced alignment and mobility and postural symmetry, with particular attention to the thoracolumbar spine, thoracic and pelvic diaphragms, pelvis, pelvic organs, and lower extremities. Treatments coincided with Urology clinic appointments.  Control Group (n=11): Standard care from Urology clinic (medications, establishment of timed voiding and evacuation schedules, dietary modifications, behaviour modification, pelvic floor muscle retraining, biofeedback training, and treatment of constipation (1 hour every 2 weeks).  **Context**: Physical Therapy - Osteopathic  **Outcomes**: Symptom improvement or resolution pre and post intervention | | | **Main Findings**: MPT-OA treatment can improve short-term outcomes in children with dysfunctional voiding, beyond improvements observed with standard treatments (Z = -2.63, p=0.008).  **Adverse Events**: Not reported. | | |
| Olafsdottir et al (2001)  Infantile Colic | **Aim:** To investigate the efficacy of chiropractic spinal manipulation in the management of infantile colic.  **Design:** Randomised blind, placebo-controlled trial | | | | n= 86   **Age:** infants aged 3-9 weeks  **Inclusion Criteria:**  (1) Infantile colic with minimum of three hours of crying per day, three days per week for the last three weeks (2) Aged 3–9 weeks (3) No benefit from cow’s milk free diet to the mother for four days in breast fed infants, or casein hydrolysed formula for four days in bottle fed infants (4) No signs of lactose intolerance,  (5) No previous chiropractic treatment  (6) Appropriate gain in weight, length, and head circumference and a normal psychomotor development on paediatric physical examination (7) Born at term with a birth weight of more than 2500g  (8) Written informed consent from the parents before entering the study | | | Treatment (n= 31): High velocity, short lever adjustments of the spine consistent with the Palmer Package Techniques.  Control (n = 15): a sham adjustment using an Activator at a non-tension setting administered to the examiner's underlying contact point.  **Context**: Chiropractic   **Outcomes**: 24h crying diary completed by parent and parent report of effect after last visit (8–14 days later) | | | **Major Findings:** Chiropractic spinal manipulation is no more effective than placebo for symptom management of infantile colic (p=0.743). Further chiropractic spinal manipulation is no more effective than placebo for reducing crying time (day 3-6 post treatment: p=0.602) and Day 8 post treatment: p=0.982). This study emphasises the need for placebo controlled and blinded studies when investigating alternative methods.  **Adverse Events**: Not reported. | | |
| Reed et al (1994)  Nocturnal Enuresis | **Aim:** To evaluate chiropractic management of primary nocturnal enuresis in children.  **Design:** RCT | | | | n= 46  **Age**: 5-13 years  **Inclusion Criteria**: not mentioned | | | Lumbar manipulation + exercise (n= 17): patient was passively side- bent away from the therapist. The therapist passively rotated the thoracic spine and then delivered a quick posterior and inferior thrust through the anterior superior iliac spine.   Sham manipulation + exercise (n=17): equal and opposite force was applied to the lumbar spinous process with no physiologic motion   Duration: All patients performed 4 weeks of physical therapy exercise. Follow up: 4 weeks and 6 months  **Context:** Physiotherapy, manual therapists and certified orthopaedic clinical specialists certified in manual therapy  **Outcomes:** Frequency of wet nights | | | **Main Findings**: There was no significant different in the mean pre-to post-treatment change in wet night frequency between the treatment and sham treatment groups (p=0.067).   **Adverse Events**: Not reported. | | |
| **Study** | **Aim / Design** | | | | **Participants** | | | **Intervention** | | | **Main Findings & Adverse Events** | | |
| Selhorst &  Selhorst (2015)  Low back pain | **Aim: (1) to assess the efficacy of** adding lumbar manipulation to an exercise program in adolescents with acute (<90 days) LBP and (2) to report and assess any adverse reactions associated with lumbar manipulation noted in this study.  **Design:** RCT | | | | n = 34  **Age**:13-17 years  **Inclusion Criteria:**  (1) 13-17 years old (2) Mechanical LBP (3) Duration of symptoms <90 days | | | Manipulation & Exercise (n=17): passive rotation of the thoracic spine and then a quick posterior and inferior thrust through the anterior superior iliac spine was applied  Sham & Exercise (n=17): equal and opposite force was applied to the lumbar spinous process with no physiologic motion   Exercise in both groups consisted of lumbar stabilization, range of motion, postural training, core strengthening, and stretching. Duration: 4 weeks of exercise with only 2 sessions of manipulation or sham manipulation  Follow Up: 1 week, 4 weeks, 6 months  **Context**: Chiropractic  **Primary Outcomes:** patient-specific functional scale (PSFS) and numeric pain-rating scale (NPRS).  Secondary Outcome Measure: global rating of change (GROC) scale to assess perceived improvement | | | **Main Finding**s: Addition of lumbar manipulation to an exercise program did not benefit adolescents with acute low back pain. No statistical or clinical differences were noted for pain (p=0.369), function (p=0.143), or global rate of change/perceived improvement (GROC) scale (p=0.938) between groups. Adolescents with LBP had a high frequency of chronic or recurrent symptoms at the 6-month follow-up.  **Adverse Events**: No increased risk of experiencing a mild to moderate adverse reaction with adding lumbar manipulation to an exercise program noted in this study. | | |
| Wiberg et al (1999)  Infantile Colic | **Aim:** To determine whether there is a short-term effect of spinal manipulation in the treatment of infantile colic.  **Design:** RCT | | | | n= 50   **Age**: infants  **Inclusion Criteria:**  (1) Aged 2 to 10 weeks (2) No symptoms that could be a sign of any other disease than infantile colic (3) 1 or more violent spells of crying per day lasting at least 3 hours long per day and must have been present at least 5 of the 7 previous days (4) Normal behaviour (5) Must not suffer from any known past or present disease (6) Average weight gain of at least 150 g/week (7) Show typical colic behaviour during the spells of crying (i.e., motor unrest; often flexing knees against the abdomen; or extending the trunk, neck, and extremities)  (8) During attacks the infant cannot (or only temporarily) be comforted by (a) being picked up, walked, or cradled; (b) change of diaper; (c) being offered a dummy | | | Dimethicone group (n=20): dimethicone daily for 2 weeks as prescribed plus advice and education on breastfeeding techniques and mother's diet   Manipulation Group (n=25): spinal manipulation/mobilisation for 2 weeks (3-5 treatment sessions) with specific light pressure with the fingertips plus advice and education on breastfeeding techniques and mother's diet   **Context**: Chiropractic  **Primary Outcomes**: percent change within each child of the mean hours of infantile colic behaviour per day as registered in the diaries | | | **Main Findings**: Spinal manipulation is effective in relieving infantile colic symptoms in the medium term (4-7 days) (p=0.04) and (days 8-11) (p=0.004) but not the immediate to short term (0-3 days) (p=0.37).  **Adverse Events**: Not reported. | | |
| **Data Extraction for Other Studies** | | | | | | | | | | | | | |
| **Study** | | | **Aim / Design / Location** | | | **Participants** | | **Intervention** | | **Main Findings & Adverse Events** | | | |
| Alcantara et al (2009) | | | **Aim:** To describe the practice of paediatric chiropractic, including its safety and effectiveness  **Design:** Survey  **Location:** Atlanta, USA | | | n = 577 infants, children and adolescents   **Age**: Birth- 19 years   **Inclusion Criteria:**  (1) Chiropractor must be in good standing with Board of Chiropractic Examiners in his/her state (2) Must agree to the terms of participation as an ICPA PBRN participant  (3) Subject of interest must have received SMT care ranging from one to 12 visits. | | Full spine manipulation and regional spinal care.  **Context**: Chiropractic   **Outcomes**: decreased pain, changes in mood, immune function, sleep patterns | | **Statistics:** Data was entered in a Portable Document Format (PDF) through Adobe Reader. From this PDF, an Extensible Mark-up Language (XML) file was created containing the data entered in the original form and analysed using descriptive synthesis.  **Main Findings:** - With respect to condition-based presentations, MSK conditions were the most common, in addition to non MSK conditions of childhood. The most common techniques used were: diversified technique, Gonstead technique, Thompson technique, and activator methods. - With respect to the spinal regions addressed, regardless of clinical presentation, 77 patients received full spine SMT care, whereas 500 patients received regional spinal care. Full spine care denotes that SMT was applied to the cervical, thoracic, and lumbosacral spine at each visit. Regional care denotes the patient receiving SMT at one or two spinal regions. (i.e., cervical and thoracic spine or thoracic and lumbosacral spine). - Parent surveys demonstrate high perceived effectiveness (162 of 239 parents reported treatment-related improvements) for paediatric chiropractic care as well as a high level of safety (2 of 239 parents reported treatment-associated aggravations and none reported treatment-associated complications).  **Adverse Events**:  - Two separate reports of treatment-associated aggravation. These were reported as “soreness of the knee” and “cervical spine stiffness”. - Reported aggravations (from chiropractor and parent survey) were minor, self-limiting, and did not require hospitalisation or medical attention. | | | |
| Davies &  Jamison (2007)  Infantile Colic | | | **Aim**: To undertake a study describing chiropractic management and clinical outcome of a common early life problem.   **Design**: Longitudinal, practice based naturalistic study   **Location**: Australia | | | n=52 infants  **Age:** 1-43 weeks (Average – 7 weeks)  **Inclusion Criteria:**  (1) All babies presenting to the 3 chiropractic clinics who had loud, continuous, inconsolable crying for hours were included.  (2) Babies presenting with at least 2 of the following were also included: a flushed face with or without circumoral pillow, clenched fists, legs pulled up to abdomen, breast refusal, cold feet, a distended abdomen. | | Spinal manipulative therapy- details not reported.  **Context:** Chiropractic  **Outcome**: Crying time | | **Main Findings:** More than half of the babies improved ‘somewhat’ after one adjustment. In most cases, carers felt that the encounter was beneficial. Whether such benefit is attributable to reduction in psychological stress or physiological change requiring further investigations. Chiropractic care provides a potentially useful alternative for management of irritable babies. This study neither confirms nor refutes a cause-effect relationship between chiropractic subluxation and irritable baby syndrome.  **Adverse Events:** Not reported. | | | |
| **Study** | | | **Aim / Design / Location** | | | **Participants** | | **Intervention** | | **Main Findings & Adverse Events** | | | |
| Hayden et al (2003)  Low back pain | | | **Aim**: To describe chiropractic management of LBP in patients between the ages of 4 and 18 years, as well as outcomes and factors associated with the outcomes.   **Design:** Prospective cohort study  **Location**: Canada | | | n= 15 chiropractors  n=54 paediatric clients   **Age**: 2-17 years   **Inclusion Criteria:** (1) Practitioners were eligible to participate if they had been in clinical practice for more than 5 years, and if they saw, on average, a minimum of 2 paediatric patients per week. (2) 4 and 18 years with a new episode of mechanical LBP not previously treated by a chiropractor | | **Intervention:** Spinal Manipulation / Adjustment  **Context:** Chiropractic  **Outcomes**: Low Back Pain, paediatric visual analogue scale (VAS) | | **Statistics**: Used descriptive statistics to describe study subjects, assessment, and management. Kaplan-Meier curves produced to estimate time and number of follow-up visits to important outcomes. The X2 test for categorical data used for single-variable comparisons between groups. Crude relative risks (RRs) and 95% CIs were computed for various demographic and condition-related characteristics.   **Main Findings**: Almost 90% of cases presented with uncomplicated mechanical LBP, most frequently diagnosed as lumbar facet dysfunction or subluxation. Patients were managed with manipulation, with a minority (7.7%) receiving some form of active management. “Important” improvement was seen in 53.7% on the visual analogue (VAS) and patients subjectively reported being ‘improved’ (92.35), ‘much improved’ (78.9%) and ‘resolved’ (47.2%) within a 6-week course of management. Patients with chronic LBP were less likely to respond positively within the median number of treatments.  The majority of the patients presented with localised LBP of acute onset. 24% presented with chronic pain longer than 3 months. Majority of patients were managed with manipulation/ adjustment, but relatively few were provided with active care strategies. Patients responded favourably to chiropractic management, and there were no reported complications. Future investigations should establish the natural history and compare chiropractic management to other forms of treatment to gain knowledge about the effectiveness of chiropractic in managing paediatric LBP.  **Adverse Events:** Not reported. | | | |
| **Study** | | | **Aim / Design / Location** | | | **Participants** | | **Intervention** | | **Main Findings & Adverse Events** | | | |
| Lantz & Chen (2001)  Scoliosis | | | **Aim:** To assess the effectiveness of chiropractic intervention in the management of adolescent idiopathic scoliosis in curves less than 20°  **Design**: Cohort time-series trial with all subjects electing chiropractic care.  **Location:** Not reported. | | | n= 42   **Age**: 6-12 years  **Inclusion Criteria**: participants 6 to 12 years with entry-level x-ray films revealing curves of 6° to 20° | | Intervention (n=42): spinal manipulation including high velocity, low amplitude thrust with heel lifts, postural and lifestyle counselling  Duration: 1 year before follow-up  **Context:** Chiropractic   **Outcomes**: cobb angle measurement (pre and post intervention) | | **Main Findings:** Full-spine chiropractic adjustments with heel lifts and postural and lifestyle counselling are not effective in reducing the severity of scoliotic curves. Curve changes were not associated with frequency of chiropractic care (r^2^ = 0.05).  **Adverse Events:** Not reported. | | | |
| Leboeuf et al (1991)  Nocturnal Enuresis | | | **Aim**: To assess the magnitude of response to chiropractic care in the treatment of enuresis, comparing this to the treatment response with other therapies reported in the literature, as well as attempting to identify predictive factors for treatment   **Design**: Prospective outcome study  **Location:** not mentioned | | | n= 171   **Age**: 4-15 years  **Inclusion Criteria**: not mentioned | | Group A (n=71): received no treatment for 2 weeks and then received chiropractic treatment.  Group B (n=100): received chiropractic adjustments of the area(s) of aberrant spinal movement directly following the second visit.  **Context**: Chiropractic   **Outcomes:** number of wet nights | | **Main Findings**: Results do not support the claim that chiropractic care in enuretic children is an effective therapy for this condition. After 2 weeks without therapy, the number of wet nights decreased significantly from 7.0 to 5.6 (p=0.01) and by the end of the treatment period this number reduced to 4.0 (p=0.0001). After chiropractic treatment, 15.5% of subjects wet a maximum of 2 nights per fortnight or 1 night/week. These results are less favourable than other common types of therapy, which have reported ‘cure’ rates well above 50%. In the absence of a control group there is no validity to the claim that chiropractic is a treatment of choice for functional nocturnal enuresis.   **Adverse Events**: Two children complained of ill-effects from chiropractic treatment, one child developed severe headaches and a stiff neck after cervical spine treatment and one child developed acute pain in the lumbar spine. | | | |
| **Study** | | | **Aim / Design / Location** | | | **Participants** | | **Intervention** | | **Main Findings & Adverse Events** | | | |
| Miller & Newell (2012)  Infantile Colic | | | **Aim:** To determine any possible justification of the use of three a priori clinically determined categories of excessively crying infants, based on differences in parent reported outcomes after a course of chiropractic treatment.  **Design**: prospective observational cohort study  **Location:** United Kingdom | | | n= 158  **Age**: 0-18 weeks  **Inclusion Criteria:**  (1) All babies between the ages of one day and 18 weeks who presented between July 2007 and March 2008 with the chief complaint of excessive crying were eligible  (2) Included if infant could be categorised using clinical signs and symptoms into one of the three classification groups; infant colic, irritable Infant syndrome of musculoskeletal origin (IISMO) or inefficient feeding crying infant with disordered sleep (IFCIDS) | | Infantile colic (n=77): chiropractic treatment (manipulation) to spinal area of dysfunction  Irritable infant syndrome of musculoskeletal origin (n=56): chiropractic treatment (manipulation) to spinal area of dysfunction.  Inefficient feeding crying infant with disordered sleep (n=21): chiropractic treatment (manipulation) to spinal area of dysfunction.  **Context**: Chiropractic  **Outcomes**: Changes in continuous outcomes (crying, stress, sleep, and consolability) were assessed by parent reported outcomes after a course of chiropractic treatment. | | **Main Findings:** Colic babies accounted for 77 (49%) of the participant group. The area of the spine treated was not associated with outcomes. Infants with colic were reported by parents to improve significantly (p=<0.001) with treatment in crying time, sleep, stress, and consolability. However, the ‘condition’ was the only variable associated with change scores, with the exception of ‘number of treatments’ being weakly associated with increased sleep and decreased stress scores. Infants classified as having colic required significantly less treatments than infants with other conditions associated with excessive crying (p=<0.001).  **Adverse Events:** Not reported. | | | |
| Miller &  Benfield (2008)  Adverse Events | | | **Aim**: To identify any adverse effects to chiropractic care occurring in the paediatric patient and to evaluate the risk of complications arising in the paediatric patient resulting from chiropractic care  **Design**: Retrospective study   **Location**: Bournemouth, England | | | n= 697  **Age**: 0-3 years  **Inclusion Criteria**: all files of paediatric patients younger than 3 years of age were selected manually in sequential order from current files stored in the AECC clinic presenting to the AECC clinic during a specific period. | | Intervention: retrospectively looked at 697 paediatric patient files for adverse effects  **Context:** Chiropractic   **Outcomes**: parental comments on improvement, no change, or worsening of child's symptoms. | | **Main Findings**: 697 children received a total of 5242 chiropractic treatments, with 85% of parents reporting an improvement in presenting symptoms. Seven parents reported an adverse effect. There was a reaction rate of approximately 1 child in 100, or one reaction reported for every 749 treatments. There were no serious complications resulting from chiropractic treatment (i.e., reactions lasting >24 hours or severe enough to require hospital care). This study shows that chiropractic manipulation produced very few adverse effects and was a safe form of therapy in the treatment of patients in this age group.  **Adverse Events**: All 7 adverse reactions to care were mild/transient in nature and required no medical care. | | | |
| Miller & Phillips (2009)  Infantile Colic | | | **Aim**: To document any behavioural or sleep disturbances experienced by post-colicky toddlers who were previously treated with chiropractic care vs those who had not experienced this treatment as an infant.  **Design:** Survey comparison post intervention  **Location**: England | | | n= 50 parents with toddlers in a treatment group and 45 toddlers in the nontreatment group   **Age**: parents and toddlers  **Inclusion Criteria for the nontreatment group:** (1) Child 2 to 3 years of age (2) Parents reported that child had experienced infant colic (3) Child had never been treated with chiropractic manual therapy | | Treatment group (n=50): if they had been treated for infant colic with routine low-force chiropractic manual therapy.   Nontreatment group (n=45): consisted of post-colicky children in the same age group who had received no chiropractic care for their diagnosed colic as infants.   **Context**: Chiropractors  **Outcomes:** decreased symptoms (temper tantrums, crying and nocturnal waking) from parental report from survey and crying diary | | **Main Findings**: Untreated post-colicky infants demonstrated negative behavioural patterns at 2 to 3 years of age. Toddlers who were treated with chiropractic care for colic were twice as likely to not experience long-term sequelae of infant colic, such as temper tantrums (relative risk, 2.0; 95% confidence interval, 1.3-3.0) and frequent nocturnal waking (relative risk, 2.0; 95% confidence interval, 1.5-2.8) than those who were not treated with chiropractic care as colicky infants.   **Adverse Events**: Not reported. | | | |
| **Study** | | | **Aim / Design / Location** | | | **Participants** | | **Intervention** | | **Main Findings & Adverse Events** | | | |
| Saedt et al (2018)  Upper Cervical Dysfunction | | | **Aim**: To describe common clinical practices of manual therapists (MTs) in the Netherlands for infants with indications of upper cervical dysfunction (UCD).  **Design:** prospective observational cohort design  **Location:** Netherlands | | | n= 307  **Age**: < 27 weeks  **Inclusion Criteria:** (1) infants aged <27 weeks  (2) Referred with indications of UCD and without causative concomitant pathology, potential underlying pathology, and/or red flags in the referral information or history taking | | Treatment group (n=307): mobilisation techniques (traction, lateral flexion, anterior-posterior mobilisation to the cervical spine (C1-C2), and sacroiliac mobilisation).  **Context**: Manual therapists  **Outcomes**: parents reported on infant characteristics and perceived effect of treatment pre and post intervention  Active, spontaneous, and provoked mobility and passive upper cervical mobility using the flexion-rotation test (FRT) and lateral flexion test (LFT) are used. | | **Main Findings**: This is the first study to describe common clinical practice for infants referred for manual therapy. Infants with UCD were treated mainly with upper cervical mobilisation techniques and after treatment, positive diagnostic outcomes on the flexion-rotation test decreased from 78.8% to 6.8%. For lateral flexion test, the positive diagnostic outcomes decreased from 91.5% to 6.2%. All parents perceived positive treatment effects and the greatest perceived effect was observed after approximately 2 treatment sessions.  **Adverse Events:** No serious events reported. | | | |
| Sawyer et al (1999)  Otitis Media | | | **Aim:** To assess the feasibility of conducting a full-scale randomised clinical trial investigating the efficacy of chiropractic spinal manipulative therapy (SMT) for children with chronic otitis media with effusion.  **Design**: Prospective, parallel-group, observer-blinded, randomised feasibility study  **Location**: Bloomington, Minnesota | | | n= 20 patients  **Age:** 6 weeks-6 months   **Inclusion Criteria:**  (1) 6 months to 6 years (2) Three or more episodes of acute otitis media in the previous year (3) Middle ear effusion (as determined by tympanometry) at the first two evaluation visit | | Active chiropractic SMT (n=9): low amplitude, high-velocity manual spinal manipulation   Placebo chiropractic SMT (n=11): static and motion palpation and light touch of specific spinal segments so that the placebo treatment was identical to the active treatment except for the low-amplitude, high-velocity thrust.   Duration: 4 weeks with 1 month follow up  **Context**: Chiropractic   **Outcomes:** otitis media–related patient symptoms, sleep patterns, need for medical care, and medications as recorded by the parents in a daily diary | | **Main Findings:** Recruitment for a randomised controlled trial is feasible and could be enhanced by medical collaboration. Patients and parents are able and willing to participate in a study comparing active SMT and placebo SMT. Parents were extremely compliant with the daily diaries, suggesting that similar quality-of-life and functional status measures can be successfully used in a larger trial. Effectiveness of SMT was not assessed in this feasibility study and because of this and the limited sample, the data must be interpreted with extreme caution, and no conclusions regarding the efficacy of SMT for OME can be drawn.   **Adverse Events**: No reports of serious side effects as a result of either the active or placebo chiropractic treatments. One parent reported their child had some mid-back soreness after one treatment that resolved after a few days, and another child was reported by the parent as being irritable for a short time after treatment. One parent of a child in the placebo chiropractic group reported excessive crying by the child after treatment. | | | |
| Zhang &  Snyder (2004)  Otitis Media | | | **Aim:** To study the effect of Toftness chiropractic adjustment for acute otitis media in children.  **Design**: cohort study  **Location**: not mentioned | | | n= 21 infants and children  **Age**: 0-10 years  **Inclusion Criteria:**  (1) Child had acute otitis media for less than  (2) Children under the age of 10 | | **Intervention** (n=21): low force Toftness chiropractic adjustment by a metered hand-held pressure applicator at the cervical, thoracic, lumbar and sacral contact site. This applicator is a rubber-tipped, spring-loaded device that indicates the amount of force that is being applied at the contact site.   **Context:** Chiropractic   **Outcomes**: tympanic membrane redness and oral temperature post SMT | | **Main Findings:** Consistency of the results provides evidence that patients with otitis media may benefit from the Toftness chiropractic adjustment. This study reported that after Toftness chiropractic adjustment, the red and bulging tympanic membrane associated with acute otitis media, returned to normal in 95% of the children with a significant reduction in symptoms (p<0.01) and a decrease in average oral temperature from 100 degrees F to 98.6 degrees F (p<0.01). The small number of subjects and the cohort study design limit the conclusions that can be made from this data; however, this data is clearly strong enough to justify a larger, more well-controlled clinical trial to determine the extent of efficacy of Toftness chiropractic adjustment at treating this widespread childhood condition.  **Adverse Events**: Not reported. | | | |
