# Supplementary material for: Spinal manipulation and mobilisation in the treatment of infants, children, and adolescents: a systematic scoping review
Source: BMC Pediatr. 2022 Dec 19;22:721. doi: 10.1186/s12887-022-03781-6 (PMC9762100; doi:10.1186/s12887-022-03781-6)
Supplement: Supplementary file 4 — Additional file 4: Supplementary File 4. Matrix with RCT's and other studies included across all systematicreviews [file 12887_2022_3781_MOESM4_ESM.docx]

| **SUPPLEMENARY FILE 4** Matrix with RCT's and other studies included across all systematic reviews | | | | | | | | | | | | | | | | | | | | | | | | | | | | | | | | | | | |
| --- | --- | --- | --- | --- | --- | --- | --- | --- | --- | --- | --- | --- | --- | --- | --- | --- | --- | --- | --- | --- | --- | --- | --- | --- | --- | --- | --- | --- | --- | --- | --- | --- | --- | --- | --- |
|  | Reviews | | | | | | | | | | | | | | | | | | | | | | | | | | | | | | | | | | |
| Studies | Alcantara, J, Alcantara, J. D., Alcantara, J.(2011a) (Level 1b) | Alcantara, J, Alcantara, J. D., Alcantara, J. (2011b) (Level 1b) | Alcantara, J, Alcantara, J. D., Alcantara, J.(2015) (Level 2b) | Brand et al. (2005) (Level 1a) | Bronfort et al. (2010) (Level 1a) | Brurberg, K., Dahm, K., Kirkehei, I. (2019) (Level 1b) | Carnes et al. (2018) (Level 1b) | Corso et al. (2020) (Level 1b) | Clar et al. (2014) (Level 1b) | Dobson et al. (2012) (Level 1a) | Driehuis et al. (2019) (Level 1b) | Edwards, C., Miller, J. (2019) (Level 3b) | Ellwood, J., Draper-Rodi, J., Carnes, D. (2020) (Level 1b) | Ernst (2009) (Level 1a) | Fairest, C., Chiro, B., Russell, D. (2019) (Level 4) | Ferrance, R., Miller, J. (2010) (Level 1b) | Fry (2014) (Level 3b) | Glazener, C., Evans, J., Cheuk, D. (2005) (Level 1a) | Gleberzon et al. (2012) (Level 1b) | Green et al. (2019) (Level 1b) | Hawk et al. (2007) (Level 1b) | Hawk et al. (2019) (Level 1b) | Hondras, M., Linde, K., Jones, A. (2005) (Level 1a) | Humphreys (2010) (Level 1b) | Huang, Huang and Cheuk (2011) (Level 1a) | Karpouzis et al., Karpouzis, F., Bonello, R., Pollard, H. (2010) (Level 2a) | Kronau, S., Thiel, B., Jäkel, A., Liem, T (2016) (Level 2b) | Lucassen (2010) (Level 1b) | Parnell Prevost et al. (2019) (Level 1b) | Pohlman, K., Holton-Brown, M. (2012) (Level 1b) | Romano, M., Negrini, S. (2008) (Level 3b) | Theroux et al. (2017) (Level 1b) | Todd et al. (2005) (Level 1b) | Vaughn, D, Kenyon, L., Sobeck, C., Smith, R (2012), (Level 1b) | Vohra et al. (2007) (Level 1b) |
| **RCT’s** | | | | | | | | | | | | | | | | | | | | | | | | | | | | | | | | | | | |
| Balon et al (1998) | N | N | N | N | Y | N | N | N | N | N | N | N | N | N | N | N | N | N | Y | Y | Y | N | Y | N | N | N | N | N | N | N | N | N | Y | N | N |
| Borusiak (2009) | N | N | N | N | N | N | N | N | N | N | Y | N | N | N | N | N | N | N | N | Y | N | N | N | N | N | N | N | N | Y | N | N | N | N | Y | N |
| Bronfort et al (2001) | N | N | N | N | N | N | N | Y | N | N | Y | N | N | N | N | Y | N | N | Y | Y | Y | N | Y | N | N | N | N | N | Y | Y | N | N | Y | N | N |
| Browning and Miller (2008) | N | Y | N | N | Y | N | Y | N | N | N | Y | N | N | Y | N | Y | N | N | Y | Y | N | N | N | N | N | N | N | N | Y | N | N | N | N | N | N |
| Cabrera- Martos (2016) | N | N | N | N | N | Y | N | N | N | N | N | N | N | N | N | N | N | N | N | Y | N | N | N | N | N | N | N | N | Y | N | N | N | N | N | N |
| Dissing et al (2018) | N | N | N | N | N | N | N | N | N | N | N | N | N | N | N | N | N | N | N | Y | N | N | N | N | N | N | N | N | N | N | N | N | N | N | N |
| Evans (2018) | N | N | N | N | N | N | N | N | N | N | N | N | N | N | N | N | N | N | N | Y | N | N | N | N | N | N | N | N | Y | N | N | N | N | N | N |
| Haugen et al (2011) | N | N | N | N | N | Y | N | N | N | N | Y | N | Y | N | N | N | N | N | N | Y | N | N | N | N | N | N | N | N | Y | N | N | N | N | N | N |
| Kachmar, (2018) | N | N | N | N | N | N | N | N | N | N | N | N | N | N | N | N | N | N | N | Y | N | N | N | N | N | N | N | N | N | N | N | N | N | N | N |
| Lynge et al (2021) | N | N | N | N | N | N | N | N | N | N | N | N | N | N | N | N | N | N | N | N | N | N | N | N | N | N | N | N | N | N | N | N | N | N | N |
| Miller, Newell and Bolton (2012) | N | N | N | N | N | N | Y | Y | Y | Y | Y | N | N | N | N | N | N | N | N | Y | N | N | N | N | N | N | N | N | Y | N | N | N | N | N | N |
| Nemett et al (2008) | N | N | N | N | N | N | N | N | Y | N | N | N | N | N | N | N | N | N | N | N | N | N | N | N | N | N | N | N | N | N | N | N | N | N | N |
| Olafsdottir et al (2001) | N | Y | N | Y | Y | N | Y | N | N | Y | Y | N | N | Y | N | Y | N | N | Y | Y | Y | N | N | N | N | N | N | Y | Y | N | N | N | Y | N | N |
| Reed et al (1994) | N | N | N | N | Y | N | N | N | N | N | Y | N | N | N | N | N | N | Y | Y | Y | Y | N | N | N | Y | N | N | N | N | N | N | N | N | N | N |
| Selhorst (2015) | N | N | N | N | N | N | N | N | N | N | N | N | N | N | N | N | N | N | N | Y | N | N | N | N | N | N | N | N | Y | N | N | N | N | N | N |
| Wiberg, Nordsteen and Nilsson (1999) | N | Y | N | Y | Y | N | Y | N | N | Y | Y | N | N | Y | N | Y | N | N | N | Y | Y | N | N | N | N | N | N | Y | N | N | N | N | Y | N | N |
| **Other studies** | | | | | | | | | | | | | | | | | | | | | | | | | | | | | | | | | | | |
| Alcantra, Ohm and Kunz (2009) | N | N | N | N | N | N | N | N | N | N | N | N | N | N | N | N | N | N | N | Y | N | N | N | Y | N | N | N | N | N | Y | N | N | Y | N | N |
| Davies and Jamison (2007) | N | N | N | N | N | N | N | N | N | N | N | N | N | N | N | N | N | N | N | N | N | N | N | N | N | N | N | N | N | N | N | N | N | N | N |
| Hayden, Mior and Verhoef (2003) | N | N | N | N | N | N | N | N | N | N | N | N | N | N | N | N | N | N | N | Y | N | N | N | N | N | N | N | N | Y | N | N | N | Y | N | N |
| Lantz and Chen (2001) | N | N | N | N | N | N | N | N | N | N | N | N | N | N | N | N | N | N | N | Y | N | N | N | N | N | N | N | N | Y | N | Y | Y | N | N | N |
| Leboeuf et al (1991) | N | N | N | N | Y | N | N | N | N | N | N | N | N | N | N | N | N | Y | Y | Y | Y | N | N | Y | N | N | N | N | N | N | N | N | Y | N | Y |
| Miller and Beinfield (2008) | N | N | N | N | N | N | Y | N | N | N | N | N | N | N | N | Y | N | N | Y | Y | N | N | N | Y | N | N | N | N | N | N | N | N | N | N | N |
| Miller and Phillips (2009) | N | Y | N | N | N | N | Y | N | N | N | N | N | N | N | N | N | N | N | N | N | N | N | N | N | N | N | N | N | N | N | N | N | N | N | N |
| Miller and Newell (2012) | N | N | N | N | N | N | N | N | N | N | N | N | N | N | N | N | N | N | N | N | N | N | N | N | N | N | N | N | N | N | N | N | N | N | N |
| Saedt et al (2018) | N | N | N | N | N | N | N | Y | N | N | N | N | N | N | N | N | N | N | N | Y | N | N | N | N | N | N | N | N | Y | N | N | N | N | N | N |
| Sawyer et al (1999) | N | N | N | N | N | N | N | Y | N | N | N | N | N | N | N | N | N | N | Y | Y | Y | N | N | Y | N | N | N | N | N | Y | N | N | Y | N | Y |
| Zhang and Synder (2004) | N | N | N | N | N | N | N | N | N | N | N | N | N | N | N | N | N | N | N | Y | N | N | N | N | N | N | N | N | N | N | N | N | N | N | N |
| Y – The study was captured in the systematic review. N – The study was not captured by the systematic review. | | | | | | | | | | | | | | | | | | | | | | | | | | | | | | | | | | | |
