# Supplementary material for: Spinal manipulation and mobilisation in the treatment of infants, children, and adolescents: a systematic scoping review
Source: BMC Pediatr. 2022 Dec 19;22:721. doi: 10.1186/s12887-022-03781-6 (PMC9762100; doi:10.1186/s12887-022-03781-6)
Supplement: Supplementary file 5 — Additional file 5: Supplementary File 5. Descriptive Synthesis [file 12887_2022_3781_MOESM5_ESM.docx]

| Supplementary File 5 Descriptive Synthesis | | | | | | | | | | | | |
| --- | --- | --- | --- | --- | --- | --- | --- | --- | --- | --- | --- | --- |
| Descriptive Synthesis Exploring Effectiveness of Spinal *Manipulation* on Infants, Children and Adolescents – Reviews and Studies | | | | | | | | | | | | |
| Conditions | **Outcomes for defined impairments** | | **Significant Positive (Favourable) Effect (+)** | **No significant effect (0)**  **Significantly negative (Unfavourable) Effect (-)** | | **Inconclusive (Inc)** | | **Level of Evidence Statement** | | | |  |
|  |  | | **(Authors, Year, Type, n = sample size / demographic)** | | | | |  | | | |  |
| Adolescent Idiopathic Scoliosis (AIS) | 1. Cobb angle measurement / reduction in scoliotic curve | | - | 1. Lantz (2001) OS (n=42 C&A) (Level III-2) (0) | | 1. Parnell (2019) SR (n=79 C&A) (Level 1b) (Inc)  1. Romano (2008) SR (n=61 A) (Level 3b) (Inc)  1. Theroux (2017) SR (n=234 C+A) (Level 1b) (Inc) | | Inconclusive | | Inconclusive | |  |
|  | 2. Scoliosis Quality of Life Index | | - | - | | 2. Parnell (2019) SR (n=79 C&A) (Level 1b) (Inc). | | Insufficient | |  |  |  |
|  | 3. Pain intensity, physical disability, quality of life, and adverse events | | - | - | | 3. Theroux (2017) SR (n=234 C&A) (Level 1b) (Inc) | | Insufficient | |  |  |  |
| Asthma | 1. Peak expiratory flow | | 1. Hawk (2007) SR (n=270 C&A) (Level 1b) (+) | 1. Gleberzon (2012) SR (n=116 C&A) (Level 1b) (0)  1. Green (2019) SR (n=125 C&A) (Level 1b) (0)  1. Balon (1998) RCT (n=80 C&A) (Level II) (0) | | 1. Parnell (2019) SR (n=174 C&A) (Level 1b) (Inc) | | Inconclusive | | SR = Inconclusive  Studies = STRONG Evidence of No Significant Effect* | |  |
|  | 2. Pulmonary Function | | - | 2. Gleberzon (2012) SR (n=36 C&A) (Level 1b) (0)  2. Bronfort (2010) SR (n= NR) (Level 1a) (-)  2. Hondras (2005) SR (n=91 C&A) (Level 1a) (0)  2. Bronfort (2001) RCT (n=36 C&A) (Level II) (0) | | - | | VERY STRONG Evidence of No Significant Effect (unfavourable) | |  |  |  |
|  | 3. Symptoms | | - | 3. Bronfort (2010) SR (n= NR) (Level 1a) (-)  3. Bronfort (2001) RCT (n=36 C&A) (Level II) (0) | | 3. Ferrance (2010) SR (n=NR I) (Level 1b) (Inc)  3. Parnell (2019) SR (n=174 C&A) (Level 1b) (Inc) | | Inconclusive | |  |  |  |
|  | 4. Asthma Severity | | 4. Bronfort (2001) RCT (n=36 C&A) (Level II) (+) | 4. Bronfort (2001) RCT (n=36 C&A) (Level II) (0) | | 4. Parnell (2019) SR (n=174 C&A) (Level 1b) (Inc) | | Inconclusive | |  |  |  |
|  | 5. Quality of Life (associated with Asthma) | | - | - | | 5. Ferrance (2010) SR (n=NR I) (Level 1b) (Inc)  5. Hondras (2005) SR (n=91 C&A) (Level 1a) (Inc)  5. Parnell (2019) SR (n=174 C&A) (Level 1b) (Inc) | | Inconclusive | |  |  |  |
| Autism Spectrum Disorder (ASD) | 1. Autism related symptoms | | 1.Kronau (2016) SR (n=52 C&A) (Level 2b) (+) | - | | - | | Insufficient | | Inconclusive | |  |
|  | 2. ATEC | | - | - | | 2. Kronau (2016) SR (n=52 C&A) (Level 2b) (Inc) Parnell (2019) SR (n=63 C) (Level 1b) (Inc) | | Inconclusive | |  |  |  |
|  | 3. X-rays | | - | - | | 3. Kronau (2016) SR (n=52 C&A) (Level 2b) (Inc) | | Insufficient | |  |  |  |
|  | 4. Spinal palpation | | - | - | | 4. Kronau (2016) SR (n=52 C&A) (Level 2b) (Inc) | | Insufficient | |  |  |  |
|  | 5. leg length difference | | - | - | | 5. Kronau (2016) SR (n=52 C&A) (Level 2b) (Inc) | | Insufficient | |  |  |  |
|  | 6. SEMG | | - | - | | 6. Kronau (2016) SR (n=52 C&A) (Level 2b) (Inc) | | Insufficient | |  |  |  |
|  | 7. Thermal Scan | | - | - | | 7. Kronau (2016) SR (n=52 C&A) (Level 2b) | | Insufficient | |  |  |  |
|  | 8. Modified Autism Research Institute Outcomes Survey | | - | - | | 8. Parnell (2019) SR (n=63 C) (Level 1b) (Inc) | | Insufficient | |  |  |  |
| Spinal (Back / Neck) Pain (acute and chronic) | 1. Pain Severity / Intensity | | 1. Parnell (2019) SR (n=239 C&A) (Level 1b) (+) – Mixed acute and chronic  1. Evans (2018) RCT (n=185 C&A) (Level II) (+) – Chronic  1. Hayden (2003) OS (n= 33 C&A) (Level III-3) - Acute (+) | 1. Hayden (2003) OS (n= 21 C&A) (Level III-3) – Chronic (0)  1. Selhorst (2015) RCT (n= 35 A) (Level II) – Acute (0) 1. Dissing (2018) RCT (n283 C&A) (Level II) – mixed acute and chronic (0) | | 1. Vaughn (2012) SR (n= 99 C&A) (Level 1b) (Inc) – Mixed Acute and Chronic | | SR = Inconclusive  RCTs = STRONG Evidence of No Significant Effect | | SR = Inconclusive  Studies = Inconclusive | |  |
|  | 2. Episode length / total number of pain weeks | |  | 2. Dissing (2018) RCT (n=283 C&A) (Level II) – mixed acute and chronic (0) – Pain episode length  2. Dissing (2018) RCT (n=283 C&A) (Level II) – mixed acute and chronic (0) – Total complaint duration | |  | | Insufficient | |  |  |  |
|  | 3. Recurrence of spinal pain | | - | 3.. Dissing (2018) RCT (n=283 C&A) (Level II) – mixed acute and chronic (0) | | - | | Insufficient | |  |  |  |
|  | 4. Function / disability | | 4. Evans (2018) RCT (n=185 C&A) (Level II) (+) – Chronic at 26 weeks and 52 weeks post treatment | 4. Selhorst (2015) RCT (n= 35 A) (Level II) – Acute (0) | | - | | Inconclusive | |  |  |  |
|  | 5. Global perceived effect / caregiver rated improvement / satisfaction | | 5. Dissing (2018) RCT (n=283 C&A) (Level II) – mixed acute and chronic (+)  5. Evans (2018) RCT (n=185 C&A) (Level II) (+) - Chronic at 26 weeks and 52 weeks post treatment | - | | - | | RCTs = STRONG Evidence of a Significant Positive Effect | |  |  |  |
|  | 6. Pain medication use | | - | 5. Evans (2018) RCT (n=185 C&A) (Level II) (0) - Chronic | | - | | Insufficient | |  |  |  |
|  | 7. Paediatric Quality of Life (QOL) associated with chronic LBP | | - | 5. Evans (2018) RCT (n=185 C&A) (Level II) (0) - Chronic | | - | | Insufficient | |  |  |  |
| Breastfeeding Difficulty | 1. Biomechanical changes to upper cx spine | | 1. Fry (2014) SR (n=177 I) (Level 3b) (+) | - | | - | | Inconclusive | | Inconclusive | |  |
|  | 2. Improvement in breastfeeding (ability to latch, suck etc.) | | 2. Fry (2014) SR (n=177 I) (Level 3b) (+)  2. Hawk (2019) SR (n=339 I) (Level 1b) (+) | - | | 2. Parnell (2019) SR (n=139 I) (Level 1b) (Inc) | | Inconclusive | |  |  |  |
|  | 3. Improved Breastfeeding | | - | 3. Edwards (2019) SR (n=239 I) (Level 3b) (0) | | - | | Insufficient | |  |  |  |
|  | 4. Exclusive Breastfeeding | | - | 4. Edwards (2019) SR (n=239 I) (Level 3b) (0) | | - | | Insufficient | |  |  |  |
|  | 5. Extended Breastfeeding | | - | 5. Edwards (2019) SR (n=239 I) (Level 3b) (0) | | - | | Insufficient | |  |  |  |
|  | 6. Mother's report of weight gain in infant | | - | - | | 6. Parnell (2019) SR (n=139 I) (Level 1b) (Inc) | | Insufficient | |  |  |  |
| Cerebral Palsy (CP) | 1. Leg/ Hand use during sleep  2. Motor Function  3. Functional Independence  4. Gross motor function measure (GMFMM-66) | | - | 2. Kachmar (2018) RCT (n=78) (C&A) (Level II) (0) – Manual Dexterity | | 1. Clar (2014) SR (n=197 C) (Level 1b) (Inc) 2. Clar (2014) SR (n=197 C) (Level 1b) (Inc) 3. Clar (2014) SR (n=197 C) (Level 1b) (Inc)  4. Parnell (2019) SR (n=247 I&A&C) (Level 1b) (Inc) | | Inconclusive | | Inconclusive | |  |
|  | 5. Quality of life questionnaire (CHQ) | | - | - | | 5.Parnell (2019) SR (n=247 I&A&C) (Level 1b) (Inc) | | Insufficient | |  |  |  |
|  | 6. Muscle spasticity in the wrist muscles | | 6. Kachmar (2018) RCT (n=78) (C&A) (Level II) (+) | - | | - | | Insufficient | |  |  |  |
| Headache | 1. Percentage of days with headache  2. Total duration of headache  3. Days with school absence due to headache | | 1. Lynge (2021) RCT (n=194 C&A) (Level II) (+) | 1. Borusiak (2009) RCT (n=52 C&A) (Level II) (0)  2. Borusiak (2009) RCT (n=52 C&A) (Level II) (0)  3. Borusiak (2009) RCT (n=52 C&A) (Level II) (0) | | 1. Parnell (2019) SR (n=65 I&C&A) (Level 1b) (Inc)  2. Parnell (2019) SR (n=65 I&C&A) (Level 1b) (Inc)  3. Parnell (2019) SR (n=65 I&C&A) (Level 1b) (Inc) | | SR – Insufficient  Studies = Inconclusive | | Reviews: Insufficient  Studies:  STRONG Evidence of No Significant Effect* | | |
|  | 4. Consumption of analgesics | | - | 4. Borusiak (2009) RCT (n=52 C&A) (Level II) (0) 4. Lynge (2021) RCT (n=194 C&A) (Level II) (0) | | - | | STRONG Evidence of No Significant Effect | |  |  |  |
|  | 5. Intensity of headache (i.e., pain) | | - | 5. Borusiak (2009) RCT (n=52 C&A) (Level II) (0)  5. Lynge (2021) RCT (n=194 C&A) (Level II) (0) | | 5. Parnell (2019) SR (n=65 I&C&A) (Level 1b) (Inc) | | STRONG Evidence of No Significant Effect | |  |  |  |
|  | 6. Global perceived effect - parents | | 6. Lynge (2021) RCT (n=194 C&A) (Level II) (+) | - | | - | | Insufficient | |  |  |  |
| Infantile Colic - Excessive Crying | 1. Crying time | | 1. Carnes (2018) SR (n=NR I) (Level 1b) (+)  1. Gleberzon (2012) SR (n=170 I) (Level 1b) (+)  1. Hawk (2007) SR (n=467 I) (Level 1b) (+)  1. Browning (2008) RCT (n=48 I) (Level II) (+) | 1. Bronfort (2010) SR (n=NR) (Level 1a) (-)  1. Dobson (2012) SR (n=325 I) (Level 1a) (0)  1. Green (2019) SR (n=179 I) (Level 1b) (0)  1. Miller & Newell (2012) OS (n = 158 I) (Level III-2) (0)  1. Davies (2007) OS (n=52 I) (Level III-3) (0)  1. Olafsdottir (2001) RCT (n= 86 I) (Level II) (0) | | 1. Clar (2014) SR (n=NR C) (Level 1b) (Inc)  1. Driehuis (2019) SR (n=275 I) (Level 1b) (Inc)  1. Ferrance (2010) SR (n=229 I) (Level 1b) (Inc)  1. Lucassen (2010) SR (n=127 I) (Level 1a) (Inc)  1. Parnell (2019) SR (n=261 I) (Level 1b) (Inc) | | Reviews = Inconclusive  Studies = Inconclusive | | Review = Inconclusive  Studies = Inconclusive | | |
|  | 2. Colic symptoms (temper tantrums, crying, posturing during crying and / or nocturnal waking) | | 2. Gleberzon (2012) SR (n= 973 I & C) (Level 1b) (+)  2. Miller (2009) OS (n= 95 I) (Level III-2) (+)  2. Wiberg (1999) RCT (n=50 I) (Level II) (+) Medium term (4-11 days)  2. Miller & Newell (2012) OS (n = 158 I) (Level III-2) (+) sleep & stress | 2. Ernst (2009) SR (n=198 I) (Level 1a) (-)  2. Wiberg (1999) RCT (n=50 I) (Level II) (0) Short term (0-3 days)  2. Olafsdottir (2001) RCT (n= 86 I) (Level II) (0) Short- term (Day 3-6) and medium-term (Day 8).  2. Miller & Newell (2012) OS (n = 158 I) (Level III-2) (0) crying and consolability. | | - | | Review = Inconclusive  Studies = Inconclusive | |  |  |  |
|  | 3. Prescence/ Absence of colic | | - | 3. Dobson (2012) SR (n=325 I) (Level 1a) (-) | | - | | Insufficient | |  |  |  |
|  | 4. Parent–child relations | | - | - | | 4. Carnes (2018) SR (n=NR I) (Level 1b) (Inc) | | Insufficient | |  |  |  |
| Infantile Colic - Sleep | 1. Sleep time  2. Changes to sleep patterns | | 1. Dobson (2012) SR (n=325 I) (Level 1a) (+)  1. Browning (2008) RCT (n=48 I) (Level II) (+) |  | | 1. Carnes (2018) (n=NR I) (Level 1b) (Inc)  1. Ferrance (2010) SR (n=229 I) (Level 1b) (Inc)  1. Parnell (2019) SR (n=261 I) (Level 1b) (Inc) | | Inconclusive | | Inconclusive | | |
| Nocturnal Enuresis | 1. Number of wet nights  2. Frequency of bed wetting | | - | 1. Glazener (2005) SR (n=217 C&A) (Level 1a) (0)  1. Gleberzon (2012) SR (n=46 C) (Level 1b) (0)  1. Hawk (2007) SR (n=219 I) (Level 1b) (0)  1. Huang (2011) SR (n=46 C&A) (Level 1a) (0) | | 2. Driehuis (2019) SR (n=46 C) (Level 1b) (Inc) | | VERY STRONG Evidence of No Significant Effect | | VERY STRONG Evidence of No Significant Effect | | |
|  | 3. Hours crying, intensity of feeding and stool samples | | - | 3. Gleberzon (2012) SR (n=171 C&A) (Level 1b) (0) | |  | | Insufficient | |  |  |  |
| Otitis Media | 1. Otitis media–related patient symptoms, sleep patterns, need for medical care, and medications as recorded by the parents in a daily diary | | 1. Zhang & Snyder (2004) OS (n=21 I&C) (III-2) (+) | - | | 1. Parnell (2019) SR (n=NR) (Level 1b) (Inc)  1. Pohlman (2012) SR (n=630 I&C) (Level 1b) (Inc) | | Inconclusive | | Review = Inconclusive  Studies = Insufficient | | |
|  | 2. Fewer episodes and recurrences | | - | 2. Hawk (2007) SR (n=465 I&C) (Level 1b) (0) | | - | | Insufficient | |  |  |  |
| Torticollis | 1. Sleep disorders | | - | - | | 1. Brurberg (2019) SR (n=110 I&C) (Level 1b) (Inc) | | Insufficient | | Review = Insufficient  Studies = Insufficient | | |
|  | 2. Duration of crying | | - | - | | 2. Brurberg (2019) SR (n=110 I&C) (Level 1b) (Inc) | | Insufficient | |  |  |  |
|  | 3. Passive cervical flexion and rotation | | - | 3. Haugen et al (2011) RCT (n=32 I) (Level II) (0) | | 3. Brurberg (2019) SR (n=110 I&C) (Level 1b) (Inc) | | Insufficient | |  |  |  |
|  | 4. Degree of cranial symmetry using Argenta Scale | | - | - | | 4. Brurberg (2019) SR (n=46 C) (Level 1b) (Inc) | | Insufficient | |  |  |  |
|  | 5. Spontaneous motor function | | - | - | | 5. Brurberg (2019) SR (n=31 C) (Level 1b) (Inc) | | Insufficient | |  |  |  |
|  | 6. Strength | | - | - | | 6. Brurberg (2019) SR (n=31 C) (Level 1b) (Inc) | | Insufficient | |  |  |  |
|  | 7. Motor development using Alberta Infant Motor Scale | | - | - | | 7. Brurberg (2019) SR (n= 46 C) (Level 1b) (Inc) | | Insufficient | |  |  |  |
|  | 8. Degree of symmetry using standardised video-based measurements | | - | - | | 8. Brurberg (2019) SR (n=32 I) (Level 1b) (Inc) | | Insufficient | |  |  |  |
|  | 9. Head righting reaction | | - | 9. Haugen et al (2011) RCT (n=32 I) (Level II) (0) | | - | | Insufficient | |  |  |  |
| OS: Other Studies; RCT: Randomised Controlled Trial; SR: Systematic Review; NR: Not reported; I: Infant; C: Child; A: Adolescent; -: negative/undesirable effect; +: positive / desirable effect; Inc: inconsistent effects; n: number of participants in the investigation.  The following conditions did not meet methodological requirements for descriptive synthesis of previously published *reviews*: ADHD, Dysfunctional voiding, Kinetic imbalance due to suboccipital strain (KISS), Upper cervical dysfunction.  The following conditions did not meet methodological requirements for descriptive synthesis of individual *studies*: Autism Spectrum Disorder (ASD), Attention Deficit Hyperactivity Disorder (ADHD), Breast feeding difficulties, dysfunctional voiding, Nocturnal Enuresis, KISS Syndrome, Upper cervical dysfunction.  *This outcome may be biased from multiple investigations in a single study or investigations of multiple outcomes in a single review. | | | | | | | | | | | | |
| Descriptive Synthesis Exploring Effectiveness of Spinal *Mobilisation* on Infants, Children and Adolescents – Reviews and Studies | | | | | | | | | | | | |
| Conditions | **Outcomes** | **Significant Positive (Favourable) Effect (+)** | | | **No significant effect (0)**  **Significantly negative (Unfavourable) Effect (-)** | | **Inconclusive (Inc)** | | **Level of Evidence Statement** | |  |  |
|  |  | **(Authors, Year, Type, n= sample size/type)** | | | | | | |  | |  |  |
| Asthma | 1. Peak expiratory flow | - | | | - | | 1. Driehuis (2019) SR (n=106 C&A) (Level 1b) (Inc); | | Insufficient | |  |  |
| ADHD | 1. Outcomes using ADHD Connors scale | - | | | - | | 1. Clar (2014) SR (n=NR C) (Level 1b) (Inc) | | Insufficient | |  |  |
| Infantile Colic | 1. Crying time | 1. Miller, Newell & Bolton (2012) RCT (n=102 I) (Level II) (+) (Medium term – 8-10 days) | | | 1. Miller, Newell & Bolton (2012) RCT (n=102 I) (Level II) (0) Short term (0-6 days) | |  | | Insufficient | |  |  |
| Torticollis | 1. Cervical Mobility 2. Degree of cranial symmetry using Argenta Scale | 1. Ellwood (2020) SR (n=NR I) (Level 1b) (+) 2. Ellwood (2020) SR (n=NR I) (Level 1b) (+) | | | - | | - | | Insufficient | |  |  |
| Plagiocephaly (without torticollis) | 1. Duration of treatment (days)  2. Motor Development | 1. Cabrera-Martos (2016) Pilot RCT (n=46 I) (Level II) (+) | | | 2. Cabrera-Martos (2016) Pilot RCT (n=46 I) (Level II) (0) | | - | | Insufficient | |  |  |
| Upper Cervical Dysfunction | 1. Active, spontaneous, and provoked mobility and passive upper cervical mobility | 1. Saedt (2018) OS (n=307 I) (Level III-2) (+) | | | - | |  | | Insufficient | |  |  |
| OS: Other Studies; RCT: Randomised Controlled Trial; SR: Systematic Review; NR: Not reported; I: Infant; C: Child; A: Adolescent; -: negative/undesirable effect; +: positive / desirable effect; Inc: inconsistent effects; n: number of participants in the investigation.  The following conditions did not meet our methodological requirements for descriptive synthesis of previously published *reviews*: Adolescent Idiopathic Scoliosis (AIS), Autism, Back / Neck Pain, Breastfeeding difficulty, Cerebral Palsy, Dysfunctional voiding, Headache, Infantile colic – crying / behaviour, Infantile colic – sleep, Kinetic imbalance due to suboccipital strain (KISS), Nocturnal Enuresis, Otitis Media, Upper cervical dysfunction.  The following conditions did not meet methodological requirements for descriptive synthesis of individual *studies*: Adolescent Idiopathic Scoliosis (AIS), Asthma, Autism, ADHD, Back / Neck Pain, Breastfeeding difficulty, Cerebral Palsy, Dysfunctional voiding, Headache, Infantile colic – crying / behaviour, Infantile colic – sleep, Kinetic imbalance due to suboccipital strain (KISS), Nocturnal Enuresis, Torticollis. | | | | | | | | | | |  |  |
